# Polymyxin Resistance and Heteroresistance Are Common in Clinical Isolates of *Achromobacter* Species and Correlate with Modifications of the Lipid A Moiety of Lipopolysaccharide

**DOI:** 10.1128/spectrum.03729-22

**Published:** 2022-12-15

**Authors:** Lewis MacDonald, Sean Keenan, Flaviana Di Lorenzo, Nana E. Adade, Dervla T. D. Kenna, Beverley C. Millar, John E. Moore, José Ramos Vivas, Antonio Molinaro, Miguel A. Valvano

**Affiliations:** a Wellcome-Wolfson Institute for Experimental Medicine, Queen's University Belfast, Belfast, United Kingdom; b Department of Chemical Sciences, University of Naples Federico II, Naples, Italy; c Task Force on Microbiome Studies, University of Naples Federico II, Naples, Italy; d West African Center for Cell Biology of Infectious Pathogens, University of Ghana, Accra, Ghana; e Antimicrobial Resistance and Healthcare Associated Infections Unit, UK Health Security Agency, London, United Kingdom; f Laboratory for Disinfection and Pathogen Elimination Studies, Northern Ireland Public Health Laboratory, Belfast City Hospital, Belfast, United Kingdom; g Research Group on Foods, Nutritional Biochemistry and Health, Universidad Europea del Atlántico, Santander, Spain; h CIBERINFEC, Instituto de Salud Carlos III, Madrid, Spain; i Department of Chemistry, School of Science, Osaka University, Osaka, Japan; Emory University School of Medicine

**Keywords:** *Achromobacter*, antibiotic resistance, lipid A, opportunistic infections, polymyxins

## Abstract

The *Achromobacter* genus includes opportunistic pathogens that can cause chronic infections in immunocompromised patients, especially in people with cystic fibrosis (CF). Treatment of *Achromobacter* infections is complicated by antimicrobial resistance. In this study, a collection of *Achromobacter* clinical isolates, from CF and non-CF sources, was investigated for polymyxin B (PmB) resistance. Additionally, the effect of PmB challenge in a subset of isolates was examined and the presence of PmB-resistant subpopulations within the isolates was described. Further, chemical and mass spectrometry analyses of the lipid A of *Achromobacter* clinical isolates enabled the determination of the most common structures and showed that PmB challenge was associated with lipid A modifications that included the addition of glucosamine and palmitoylation and the concomitant loss of the free phosphate at the C-1 position. This study demonstrates that lipid A modifications associated with PmB resistance are prevalent in *Achromobacter* and that subresistant populations displaying the addition of positively charged residues and additional acyl chains to lipid A can be selected for and isolated from PmB-sensitive *Achromobacter* clinical isolates.

**IMPORTANCE** *Achromobacter* species can cause chronic and potentially severe infections in immunocompromised patients, especially in those with cystic fibrosis. Bacteria cannot be eradicated due to *Achromobacter*'s intrinsic multidrug resistance. We report that intrinsic resistance to polymyxin B (PmB), a last-resort antimicrobial peptide used to treat infections by multiresistant bacteria, is prevalent in *Achromobacter* clinical isolates; many isolates also display increased resistance upon PmB challenge. Analysis of the lipopolysaccharide lipid A moiety of several *Achromobacter* species reveals a penta-acylated lipid A, which in the PmB-resistant isolates was modified by the incorporation of glucosamine residues, an additional acyl chain, loss of phosphates, and hydroxylation of acyl chains, all of which can enhance PmB resistance in other bacteria. We conclude that PmB resistance, particularly in *Achromobacter* isolates from chronic respiratory infections, is a common phenomenon, and that *Achromobacter* lipid A displays modifications that may confer increased resistance to polymyxins and potentially other antimicrobial peptides.

## INTRODUCTION

The *Achromobacter* genus is a member of the Gram-negative class *Betaproteobacteria* that belongs to the *Alcaligenaceae* family and currently consists of 36 species, 20 of which are validly published with a correct name (List of Prokaryotic Names with Standing in Nomenclature; https://lpsn.dsmz.de/genus/achromobacter). In addition to Achromobacter xylosoxidans, the type species, the genus includes several other species that can cause opportunistic infections in certain patient populations, such as people with cystic fibrosis (pwCF) (1–6), hematologic and solid organ malignancies, renal failure, and immunodeficiencies (7). Also, increased abundance of A. xylosoxidans has been detected in the upper airways of COVID-19 patients with severe disease and mortality (8). The most frequently isolated species in pwCF is A. xylosoxidans (9–13). Evidence is mounting that pwCF chronically infected with *Achromobacter* are likely to experience pulmonary exacerbations, reduction in respiratory function that may accelerate the need of transplantation, and a greater risk of death (10, 14–18).

Compared to other CF pathogens, the pathogenesis of *Achromobacter* infection and the mechanisms associated with the host inflammatory response are less well understood. The biofilm potential and swimming motility of A. xylosoxidans and an uncharacterized heat-stable cytotoxin have been reported in previous studies (19–23). Genetic analysis of A. xylosoxidans has identified genes encoding a type 3 secretion system (T3SS), a capsular polysaccharide similar to that produced in Salmonella enterica serovar Typhi, and lipid A modifications (24). Further, a putative enoyl-coenzyme A hydratase appears to contribute to biofilm formation and antibiotic tolerance (25). Recent studies have highlighted the cytotoxicity of A. xylosoxidans for murine macrophages based on the potential cytotoxic role of the T3SS ubiquitin-activated phospholipase effector AxoU (26) and a giant repeats-in-toxin (RTX) adhesin (27). Comparative genomic studies of A. xylosoxidans isolated from acute and chronic infections indicate that the bacteria can adapt to chronic lung infection, as evidenced by increased antimicrobial resistance, decreased motility, alteration in biofilm formation, and reduced cytotoxicity (26, 28–31).

*Achromobacter* infections are difficult to treat due to resistance to aminoglycosides and β-lactams (32–36). This resistance can be conferred by intrinsic mechanisms, including chromosomally encoded efflux pumps and β-lactamases responsible for reducing the susceptibility of A. xylosoxidans to carbapenems, cephalosporins, and fluoroquinolones (35, 37). High-level intrinsic resistance is compounded by the acquisition of metallo-β-lactamases via mobile genetic elements, conferring resistance to carbapenems (38, 39), and can be further enhanced by chronic infection (36). Frequent coisolation with Pseudomonas aeruginosa in pwCF reduces the choices of antibiotics with efficacy against both genera (40, 41).

Due to the emergence of pan-antibiotic-resistant Gram-negative bacteria, polymyxins such as colistin (polymyxin E) and polymyxin B (PmB) are increasingly used as last-resort antibiotics. Colistin is commonly used as inhaled therapy in pwCF, and some CF centers treat *Achromobacter* infections with such nebulized therapies (42, 43). Colistin and PmB are cationic antimicrobial peptides (CAMPs) that disrupt the bacterial cell envelope by means of a cationic detergent-like mechanism. They have broad-spectrum activity against Gram-negative bacteria, and despite pharmacodynamic differences between colistin and PmB, the two peptides have comparable antimicrobial activities (44). Polymyxins enter bacterial cells by binding to negative charges in the lipid A moiety of the lipopolysaccharide (LPS). Self-promoted uptake of polymyxins has been proposed in which these molecules outcompete Mg^2+^ ions cross-linking LPS molecules at the outer membrane surface, bind to LPS, and cross the outer membrane (45, 46). Recent evidence suggests that polymyxins also target nascent LPS molecules in the inner membrane (47). Bacteria can become resistant to polymyxins and other CAMPs through modification of the lipid A. These modifications involve the addition of 4-amino-4-deoxy-l-arabinose or other positively charged moieties, which are added to the free phosphates of the lipid A, hence decreasing the negative charge of the lipid A or, alternatively, directly removing free phosphate groups from the lipid A (48). For example, in Bordetella pertussis, which is closely related to *Achromobacter* species, the lipid A can be modified by the addition of glucosamine (GlcN), resulting in bacterial resistance to CAMPs (49). The number, length, and hydroxylation of acyl chains to lipid A can also be modified, which potentially increases the lipid A packing in the outer membrane, further reducing the permeability of the outer membrane to CAMPs (48, 50, 51).

The phenomenon of heteroresistance, in which discrete subpopulations of bacteria have a higher level of antimicrobial resistance than the average resistance of the population (52), has been observed across several Gram-negative bacteria, especially in response to polymyxins (53, 54). Heteroresistance has not yet been reported in *Achromobacter*; however, the potential for bacterial isolates to harbor discrete subpopulations primed for resistance could explain therapeutic failures. Research on Enterobacter species has revealed that resistant bacterial subpopulations can also be selected without colistin treatment in response to the host immune system due to upregulation of genes responsible for lipid A modification (53).

CF isolates of A. xylosoxidans have been reported as generally susceptible to colistin at MIC values of the antibiotic below the suggested MIC clinical breakpoint of ≥4 μg/mL (21, 40, 43). In contrast, other studies have shown a bimodal colistin resistance in a subset of *Achromobacter* species from CF isolates and in biofilms and a steady increase in colistin resistance among *Achromobacter* isolates over time (34, 55, 56). The increased use of polymyxins as a last-resort treatment has resulted in the emergence of resistance due to a transmissible plasmid carrying the *mcr*-*1* gene encoding a phosphoethanolamine transferase (57, 58). However, colistin-resistant CF isolates of *Achromobacter* do not appear to harbor the *mcr*-*1* plasmid (56), suggesting that other mechanisms of polymyxin resistance operate in *Achromobacter* species.

In this study, an examination of a collection of *Achromobacter* clinical isolates from CF and non-CF sources showed that PmB resistance and heteroresistance are common. Characterization of the associated lipid A modifications, by chemical analyses and mass spectrometry of a subset of isolates, helped to correlate the effect of PmB challenge with lipid A modification and to establish the presence of PmB-resistant subpopulations within the isolates. These results indicate that lipid A modifications associated with PmB resistance are prevalent in *Achromobacter* and that subresistant populations displaying an increased addition of positively charged moieties and additional lipid A acyl chains can be selected for and isolated from PmB-sensitive *Achromobacter* clinical isolates.

## RESULTS

### Resistance to PmB is common in *Achromobacter* sp. clinical isolates.

**FIG 1:**
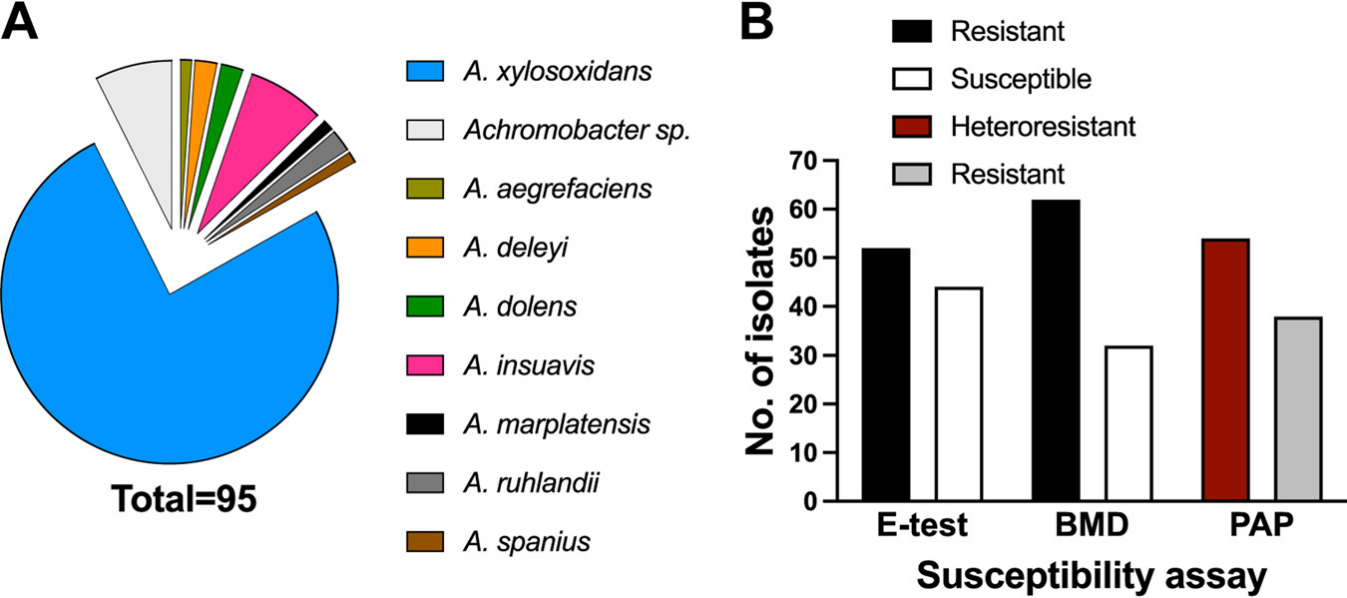
Analysis of the *Achromobacter* clinical isolate collection (*n* = 95) and A. xylosoxidans type strain ATCC 27061. (A) Incidence of *Achromobacter* species within the clinical isolate collection investigated in this study. Species identification was obtained via ribosomal, *gyrB*, or *nrdA* PCR. (B) Number of clinical isolates (including ATCC 27061) classed as susceptible, resistant, or heteroresistant to PmB. Isolates with an MIC of ≤4 μg/mL were deemed susceptible. MICs were established via Etest and broth microdilution (BMD) assay. Heteroresistance was established by population analysis profiling (PAP).

Clinical isolates of *Achromobacter* species (*n* = 95) isolated from blood and sputum were examined for resistance to PmB by Etest and broth microdilution (BMD) assays (Fig. 1A and Table 1). The A. xylosoxidans reference strain ATCC 27065 was included as a control. Using BMD, 62 out of the 94 *Achromobacter* isolates (66%) that grew in cation-adjusted Mueller-Hinton broth (CA-MHB) had MIC values of ≥4 μg/mL (Fig. 1B). When MIC values from the Etest were considered, 43 out of 95 isolates (45.3%) were found to be resistant (Fig. 1B). Also, the Etest plates of several isolates developed secondary halos between 1 and 6 days after plating, suggesting that these isolates consist of subpopulations of bacterial cells displaying heteroresistance. Comparison of the two tests based on breakpoint indicated disagreement in 16 isolates, with 13 of these showing PmB resistance by BMD but PmB sensitivity by Etest (Table 1). These differences can be explained by heteroresistance, which may be more apparent in broth culture since resistant subpopulations within a given isolate could continue growing in the presence of the antibiotic, resulting in turbidity at higher PmB concentrations relative to the no-antibiotic control. Heteroresistance was determined by population profile analysis as a ≥8-fold increase in the highest concentration of antibiotic with no inhibition and the lowest concentration with maximum inhibition (52, 59). Of the isolates for which resistance was observed by BMD but not by Etest, 54% were classed as heteroresistant. A similar analysis carried out on all the remaining isolates from our collection revealed that 50% had population profiles indicating heteroresistance (Table 1; see Data Set S1 in the supplemental material). Notably, while all the heteroresistant isolates were deemed resistant by BMD, 15% of the heteroresistant isolates were susceptible to PmB by Etest. Only 4 (31%) of the 13 blood isolates (Table 1) were resistant, suggesting that PmB resistance is more prevalent in the isolates obtained from chronic pulmonary infection. Together, our results demonstrate that resistance to PmB is common in clinical isolates of *Achromobacter* species, especially in those isolates obtained from chronic respiratory infection, and that some isolates display heteroresistance.

**TABLE 1:** Clinical isolate collection of *Achromobacter* spp.

Source^*a*^	Isolate no.	Species	Clinical source^*b*^	Etest MIC (μg/mL)	BMD^*c*^ MIC (μg/mL)	Resistance (S/R^*d*^)	PAP HR^*e*^
HSCNIMR	AC001	A. xylosoxidans	Unknown	0.125	1	S	N
HSCNIMR	AC002	A. xylosoxidans	Unknown	1	1	S	N
HSCNIMR	AC003	*A. spanius*	Unknown	>1,024	>256	R	NG
HSCNIMR	AC004	A. xylosoxidans	Respiratory isolate	>1,024	>256	R	NG
HSCNIMR	AC005	A. xylosoxidans	Respiratory isolate	1.5	8	R	Y
HSCNIMR	AC006	A. xylosoxidans	Respiratory isolate	0.125	1	S	N
HSCNIMR	AC007	A. xylosoxidans	Respiratory isolate	<0.064	1.7	S	N
HSCNIMR	AC008	A. xylosoxidans	Respiratory isolate	2	4	R	N
HSCNIMR	AC009	A. xylosoxidans	Respiratory isolate	6	4	R	N
HSCNIMR	AC010	A. xylosoxidans	Respiratory isolate	16	1	S	N
HSCNIMR	AC011	A. xylosoxidans	Respiratory isolate	4	64	R	Y
HSCNIMR	AC012	A. xylosoxidans	Respiratory isolate	4	5.7	R	Y
HSCNIMR	AC013	A. xylosoxidans	Respiratory isolate	16	>256	R	Y
HSCNIMR	AC014	A. xylosoxidans	Respiratory isolate	0.38	1	S	N
HSCNIMR	AC015	A. xylosoxidans	Respiratory isolate	0.38	1	S	N
HSCNIMR	AC016	A. xylosoxidans	Blood isolate	0.38	1	S	N
HSCNIMR	AC017	A. xylosoxidans	Blood isolate	0.5	1.7	S	N
HSCNIMR	AC018	A. xylosoxidans	Blood isolate	16	45.3	R	Y
HSCNIMR	AC019	A. xylosoxidans	Blood isolate	8	64	R	Y
HSCNIMR	AC020	A. xylosoxidans	Blood isolate	12	64	R	Y
HSCNIMR	AC021	A. xylosoxidans	Blood isolate	0.25	1.4	S	N
HSCNIMR	AC022	A. xylosoxidans	Blood isolate	0.19	1.4	S	N
HSCNIMR	AC023	A. xylosoxidans	Blood isolate	0.19	1.4	S	N
HSCNIMR	AC024	A. xylosoxidans	Blood isolate	0.064	1	S	N
HSCNIMR	AC025	A. xylosoxidans	Blood isolate	<0.064	1	S	N
HSCNIMR	AC026	A. xylosoxidans	Blood isolate	<0.064	1.2	S	N
HSCNIMR	AC027	A. xylosoxidans	Blood isolate	<0.064	11.3	R	N
HSCNIMR	AC028	A. xylosoxidans	Blood isolate	0.38	1	S	N
UKHSA	AC029	A. xylosoxidans	CF sputum	8	>256	R	Y
UKHSA	AC030	A. xylosoxidans	NCF, sputum	0.25	1	S	NG
UKHSA	AC031	A. xylosoxidans	CF	254	>256	R	Y
UKHSA	AC032	*A. insuavis* 2b	CF, sputum	0.25	1.4	S	N
UKHSA	AC033	A. xylosoxidans	CF, sputum	252	>256	R	Y
UKHSA	AC034	A. xylosoxidans	CF, sputum	8	45.3	R	Y
UKHSA	AC035	*Achromobacter* sp. cluster II	CF, sputum	1	8	R	N
UKHSA	AC036	A. xylosoxidans	CF	0.38	1.8	S	N
UKHSA	AC037	A. xylosoxidans	CF, sputum	8	>256	R	Y
UKHSA	AC038	A. xylosoxidans	CF, sputum	6	16	R	Y
UKHSA	AC039	*Achromobacter* sp. cluster II	CF, sputum	1	11.3	R	N
UKHSA	AC040	A. xylosoxidans	CF, sputum	0.5	181	R	Y
UKHSA	AC041	A. xylosoxidans	CF, sputum	1.5	22.6	R	Y
UKHSA	AC042	A. xylosoxidans	CF, BAL fluid	12	>256	R	Y
UKHSA	AC043	*Achromobacter* sp. cluster II	CF, sputum	32	>256	R	Y
UKHSA	AC044	A. xylosoxidans	CF, sputum	>1024	>256	R	Y
UKHSA	AC045	A. xylosoxidans	CF, sputum	1.5	64	R	Y
UKHSA	AC046	A. xylosoxidans	CF, sputum	24	>256	R	Y
UKHSA	AC047	*Achromobacter* sp. cluster II	CF, sputum	128	256	R	Y
UKHSA	AC048	A. xylosoxidans	CF, sputum	124	>256	R	Y
UKHSA	AC049	A. xylosoxidans	CF, cough swab	0.125	1	S	N
UKHSA	AC050	*A. dolens*	CF, sputum	12	32	R	Y
UKHSA	AC051	*A. ruhlandii*	CF, sputum	64	>256	R	Y
UKHSA	AC053	A. xylosoxidans	CF, sputum	6	22.6	R	Y
UKHSA	AC054	*A. insuavis* 2b	CF, sputum	1.5	1	S	N
UKHSA	AC055	A. xylosoxidans	CF, sputum	16	>256	R	Y
UKHSA	AC056	*A. insuavis* 2a	CF, cough swab	2	2	S	N
UKHSA	AC057	A. xylosoxidans	CF, sputum	384	>256	R	Y
UKHSA	AC058	A. xylosoxidans	CF, sputum	64	>256	R	Y
UKHSA	AC059	A. xylosoxidans	Unknown	4	64	R	Y
UKHSA	AC060	*A. dolens*	CF, sputum	4	32	R	Y
UKHSA	AC061	A. xylosoxidans	CF, sputum	12	>256	R	Y
UKHSA	AC062	A. xylosoxidans	CF, sputum	12	>256	R	Y
UKHSA	AC063	A. xylosoxidans	CF, sputum	8	>256	R	Y
UKHSA	AC064	A. xylosoxidans	CF, sputum	16	>256	R	Y
UKHSA	AC065	*A. aegrefaciens* 5b	CF, sputum	4	64	R	Y
UKHSA	AC066	A. xylosoxidans	CF, sputum	1	22.6	R	Y
UKHSA	AC067	A. xylosoxidans	CF, sputum	0.125	1	S	N
UKHSA	AC068	*A. deleyi*	CF, sputum	0.75	1.4	S	N
UKHSA	AC070	A. xylosoxidans	CF, sputum	32	128	R	Y
UKHSA	AC071	A. xylosoxidans	CF, sputum	1	128	R	Y
UKHSA	AC072	*A. ruhlandii*	CF, sputum	384	>256	R	Y
UKHSA	AC073	A. xylosoxidans	CF, sputum	16	>256	R	Y
UKHSA	AC074	*Achromobacter* sp. cluster II	CF, sputum	8	128	R	Y
UKHSA	AC075	*A. deleyi*	CF, sputum	3	22.6	R	Y
UKHSA	AC076	A. xylosoxidans	CF, sputum	1	>256	R	Y
UKHSA	AC077	*A. insuavis* 2a	CF, sputum	0.75	1.4	S	N
UKHSA	AC078	A. xylosoxidans	CF, sputum	16	>256	R	N
UKHSA	AC079	A. xylosoxidans	CF, sputum	16	>256	R	Y
UKHSA	AC080	A. xylosoxidans	CF, sputum	0.75	1	S	N
UKHSA	AC082	A. xylosoxidans	CF, sputum	1	1	S	N
UKHSA	AC083	*Achromobacter* sp. cluster I	CF, sputum	1	181	R	Y
UKHSA	AC084	A. xylosoxidans	CF, sputum	4	>256	R	Y
UKHSA	AC085	A. xylosoxidans	CF, sputum	3	8	R	N
UKHSA	AC086	A. xylosoxidans	CF, sputum	256	>256	R	Y
UKHSA	AC087	A. xylosoxidans	CF, sputum	16	>256	R	Y
UKHSA	AC088	A. xylosoxidans	CF, sputum	12	256	R	Y
UKHSA	AC089	A. xylosoxidans	CF, sputum	6	1	S	N
UKHSA	AC090	A. xylosoxidans	CF, sputum	4	128	R	Y
UKHSA	AC091	*A. insuavis* 2a	CF, sputum	8	1	S	N
UKHSA	AC092	*A. insuavis* 2b	COPD, sputum	2	1	S	N
UKHSA	AC093	*Achromobacter* sp.	CF, sputum	1.5	1	S	N
UKHSA	AC094	*A. marplatensis*	CF, cough swab	4	1	S	NG
UKHSA	AC095	A. xylosoxidans	CF, sputum	16	>256	R	Y
UKHSA	AC096	A. xylosoxidans	CF, sputum	16	>256	R	Y
UKHSA	AC097	*A. insuavis* 2a	Unknown	1.5	1.8	S	N
UKHSA	AC098	A. xylosoxidans	Unknown	12	>256	R	Y
ATCC 27061	ATCC	A. xylosoxidans	Otitis, ear discharge	3	16	R	Y

a HSCNIMR, Health and Social Care Northern Ireland Microbiology Repository; UKHSA, UK Health and Safety Agency; ATCC, American Type Culture Collection.

b BAL, bronchoalveolar lavage; COPD, chronic obstructive pulmonary disease; NCF, Non Cystic Fibrosis.

c BMD, broth microdilution assay.

d S/R, susceptible or resistant.

e PAP HR, heteroresistant by population analysis profile. Y, yes; N, no; NG, no growth.

### The lipid A of *Achromobacter* species is penta-acylated and can be modified by addition of glucosamine and palmitate.

**FIG 2:**
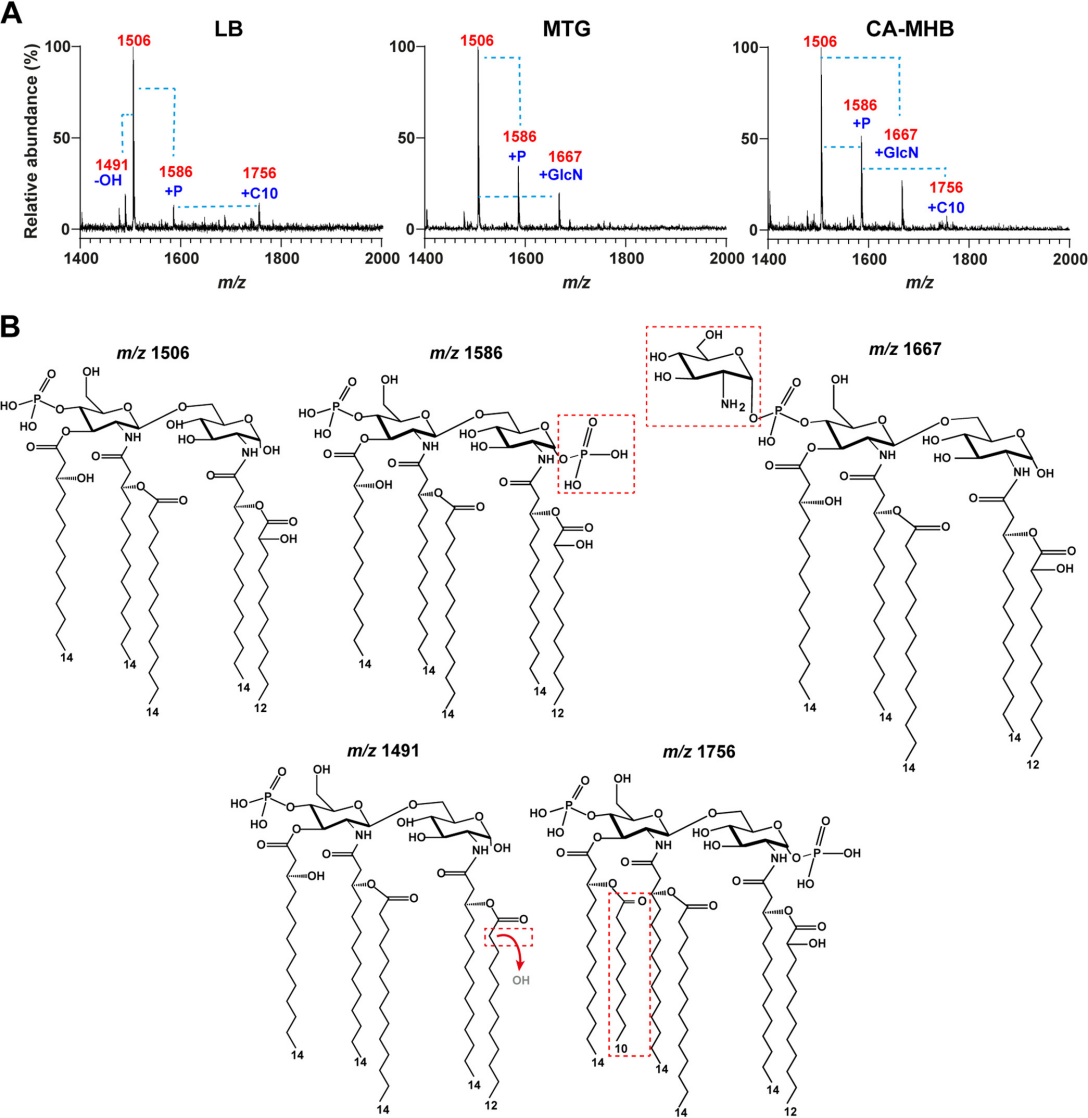
Basic lipid A profile of *Achromobacter* species. (A) MALDI-TOF spectra of AC075 lipid A from bacteria grown to log phase in lysogeny broth (LB), mineral-tryptone-glycerol (MTG), and cation-adjusted Mueller-Hinton broth (CA-MHB). Spectra were obtained via negative-ion reflectron MALDI-TOF mass spectrometry using a 2,5-dihydroxybenzoic acid matrix and lipid A isolated via citric acid lysis (62). Ion peaks representing the gain of a phosphate group (+P), a hydroxydecanoate acyl chain (+C10), and a glucosamine residue (+GlcN) and the loss of a hydroxyl group (−OH) are indicated by blue dotted lines. (B) Predicted lipid A structures. The additional phosphate group at position 4 (*m/z* 1,586), the addition of the GlcN residue (tentatively given in its α-anomeric configuration) at position 4′ (*m/z* 1,667), and the addition of a C-10 acyl chain (*m/z* 1,756) are indicated by dashed boxes. The loss of a 2-OH group on the C-12 acyl chain (*m/z* 1,491) is indicated by an arrow.

Modifications of the lipid A moiety of LPS are associated with resistance to polymyxins; these include the addition of positively charged residues to the phosphate groups and the alteration in length, number, and hydroxylation state of the acyl chains hypothesized to affect the integrity of the outer membrane (48–50, 60, 61). Because these modifications depend on environmental conditions, we first evaluated the effect of the growth medium (lysogeny broth [LB], CA-MHB, and mineral-tryptone-glycerol [MTG] medium) in the matrix-assisted laser desorption ionization–time of flight (MALDI-TOF) mass spectrometry (MALDI-MS) lipid A profiles of the isolate AC075 (Achromobacter deleyi), which were obtained by a rapid lysis method (62) and used to standardize the profiles. The most abundant ion peak in all three media during log-phase growth had a mass-to-charge (*m/z*) ratio of 1,506 (Fig. 2A), corresponding to a penta-acylated lipid A structure with one phosphate at the 4′ position and secondary acyl chains at 2 and 2′ positions (hydroxylaurate [C12:0(OH)], and myristate [C14:0(OH)], respectively), giving a 3 + 2 acylation pattern (Fig. 2B). A second peak at *m/z* 1,586 corresponds to the presence of another phosphate group (Fig. 2A and B). A third ion peak was also present in the lipid A from isolate AC075 grown in CA-MHB and MTG at *m/z* 1,667 (Fig. 2A), corresponding to a gain of 161 mass units of the *m/z* 1,506 ion peak; this was consistent with the addition of a HexN residue (61) (Fig. 2B). A relatively small ion peak at *m/z* 1,756 was interpreted as representing a small quantity of a hexa-acylated lipid A species with hydroxydecanoate [C10:0(OH)] (61). The results suggest that the growth medium influences the lipid A profiles, especially concerning the HexN modification. The lipid A profiles of A. xylosoxidans isolates AC011, AC036, AC061, AC064, and AC080 and Achromobacter insuavis AC054 were also examined from preparations using large-scale cultures in LB and treated to extract the lipid A as previously described (63) (Fig. S1). These preparations revealed additional peaks indicating variations in the hydroxylation of the acyl chains, but overall, they predicted that the *Achromobacter* species lipid A is mainly penta-acylated, with modifications resulting in the addition of HexN and in some cases an additional palmitoyl (C16:0) residue. Table S1 summarizes the predicted structural compositions of lipid A forms identified in this work.

To confirm the structural hypotheses deduced by MALDI-TOF MS, a detailed compositional analysis of these lipid A fractions directly extracted from cell pellets of A. xylosoxidans isolates AC011, AC036, AC061, AC064, and AC080 and *A. insuavis* AC054 was undertaken to establish both the fatty acid (Fig. S2 and Table S2) and sugar (Fig. S3) content. The analysis of acetylated methyl glycoside derivatives showed only the presence of GlcN in all the lipid A preparations (Fig. S3). This was further confirmed by the analysis of the alditol acetates of the unknown HexN properly separated from the lipid A upon dephosphorylation and inspection by means of gas chcromatography (GC)-MS. This analysis showed the presence of glucosaminitol acetate, thus definitively proving that the additional HexN unit decorating the disaccharide backbone of the *Achromobacter* lipid A molecular species investigated was a GlcN residue.

### LPS and lipid A modifications between early and late isolates.

Although most of the isolates were from individual patients, our collection included eight pairs of isolates from eight pwCF which were collected at intervals ranging from over 1 month up to more than 1 year. These pairs included isolates classified as *A. deleyi*, A. xylosoxidans, *A. insuavis*, and *Achromobacter* sp. cluster II (Table 2). Of these, one pair of isolates was susceptible to PmB, one pair showed a decrease in MIC when the early and late isolates were compared, and one pair showed an increased MIC from susceptible to resistant (Table 2). For the remaining pairs, the isolates were resistant (Table 2). To gain further insight that could explain the differences in PmB resistance, we investigated in more detail the MALDI-MS lipid A profiles of the isolate pairs that showed an increase in or maintenance of PmB resistance, which included AC036/AC088 (A. xylosoxidans), AC037/AC058 (A. xylosoxidans), AC046/AC048 (A. xylosoxidans), and AC068/AC075 (*A. deleyi*) (Table 2). Because no differences in lipid A structure were observed for AC075 in CA-MHB and MTG, we employed overnight cultures in MTG for all subsequent comparisons.

**TABLE 2:** PmB susceptibility of early and late isolate pairs from eight pwCF

Collection isolate no.	Species^*a*^	Time between isolations	E-test MIC (μg/mL)	BMD MIC (μg/mL)	Resistant^*b*^	Heteroresistant^*b*^
AC032	*A. insuavis 2b*	312 days	0.25	1.4	N	N
AC054	*A. insuavis 2b*		1.5	1	N	N
						
AC035	*Achromobacter* sp. cluster II	83 days	1	8	Y	N
AC039	*Achromobacter* sp. cluster II		1	11.3	Y	N
						
AC036	A. xylosoxidans	>1 yr	0.38	1.8	N	N
AC088	A. xylosoxidans		12	256	Y	Y
						
AC037	A. xylosoxidans	321 days	8	>256	Y	Y
AC058	A. xylosoxidans		64	>256	Y	Y
						
AC046	A. xylosoxidans	34 days	24	>256	Y	Y
AC048	A. xylosoxidans		124	>256	Y	Y
						
AC064	A. xylosoxidans	372 days	16	>256	Y	Y
AC079	A. xylosoxidans		16	>256	Y	Y
						
AC068	*A. deleyi*	193 days	0.75	1.4	N	N
AC075	*A. deleyi*		3	22.6	Y	Y
						
AC043	*Achromobacter* sp. cluster II	138 days	32	>256	Y	Y
AC074	*Achromobacter* sp. cluster II		8	128	Y	Y

a *Achromobacter* sp. cluster II was described by Coward et al. (12).

b Y, yes; N, no.

**FIG 3:**
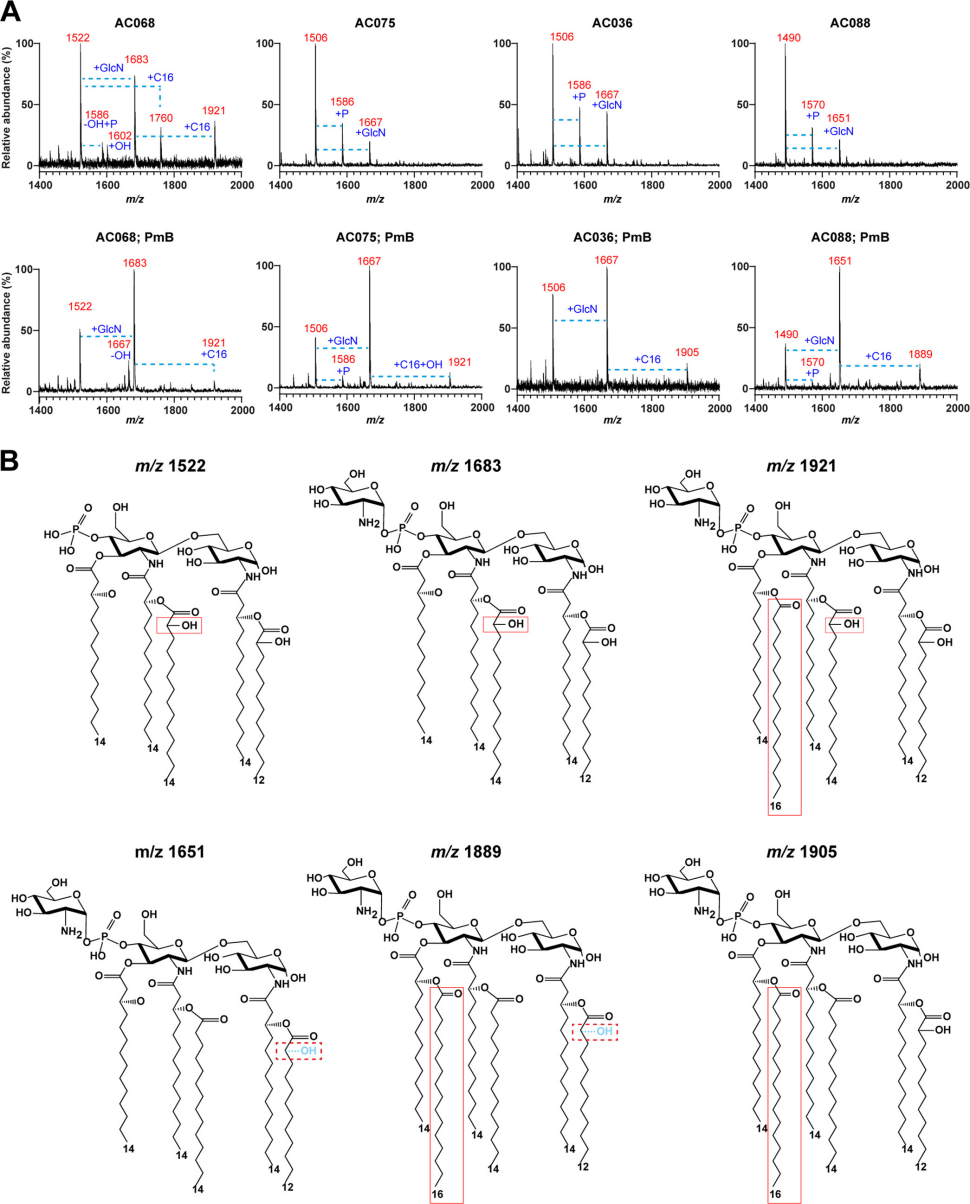
(A) Comparative MALDI-TOF spectra of early and late paired isolates AC068/AC075 and AC036/AC088 cultured to log phase in MTG without or with PmB at sub-MICs appropriate for each isolate. Spectra were obtained as described in the legend to Fig. 2. Ion peaks representing the gain of a phosphate group (+P), a palmitate acyl chain (+C16), and a glucosamine residue (+GlcN), as well as the gain (+OH) or loss (−OH) of hydroxyl groups, are indicated by blue dashed lines. (B) Predicted structures of the most abundant ion peaks. Solid boxes indicate hydroxylation or palmitoylation of the lipid A. Dashed boxes indicate the loss of hydroxylation in the secondary acyl chain at position C-3.

The lipid A of the early *A. deleyi* isolate AC068 exhibited a gain of 16 mass units at each of the main peaks seen in AC075 (Fig. 3A), giving peaks with *m/z* ratios of 1,522, 1,602, and 1,683. This indicates a gain of a hydroxyl group on the secondary myristate group at the C-2′ position (Fig. 3B and Table S1). A low-abundance peak with an *m/z* ratio of 1,586 was also present, which denotes the lack of the additional hydroxyl and the presence of a second phosphate, showing the potential for variation in lipid A structures even within a single strain. A hexa-acylated, GlcN-modified lipid A (*m/z* 1,921) (Fig. 3A) was present in AC068 and not in AC075. Variation in hydroxylation was also observed in the A. xylosoxidans AC036/AC088 pair (Fig. 3A). In AC036 lipid A, a monophosphorylated, penta-acylated structure (*m/z* 1,506) (Fig. 2B) with the gain of either a second phosphate (*m/z* 1,586) (Fig. 2B) or GlcN (*m/z* 1,667) (Fig. 2B) was predicted. In contrast, each peak in the lipid A spectrum of the AC088 late isolate corresponded to peaks like those in AC036 with a loss of 16 mass units (Fig. 3A). This suggests that the secondary myristate chains in the AC088 lipid A are not hydroxylated (Fig. 3B and Table 3; Table S1). No differences were observed in the lipid A of the AC037/AC058 and AC046/AC048 pairs of serial isolates, both of which displayed penta-acylated, monophosphorylated lipid A with hydroxylated secondary acyl chains (*m/z* 1,522), *m/z* 1,522 with GlcN modification (*m/z* 1,683), and *m/z* 1,683 plus a palmitoyl group (*m/z* 1,921), as with AC068 (Fig. 3B and Table 3; Table S1). Despite the fact that the small sample size precludes us from drawing unequivocal conclusions, our observations suggest that the lipid A of late isolates undergoes modifications involving various levels of hydroxylation of secondary acyl chains and GlcN addition, which could in turn confer high-level PmB resistance or decreased virulence, consistent with bacterial adaptation to chronic airway infection.

**TABLE 3:** Peaks indicating loss of a hydroxyl group between early and late isolates from two pwCF

Strains (early/late)	+OH *m/z* (early)	−OH *m/z* (late)	Position
AC068/AC075	1,522	1,506	Myristate on C-2′
	1,683	1,667	Myristate on C-2′
AC036/AC088	1,506	1,490	Laurate on C-4′
	1,586	1,570	Laurate on C-4′
	1,667	1,651	Laurate on C-4

The experiments described above were performed in the absence of PmB; therefore, the observed lipid A modifications could not be directly correlated with PmB exposure. We investigated the effect of subinhibitory PmB concentrations (ranging from 0.1 to 10 μg/mL, depending on the specific strain investigated) on the lipid A of the five pairs of isolates under investigation to identify the potential modifications induced in *Achromobacter* species following exposure to polymyxins. PmB concentrations were initially trialed using the MICs identified by BMD and gradually decreased if insufficient growth was observed overnight. Of the 10 isolates investigated, changes in lipid A structure following overnight culture in PmB were observed in A. xylosoxidans AC036 and AC088 and *A. deleyi* AC075 (Fig. 3A), while no changes were observed in the remaining strains when cultured in the presence or absence of PmB. MALDI-MS of AC036 lipid A showed penta-acylated structures, one of which contained the GlcN modification (*m/z* 1,667) (Fig. 2B and Fig. 3A). Upon growth in 0.1 μg/mL PmB, a gain of 238 mass units from *m/z* 1,667 to *m/z* 1,905 was observed (Fig. 3), indicating a gain of a palmitoyl group resulting in a hexa-acylated lipid A with a GlcN modification. The peak at *m/z* 1,586 corresponding to a bisphosphorylated and penta-acylated structure (Fig. 2B) was not observed in AC036 following growth in PmB (Fig. 3A). AC088 cultured in PmB showed a lipid A with a peak at *m/z* 1,889, an increase in 238 mass units from *m/z* 1,651, indicating the addition of a palmitate group (Fig. 3A), resulting in a hexa-acylated, GlcN-modified lipid A without hydroxylation of the secondary acyl chains (Fig. 3B). While MALDI-MS is not quantitative, the increased relative abundance of the peak at *m/z* 1,651 (Fig. 3A) and the decrease of the *m/z* 1,490 ion peak were consistent in both replicates, suggesting a greater proportion of GlcN-modified lipid A after treatment with PmB. Culturing AC075 in PmB revealed a lipid A with a gain of 254 mass units compared to *m/z* 1,667 (Fig. 3A), giving a peak at *m/z* 1,921 demonstrating the gain of a palmitate group (236 mass units) and a hydroxyl group (16 mass units) (Fig. 3B). The increased abundance of the GlcN-modified peak in AC088 was also seen in AC075 when treated with PmB (Fig. 3A), with the abundance of *m/z* 1,506 decreasing and that of *m/z* 1,667 increasing. The gain of a sixth acyl chain and a high relative abundance of GlcN-modified lipid A were found in isolates with high PmB resistance (≥32 μg/mL) without challenge with PmB (AC058, AC046, and AC048). No additional modification to these structures occurred following growth in PmB. Together, our results indicate that *Achromobacter* species have a heterogenous lipid A structure containing modifications that confer bacterial adaptation to high-level concentrations of PmB.

### *Achromobacter* species isolates can display heteroresistance to PmB.

**FIG 4:**
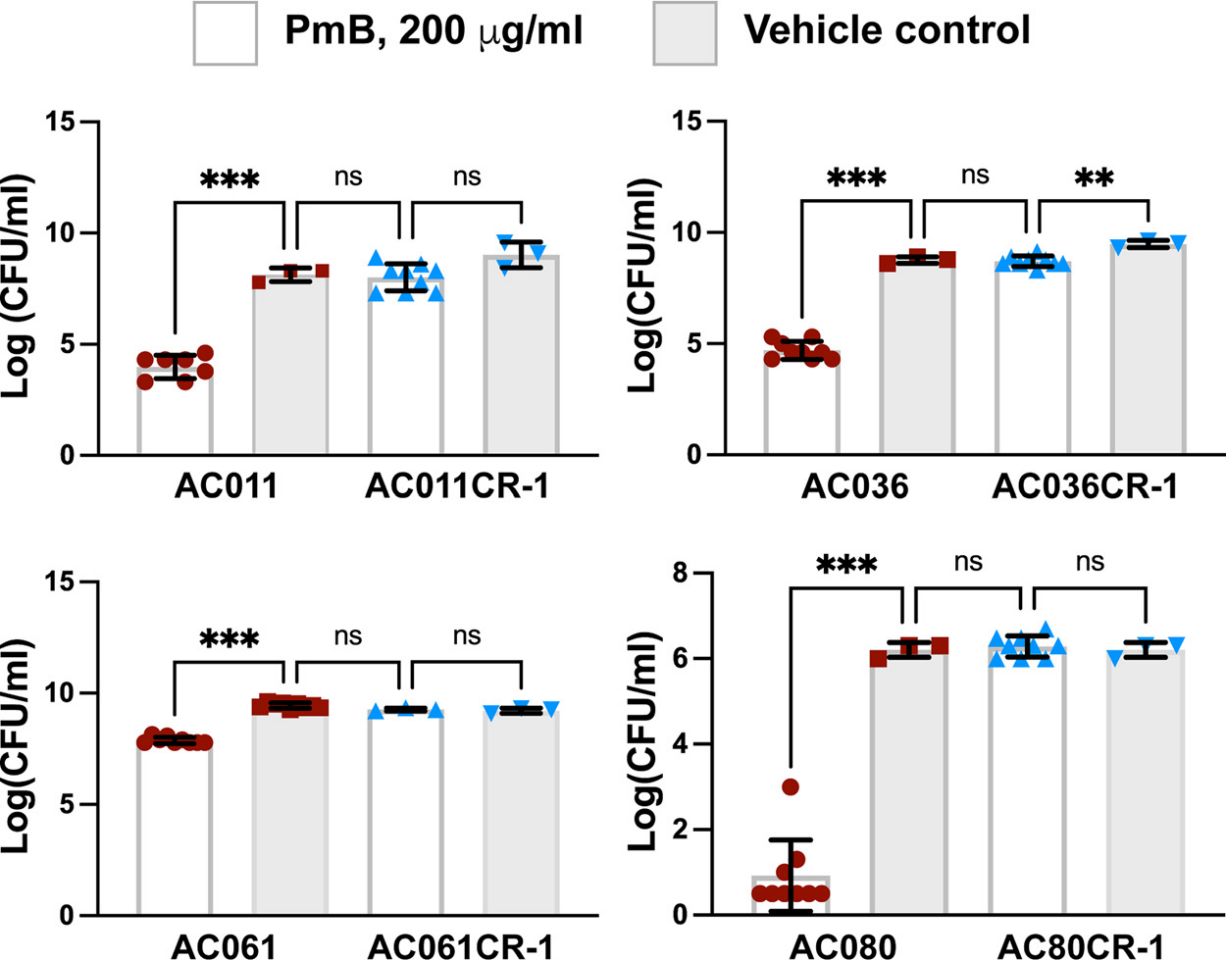
Comparison of the geometric mean of log_10_(CFU/mL) of the initial general population (*n* = 9) and heteroresistant subpopulation (*n* = 3) of the *Achromobacter* isolates when treated with the control (gray) and 200 μg/mL polymyxin B (black). CR-1 denotes the isolates recovered after the PmB challenge. **, *p* < 0.01; ***, *p* < 0.001; ns, nonsignificant.

The population profile analysis of all the isolates in our collection indicated that many display heteroresistance (Table 1). To better characterize this phenotype, the growth of every isolate in our collection was observed on a 200-μg/mL PmB LB plate. From this, the isolates that grew most successfully (AC011, AC036, AC061, and AC080) were selected for the PmB challenge assay. The results showed that when the clinical isolates were initially treated with PmB, there was a significant reduction in CFU/mL in comparison to the control group without antibiotic (mean log_10_ CFU increased to 4.9, 4.0, 1.6, and 5.7 from the challenge plate to the control plate for AC011, AC036, AC061, and AC080, respectively). The small differences between the challenge and control AC061 can be explained by the already high level of PmB resistance in this isolate (64 μg/mL BMD MIC). However, when the surviving colonies after challenge were challenged a second time, no significant differences were found with the control group of the initial isolate with no antibiotic (mean log CFU differences of 0.9, 0.3, −0.06, and 0 for AC011, AC036, AC061, and AC080, respectively). This indicates that they are resistant to PmB up to a minimum of 200 μg/mL following just two challenges of 2 h from polymyxin B (Fig. 4 and Table 4). Isolates recovered from the challenges were named with a CR-1 suffix (Fig. 4 and Table 4).

**TABLE 4:** Comparison of the geometric mean log_10_(CFU/mL) values of the initial clinical isolate (*n* = 9) and resistant subpopulation (*n* = 3) of *Achromobacter* isolates when treated with PmB (200 μg/mL) and a control

Isolate	Geometric mean log_10_(CFU/mL)			
	Susceptible general population		Resistant subpopulation	
	PmB treated	Control	PmB treated	Control
AC011	3.10	8.01	8.13	9.02
AC036	4.70	8.70	8.82	9.16
AC061	7.88	9.44	9.27	9.21
AC080	0.59	6.28	6.20	6.20

**FIG 5:**
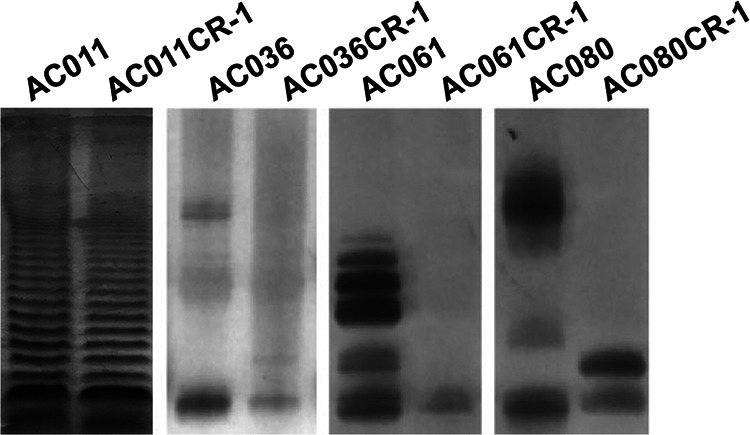
LPS profile comparison of the susceptible *Achromobacter* isolates and PmB-resistant subpopulations following a challenge assay (CR-1) using Tricine-SDS-PAGE and visualized by silver staining.

LPS profiling using silver staining revealed a noticeable reduction in higher-molecular-weight bands that represent the O-antigen polysaccharide in isolates AC036, AC061, and AC080 surviving PmB treatment compared to those of the untreated counterparts (Fig. 5). This suggests a switch in the production of O-antigen under PmB stress. In contrast, AC011 and its challenge resistant isolate AC011CR-1 showed no differences in O-antigen production. Loss of O-antigen polysaccharide, as described here, has been reported to confer PmB resistance to Escherichia coli strains even without modification of the phosphate of lipid A (64).

**FIG 6:**
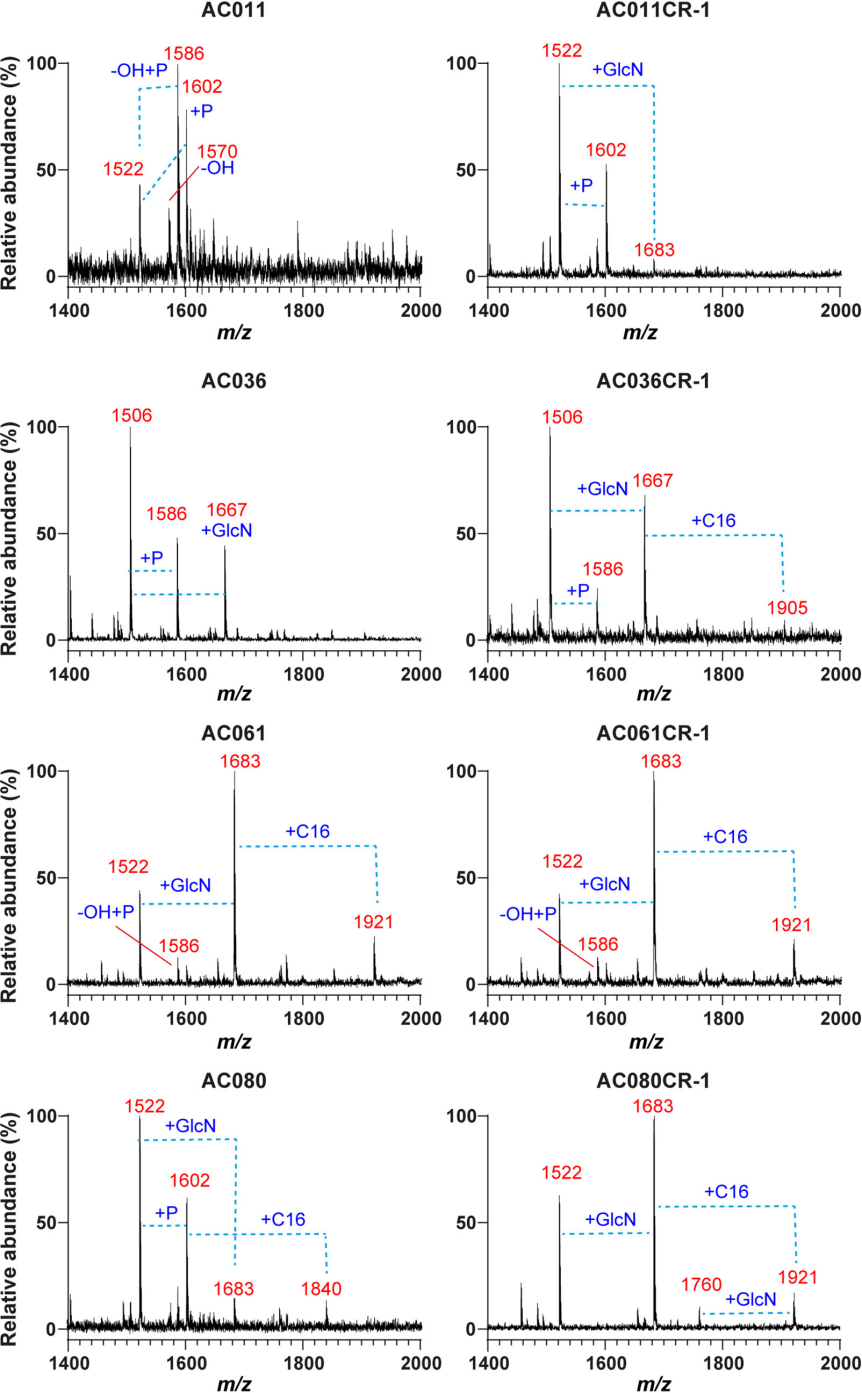
Lipid A mass spectrometry of A. xylosoxidans clinical isolates and their PmB challenge-resistant subpopulation. Spectra were obtained via negative-ion reflectron MALDI-TOF mass spectrometry using a DHB matrix and lipid A isolated via citric acid lysis following growth in MTG. Structural changes between peaks are indicated by dashed lines and annotated with the component responsible for the change in mass.

Analysis of the lipid A by MALDI-MS was carried out for the four clinical isolates and their challenge resistant isolates (Fig. 6). AC011 lipid A mass spectra predominantly displayed peaks consistent with a bisphosphorylated penta-acylated lipid A structure (*m/z* 1,586 and 1,602) with one or both secondary acyl chains hydroxylated, respectively. The lipid A of AC011CR-1 shows similar peaks at *m/z* 1,522 and *m/z* 1,602 with a drop in incidence of the single hydroxylated structures (*m/z* 1,586). AC011CR-1 also has the peak *m/z* 1,683, representative of a penta-acylated, monophosphorylated lipid A with a GlcN modification and both secondary acyl chains hydroxylated (Fig. 3B). The lipid A profile of AC036CR-1 showed the gain of a hexa-acylated, GlcN-modified lipid A structure (*m/z* 1,905) (Fig. 3B and Fig. 6). The lipid A of AC080 consisted of peaks at *m/z* 1,522, 1,602, and 1,683 and a peak at *m/z* 1,840 representative of a palmitoyl group giving a hexa-acylated, bisphosphorylated structure without a GlcN modification. However, AC080CR-1 showed an increase in the relative abundance of the GlcN-modified lipid A (*m/z* 1,683) (Fig. 3B). Additionally, the gain of a palmitoyl group giving a hexa-acylated structure with a GlcN modification (*m/z* 1,921) (Fig. 3B) was seen. No differences were found between the lipid A structures of AC061 and AC061CR-1, both displaying peaks at *m/z* 1,683 and 1,921, as in AC080CR-1, which could be related to the high PmB resistance (16 μg/mL by BMD) initially displayed by this isolate.

## DISCUSSION

This study has shown that PmB resistance and heteroresistance are common in clinical isolates of *Achromobacter* species, especially in respiratory isolates from pwCF, while blood isolates from non-CF patients were mostly PmB sensitive and did not show heteroresistance. Further, this study shows that *Achromobacter* isolates can modify their lipid A structures by the addition of GlcN, palmitoylation of the penta-acylated lipid A, dephosphorylation, and hydroxylation of secondary acyl chains. The gain of a sixth acyl chain, especially following challenge by PmB, could lead to increased resistance to polymyxins. The palmitoylation of lipid A has been shown to increase resistance to CAMPs, which are a component of the innate immune response (65), and palmitoylation of the lipid A in Salmonella and P. aeruginosa is suggested to increase the permeability barrier of the outer membrane against CAMPs (66–68). Similarly, disruption of the gene responsible for palmitoylation in Bordetella bronchiseptica resulted in the clearance of infection following the activation of the adaptive immune system, while the palmitoyl-containing wild-type strain persisted (69).

Although ion peaks consistent with bisphosphorylated lipid A structures (*m/z* 1,586 and 1,602) were present in many isolates, the peaks with the highest relative abundance in each strain were representative of monophosphorylated lipid A, especially in cases where lipid A was modified with GlcN (*m/z* 1,651, 1,667, 1,683, 1,889, 1,905, and 1,921). This adaptation has been observed in many Gram-negative pathogens, including Salmonella enterica and Helicobacter pylori, in which the loss of the C-1 phosphate group is associated with a higher resistance to PmB (70, 71). This confers resistance via the reduction in the overall negative charge of the lipid A molecule following the loss of a phosphate. Therefore, we propose that the GlcN modification combined with the loss of the C-1 phosphate contribute to PmB resistance in *Achromobacter* species.

Hydroxylation of the secondary acyl chains of lipid A molecules is common in many genera of Gram-negative bacteria and depends on the dioxygenase LpxO. Deletion of the *lpxO* gene has been associated with various phenotypes in Klebsiella pneumoniae, P. aeruginosa, Acinetobacter baumannii, and Burkholderia pseudomallei strains, including increased virulence and inflammatory responses, reduction of resistance to CAMPs, and increased macrophage-mediated killing across members of the genera (72–75). A recent study on Enterobacter cloacae complex isolates from neonatal sepsis demonstrated that isolates from asymptomatic patients carried a myristate group at the C-2 position in comparison to a hydroxymyristate at C-2 in sepsis-causing isolates (76). The authors hypothesize that the hydroxyl group at this position increases hydrogen bonding within the outer membrane, conferring resistance to CAMPs produced by the immune system, which would suggest that hydroxylation could be an adaptation to colonizing the lung environment. Both palmitoylation and hydroxylation are controlled by the PhoP/PhoQ regulatory system in other Gram-negative genera (77), but it is currently unknown if *Achromobacter* species harbor a PhoP/PhoQ regulator. Moreover, within the *Alcaligenaceae* family, the presence of C14:0 (2-OH) in the fatty acid profiling was reported only for species of *Bordetella* (78), Pigmentiphaga kullae (79), and representatives of *Kerstersia* (80). Therefore, the findings in this study extend these observations to *Achromobacter* species.

We hypothesize that in addition to CAMP resistance, the identified lipid A modifications may have implications in inflammation. Hexa-acylated lipid A is typically considered to be highly proinflammatory due to an enhanced interaction between lipid A, myeloid differentiation factor 2 (MD-2), and Toll-like receptor 4 (TLR4) provided by the presence of the sixth acyl chain, which in turn enhances dimerization of TLR4-MD2, leading to stronger stimulation of inflammatory responses (81). The penta-acylated lipid A structure in most of the *Achromobacter* isolates examined in this work suggests that their lipid A may be less proinflammatory than the canonical hexa-acylated structures. However, the GlcN modification has been associated with increased dimerization of TLR4 and the enhanced inflammatory response induced by the penta-acylated lipid A of B. pertussis (49). A similar observation has been made for Burkholderia cenocepacia, which produces a penta-acylated lipid A replaced by 4-amino-4-*deoxy*-l-arabinose (82). In this case, the 4-amino-4-*deoxy*-l-arabinose residue in the B. cenocepacia lipid A allows exposure of the fifth acyl chain on the surface of MD-2, enabling interactions with TLR4 and its dimerization. The change in lipid A between pairs of early and late isolates from the same patients was consistent with previous studies suggesting that *Achromobacter* species adapt to the surrounding environment and evolve during chronic infection (36). The chronicity of airway infections in pwCF could exert selective pressure on the pathogen, leading to higher mutation rates. Hypermutable clones causing the formation of genetically diverse subpopulations within the general bacterial population have been observed in Achromobacter ruhlandii (29, 30). In E. cloacae and K. pneumoniae, undetected heteroresistance to polymyxins leads to treatment failure in *in vivo* models of infection (53, 54). This can allow infections to reemerge and no longer respond to subsequent treatment after seemingly being eradicated after initial treatment, thus underscoring the potential clinical significance of this phenomenon.

In summary, this study demonstrates that resistance and heteroresistance to PmB in *Achromobacter* species, particularly in isolates from respiratory sources and chronic infection, are common phenomena. In addition, this study reveals that the *Achromobacter* lipid A has features resembling the lipid A from the closely related *Bordetella* genus and displays modifications that are consistent with increased bacterial resistance to polymyxins and potentially other CAMPs.

## MATERIALS AND METHODS

### Bacterial isolates.

The bacterial collection examined consisted of 95 *Achromobacter* clinical isolates representing different species of the genus, including A. xylosoxidans, A. spanius, *A. ruhlandii*, *A. insuavis*, A. marplatensis, A. dolens, A. deleyi, and *A. aegrifaciens*, which were obtained from sputum and blood and identified by *nrdA* (ribonucleoside-diphosphate reductase 1 subunit α) and *gyrB* (DNA gyrase subunit B) PCR gene amplification and DNA sequencing (12) (Table 1). These isolates were obtained from the Antimicrobial Resistance and Healthcare Associated Infections Reference Unit of the UK Health Security Agency and local clinical isolates from the Northern Ireland HSC Microbiology Culture Repository (MicroARK), housed at the Northern Ireland Public Health Laboratory, Belfast City Hospital, Belfast, Northern Ireland. Most of the sputum isolates originated from pwCF and were collected between January 2013 and June 2014 from 96 patients attending 27 UK hospitals, except for two samples taken from a non-CF patient and a patient with chronic obstructive pulmonary disease, while all blood isolates were from non-CF patients (Table 1).

### Culture conditions.

As the *Achromobacter* isolates displayed variable growth rates on lysogeny broth (LB) medium (Melford Laboratories, Ltd., Chelsworth, Ipswich, UK) depending on the strain, with some taking up to 4 days to grow, a semidefined mineral-tryptone-glycerol (MTG) medium was developed in our laboratory that provided consistent growth rates in most isolates and exploited the ability of most *Achromobacter* species to metabolize nitrates, since most species are denitrifying in their natural environment (83). MTG consisted of 15.1 mM (NH_4_)_2_SO_4_, 16.8 mM Na_2_HPO_4_ · 2H_2_O, 22 mM KH_2_PO_4_, 51.3 mM NaCl, 10 mM KNO_3_, 1% (vol/vol) glycerol as a carbon source, and 0.5% (wt/vol) tryptone, with a final pH of 6.7. A sterile 10× stock of these salts and glycerol was prepared and added after autoclaving to tryptone for broth and tryptone–1.5% agar for solid medium. All isolates were grown in MTG at 37°C. When indicated, isolates were also grown in cation-adjusted Mueller-Hinton broth (CA-MHB; Sigma-Aldrich) or high-salt LB.

### LPS extraction and characterization.

LPS was extracted by the hot aqueous-phenol extraction method and characterized using Tricine-SDS-PAGE as previously described, with some minor modifications (84). Overnight incubation in proteinase K was reduced to 3 h with identical results. LPS samples were diluted in loading buffer (188 mM Tris-HCl [pH 6.8], 6% SDS, 15% β-mercaptoethanol, 40% glycerol, and 0.01% bromophenol blue) at a loading dye-to-LPS ratio of 1:2 for storage at −20°C and further diluted at a ratio of 1:1 immediately before being run on a Tricine-SDS-PAGE gel using a 2-μL sample per well. Gels were stained with silver nitrate as previously described (84).

### MALDI-TOF mass spectrometry.

The characterization of *Achromobacter* lipid A was performed by matrix-assisted laser desorption ionization–time of flight (MALDI-TOF) mass spectrometry using a Bruker Autoflex MALDI-TOF spectrometer. Lipid A was isolated from either log-phase-grown bacteria in MTG or from overnight cultures in MTG containing the appropriate subinhibitory concentrations of PmB based on the MIC of each strain. Subinhibitory concentrations were determined by initially trialing 50% MIC and reducing the concentration further if insufficient growth was obtained. The bacteria were centrifuged at 3,220 × *g* for 10 min at room temperature before being resuspended in 1 mL of 0.1 M citric acid (62). The samples were centrifuged for 1 min at 14,500 × *g*, the supernatant was discarded, and the pellet was resuspended in 1 mL of 0.1 M citric acid. This washing step was repeated once before the performance of lipid A extraction and mass spectrometry as described by Kocsis et al. (62). For validation of lipid A mass spectrometry profiles, lipid A fractions from A. xylosoxidans isolates AC011, AC036, AC061, AC064, and AC080 and *A. insuavis* AC054 were isolated from cell pellets by the protocol described by El Hamidi et al. (85), with slight modifications. Briefly, an aliquot of each pellet (~1 mg) was washed with 500 μL of chloroform-methanol (1:2, vol/vol) and then with 500 μL of chloroform-methanol-water (3:2:0.25, vol/vol/vol), followed by several steps of centrifugation (1,200 × *g*, 30 min). Then, 500 μL of isobutyric acid acid–1 M ammonium hydroxide (5:3, vol/vol) was added to pellets and left at 100°C for 1 h. Upon centrifugation (1,200 × *g*, 30 min), the supernatants were collected, lyophilized, and washed several times with methanol, followed by a wash with chloroform-methanol-water (3:1.5:0.25, vol/vol/vol) (63). At this stage, the lipid A fractions were divided into four aliquots, three of which were chemically derivatized to analyze their lipid and sugar contents (see below), whereas the other one was prepared for MALDI-TOF MS analysis and mixed with a matrix solution of 2,4,6-trihydroxyacetophenone (THAP) in methanol–0.1% trifluoroacetic acid–acetonitrile (7:2:1, vol/vol/vol) at a concentration of 75 mg/mL. Lipid A-matrix solutions were spotted in triplicate on a MALDI plate. These MS spectra were recorded in reflectron mode, negative-ion polarity mode, on an ABSCIEX TOF/TOF 5800 Applied Biosystems (Foster City, CA, USA) mass spectrometer equipped with an neodymium-doped yttrium aluminum garnet laser (λ = 349 nm), with a 3-ns pulse width and a repetition rate of up to 1,000 Hz and equipped with delayed extraction technology. Each spectrum was a result of the accumulation of 2,000 laser shots.

### Chemical analyses of lipid A fractions.

To establish the nature of the sugars and lipids composing *Achromobacter* lipid A, an aliquot of each of the lipid A fractions isolated directly from cell pellets of A. xylosoxidans isolates AC011, AC036, AC061, AC064, and AC080 and *A. insuavis* AC054 underwent methanolysis (1.25 M hydrochloric acid in methanol, 80°C, 16 h), followed by acetylation (80°C, 20 min) and GC-MS analysis. In parallel, another aliquot of each lipid A was methanolized with 1.25 M hydrochloric acid in methanol (80°C, 2 h) and then extracted three times with hexane. The hexane layer, containing the fatty acids as methyl ester derivatives, was then injected into the GC-MS. Finally, to unequivocally define the nature of the additional hexosamine(s) decorating the *Achromobacter* lipid A, another aliquot of each lipid A preparation was treated with 50 μL of 48% aqueous hydrofluoric acid (HF) at 4°C for 16 h to remove the phosphate groups. The samples were placed in an ice bath, and the HF was evaporated until dry. Distilled water was added to the HF-treated samples, which were then lyophilized. A mixture of chloroform, methanol, and water (2:1:2, vol/vol/vol) was added to the samples and mixed. The upper parts were collected and lyophilized (86, 87). Each sample was then treated with a spatula tipful of NaBH_4_ (~5 mg), followed by acetylation with 25 μL of acetic anhydride and 25 μL of pyridine (85°C for 20 min) and GC-MS analysis. All the sugar and lipid derivatives were inspected by analyzing the MS fragmentation patterns and by comparing the gas chromatography profiles with those of opportunely prepared standards. All chemical analyses were performed on an Agilent Technologies gas chromatograph model 7820A equipped with a mass selective detector model 5977B and an HP-5 capillary column (Agilent, Milan, Italy; 30 m by 0.25 mm [inside diameter], flow rate of 1 mL/min, He as carrier gas). The temperature program used to analyze the sugar content was as follows: 140°C for 3 min and then 140°C increased to 240°C at 3°C/min. To analyze the fatty acid content, the following temperature program was used: 150°C for 5 min, 150°C increased to 280°C at 3°C/min, and 280°C for 5 min.

### Polymyxin B susceptibility testing and population analysis profiling.

There are no specific guidelines from the Clinical and Laboratory Standards Institute for breakpoints for colistin and PmB susceptibility among *Achromobacter* species. We adopted ≥4 μg/mL as a breakpoint concentration for PmB since this value was previously suggested to assess colistin resistance in *Achromobacter* isolates (43) and colistin and PmB have very similar antimicrobial activities (44). MIC values were determined by broth microdilution (BMD) performed in duplicate for PmB concentrations between 1 and 256 μg/mL. Inocula were prepared by diluting an overnight broth culture of each isolate in CA-MHB to reach a final inoculum of 5 × 10^5^ CFU/mL. One honeycomb 96-well plate (Oy Growth Curves Ab, Ltd., Finland) was used for each isolate. Each well received 180 μL of CA-MHB before the last well received 180 μL PmB at 569 μg/mL. The contents of the final well were then mixed via pipetting, the tip was changed, and 180 μL was transferred into the next well. This serial dilution was continued until 180 μL was discarded from the second-to-last well. Each well was then inoculated with 20 μL of the diluted bacteria. Plates were incubated at 37°C with continuous shaking in a Bioscreen C (Oy Growth Curves Ab, Ltd., Finland) for 24 h, with automatic readings of optical density at 600 nm (OD_600_) taken every 30 min. The MIC endpoint was read as the lowest concentration of antibiotic at which the percent OD_600_ relative to the no-antibiotic control was ≤10%, which corresponded to no visible growth. Resistance was also evaluated by Etest strips (AB bioMérieux, Solna, Sweden), which were applied to CA-MHB agar plates inoculated with test bacteria by spreading overnight cultures diluted to an optical density (OD_600_) of 0.5 corresponding to 5× 10^8^ CFU/mL onto the plates; the plates were incubated at 37°C for 24 h. Population analysis was performed as described previously (59), with some modifications. The percentage of bacterial growth relative to that of the no-PmB control was calculated from the OD_600_ readings at 24 h at all PmB concentrations. Isolates were defined as heteroresistant when the antibiotic concentration exhibiting the highest inhibitory effects was ≥8-fold higher than the highest noninhibitory concentration (52, 59).

### Polymyxin B challenge assays.

The presence of PmB-resistant subpopulations in cultured isolates was investigated by PmB challenge. Isolates were grown in MTG broth overnight, and an OD_600_ of 0.02 was adjusted in double-strength MTG broth. The PmB solution was made by dissolving 400 μg/mL polymyxin B sulfate in a solution of 0.2% bovine serum albumin (BSA) and 0.01% acetic acid. For the treatment group, the isolate inoculum was mixed with 800 μg/mL PmB solution; for the control group, only the BSA-acetic acid buffer was added. Bacteria were incubated for 2 h at 37°C with shaking and then serially diluted to 10^−6^ in phosphate-buffered saline (PBS). Aliquots (5 μL) of the serial dilutions were spread on MTG agar and incubated for 48 h at 37°C. Growth for each dilution was measured in CFU/mL (CFU/mL); the surviving colonies of the PmB treatment (CR-1) were isolated and retested for PmB resistance. The CFU/mL of each isolate was transformed by log_10_ before statistical analysis. The clinical isolates were tested in triplicate with three technical replicates each, and their CR-1 counterparts were tested in triplicate with one technical replicate each.

### Statistical analyses.

Statistical analyses were performed by Welch’s one-way analysis of variance (ANOVA) with an alpha of 0.05, and multiple comparisons were done by the Dunnett's test using Prism 9 (version 9.4.1; GraphPad Software, LLC).

### Data availability.

The original contributions presented in the study are included in the article and in the supplemental material. Further inquiries can be directed to the corresponding author.
