# Supplementary material for: Polymyxin Resistance and Heteroresistance Are Common in Clinical Isolates of Achromobacter Species and Correlate with Modifications of the Lipid A Moiety of Lipopolysaccharide
Source: Microbiol Spectr. 2022 Dec 15;11(1):e03729-22. doi: 10.1128/spectrum.03729-22 (PMC9927164; doi:10.1128/spectrum.03729-22)
Supplement: Supplemental file 1 — Supplemental material. Download spectrum.03729-22-s0001.pdf, PDF file, 6.0 MB [file spectrum.03729-22-s0001.pdf]

Supplementary Figures S1-S3, TABLES S1 and S2, and Supplementary Datafile 1

Polymyxin resistance and heteroresistance is common in clinical isolates of *Achromobacter* species and correlates with modifications of the lipid A moiety of the lipopolysaccharide

Lewis MacDonald, Sean Keenan, Flaviana Di Lorenzo, Nana E. Adade, Dervla T. D. Kenna, Beverley C. Millar, John E. Moore, José Ramos Vivas, Antonio Molinaro, and Miguel A. Valvano

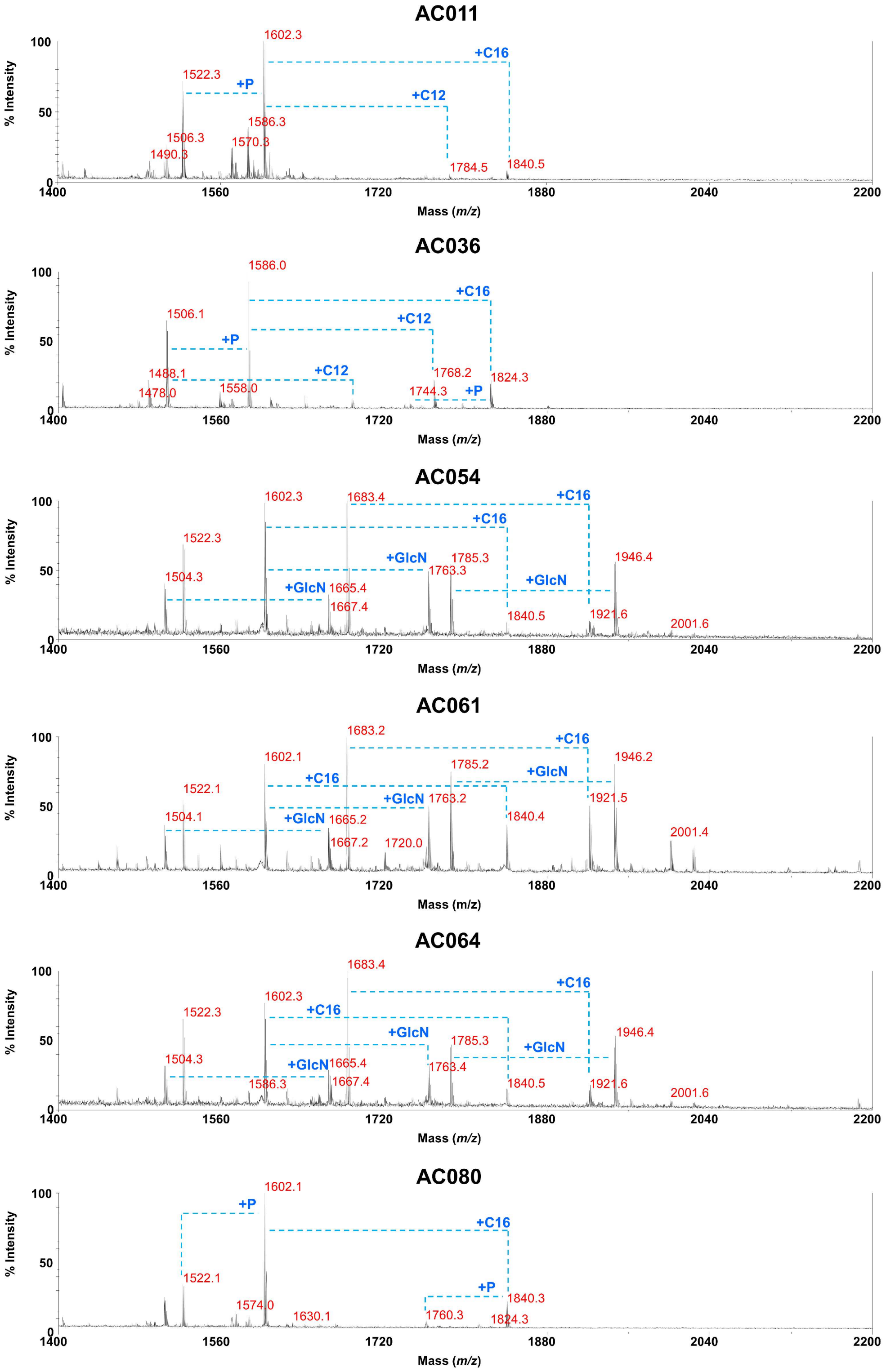

Figure S1. Lipid A profile of *A. xylosoxidans* isolates AC011, AC036, AC061, AC064 and AC080, and *A. insuavis* AC054. Spectra were obtained via negative ion reflectron MALDI-TOF mass spectrometry using THAP as the matrix. Lipid A fractions were isolated as previously described (63). Ion peaks representing the gain of phosphate groups (+P), hydroxylaurate (+C12) and palmitate (+C16) acyl chains, and glucosamine residues (+GlcN) are indicated by blue dotted lines.

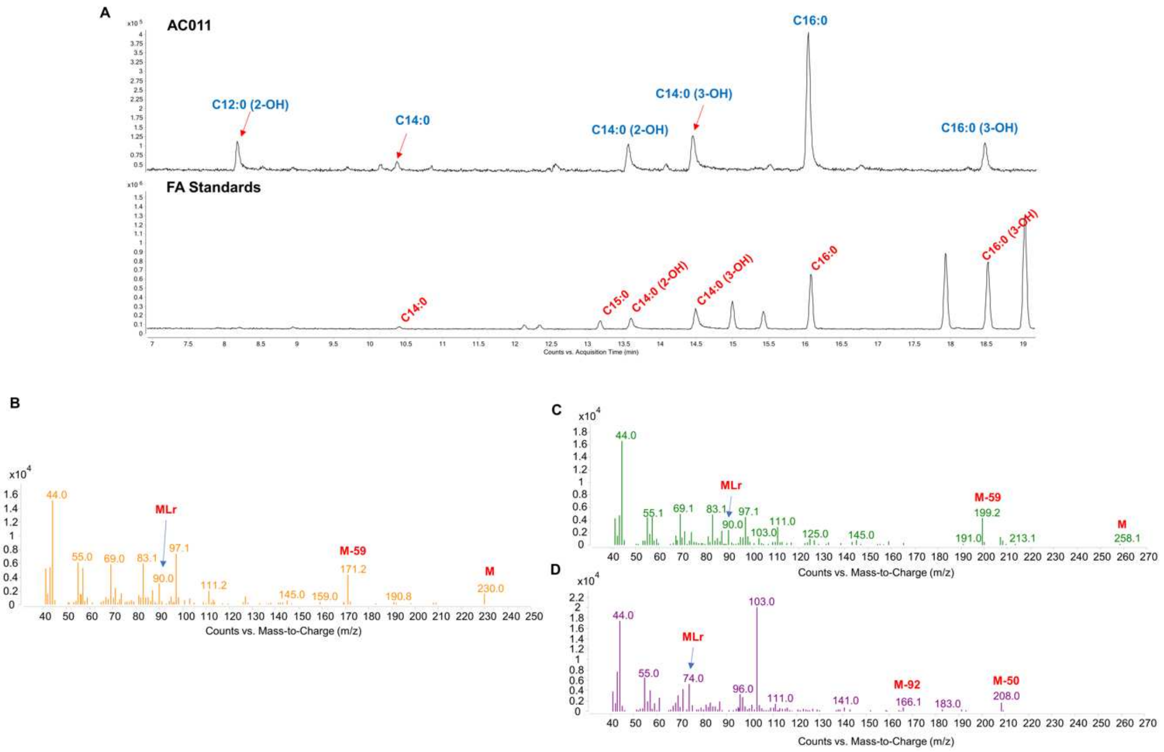

Figure S2. A. Zoom of the GC-MS chromatogram profile of the fatty acids as methyl ester derivatives of the lipid A from *A. xylosoxidans* isolate AC011 (top), chosen as a representative of the *Achromobacter* isolates analyzed in this study. The GC-MS chromatogram profile of opportunely prepared fatty acid methyl ester derivatives (bottom) is reported and compared with those obtained for *A. xylosoxidans* isolates AC011, AC036, AC061, AC064 and AC080, and *A. insuavis* AC054. **B**, **C**, and **D.** Electronic impact MS spectra that allowed the identification of C12:0(2-OH) (**B**), C14:0(2-OH) (**C**), and 14:0(3-OH) (**D**) in the lipid A preparations. "M" stands for molecular ion, while "MLr" indicates a fragment from McLafferty rearrangement. The detection of MLr ion at 90 u was diagnostic for the hydroxylation at C-2. On the contrary, the predominant ion at 103 u was indicative of the hydroxylation at C-3 as it is originated by the Cβ–Cγ fission and it comprises the first three carbon atoms of the acyl chain and the positive charge is stabilized from the β-hydroxyl function.

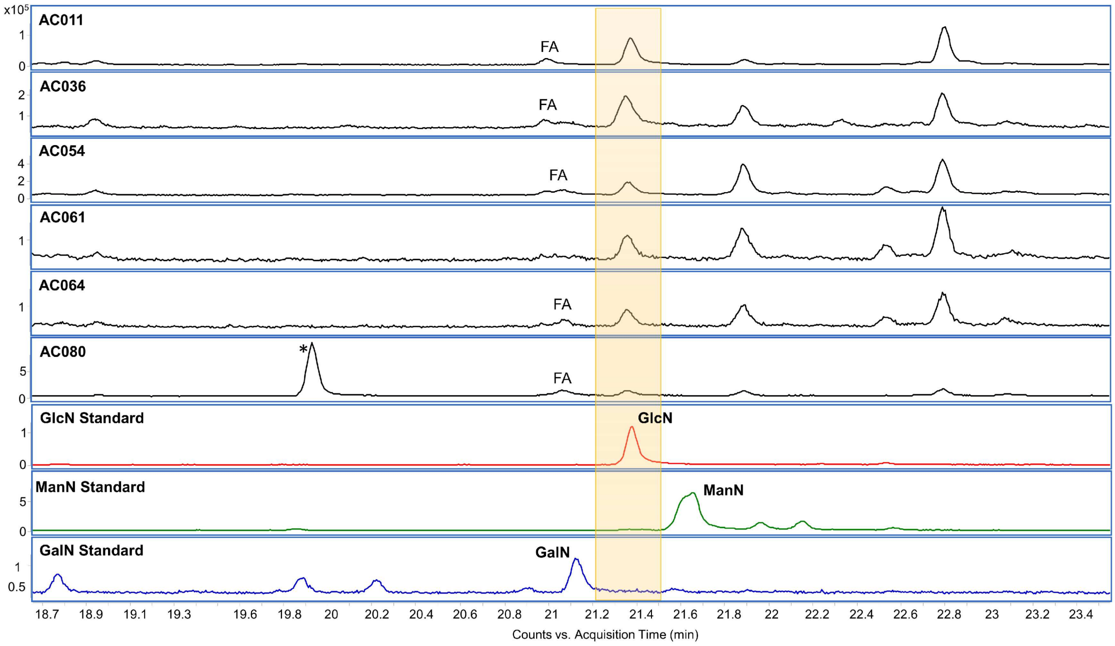

Figure S3. Zoom of the GC-MS chromatogram profiles of the acetylated methyl glycoside derivatives of the HexN isolated from the lipid A preparations of *A. xylosoxidans* isolates AC011, AC036, AC061, AC064 and AC080, and *A. insuavis* AC054. The GC-MS chromatogram profiles of the acetylated methyl glycoside of glucosamine, mannosamine, and galactosamine opportunely prepared from authentical standards were used as a reference and reported in the figure. The comparison of the retention times clearly demonstrated that the sole HexN present in the lipid A of *Achromobacter* isolates was a GlcN. The yellow box indicates the peaks relative to the acetylated methyl glycoside of hexosamine, whereas the asterisk indicates a contaminant. “FA” stands for fatty acid.

Table S1. Lipid A composition of *Achromobacter* species deduced from the observed *m/z* ion peaks

m/z	Predicted lipid A structure[a]
1490	Penta-acyl, 4x 14:0, 1x C12:0, 1P
1506	Penta-acyl, 3x C14:0(OH), 1x C14:0, 1x C12:0(OH), 1P
1522	Penta-acyl, 3x C14:0(OH), 1x C12:0(OH), 1x C14:0, 1P
1559	Penta-acyl, 3x C14:0(OH), 1x 10:0(OH), 1x C14:0, 2P
1570	Penta-acyl, 3x C14:0(OH), 1x C14:0, 1x C12:0, 2P
1586	Penta-acyl, 3x C14:0(OH), 1x C14:0, 1x C12:0(OH), 2P
1602	Penta-acyl, 4x C14:0(OH), 1x C12:0(OH), 2P
1642	Penta-acyl, 4x C14:0(OH), 1x C16:0, 2P
1651	Penta-acyl, 3x C14:0(OH), 1x C14:0, 1x C12:0, 1P, GlcN
**1667***	Penta-acyl, 3x C14:0(OH), 1x C14:0, 1x C12:0(OH), 1P, GlcN
1683	Penta-acyl, 4x C14:0(OH), 1x C12:0(OH), 1P, GlcN
1688	Hexa-acyl, 3x C14:0(OH), 1x C12:0(OH), 1x C14:0, 1x C12:0, 1P
1744	Hexa-acyl, 3xC14:0(OH), 1x C14:0, 1x C12:0(OH), 1x C16:0, 1P
1760	Penta-acyl, 4xC14:0(OH), 1x C12:0(OH), 1x C16:0, 1P
1768	Hexa-acyl, 4x C14:0(OH), 2x C12:0, 2P **or** 3x C14:0(OH), 1x C12:0(OH), 1x C14:0,
1x	C12:0, 2P
1785	Hexa-acyl, 4x C14:0(OH), 1x C10:0(OH), 1x 14:0, 2P
**1824***	Hexa-acyl, 3x C14:0(OH), 1x C14:0, 1x C12:0(OH), 1x C16:0, 2P
1840	Penta-acyl, 4xC14:0(OH), 1x C12:0(OH), 1x C16:0, 2P
1889	Hexa-acyl, 3xC14:0(OH), 1x C16:0, 1x C12:0, 1x C12:0(OH), 1P, GlcN
1905	Hexa-acyl, 3xC14:0(OH), 1x C16:0, 1x C14:0, 1x C12:0(OH), 1P, GlcN
1921	Hexa-acyl, 4xC14:0(OH), 1x C16:0, 1x C12:0(OH), 1P, GlcN
1946	Hexa-acyl, 4x C14:0(OH), 1x C10:0(OH), 1x 14:0, 2P, GlcN
2001	Hexa-acyl, 4x C14:0(OH), 1x C12:0(OH), 1x 16:0, 2P, GlcN

* The lipid A structures are predicted based on the observed *m/z* ion peaks. The canonical GlcN disaccharide is included in the calculations. GlcN, when indicated is the modification added to the phosphate residue. Peaks in bold with an asterisk may have alternative composition, which will require validation by more detailed structural analysis.

Table S2. Fatty acid content of lipid A isolated from *A. xylosoxidans* isolates AC011, AC036, AC061, AC064 and AC080, and *A. insuavis* AC054. A heterogenous composition was observed, which was however qualitatively equal for all the isolates. The absolute configuration of 2 and 3-hydroxy fatty acids remains to be defined.

Fatty Acid Component
12:0
14:0
16:0
10:0(3-OH)
12:0(2-OH)
12:0(3-OH)
14:0(2-OH)
14:0(3-OH)
16:0(3-OH)

Supplemental Dataset 1

MIC determination and population analysis profiles of all *Achromobacter* strains investigated in this study

MIC values were determined by broth microdilution (BMD) performed in duplicate for PmB concentrations between 1-256 µg/mL. Inocula were prepared by diluting an overnight broth culture of each isolate in CA-MHB to reach a final inoculum of 5 x 10^5 CFU/ml. One honeycomb 96-well plate (Oy Growth Curves Ab Ltd, Finland) was used for each isolate. Each well received 180 µL of CA-MHB before the last well received 180 µL PmB at 569 µg/mL. The final well was then mixed via pipetting, the tip changed, and 180 µL transferred into the next well. This serial dilution was continued until 180 µL was discarded from the second to last well. Each well was then inoculated with 20 µL of the diluted bacteria. Plates were incubated at 37°C with continuous shaking in a Bioscreen C (Oy Growth Curves Ab Ltd, Finland) for 24 h taking automatic OD_{600} readings every 30 min. The MIC endpoint was read as the lowest concentration of antibiotic at which the percent OD_{600} relative to the no antibiotic control was ≤10%, which corresponded to no visible growth.

Population analysis was performed by calculating the percent of bacterial growth relative to the no PmB control from the OD600 readings at 24 h in all PmB concentrations. Isolates were defined as heteroresistant when the antibiotic concentration exhibiting the highest inhibitory effects was ≥ 8-fold higher than the highest non-inhibitory concentration.

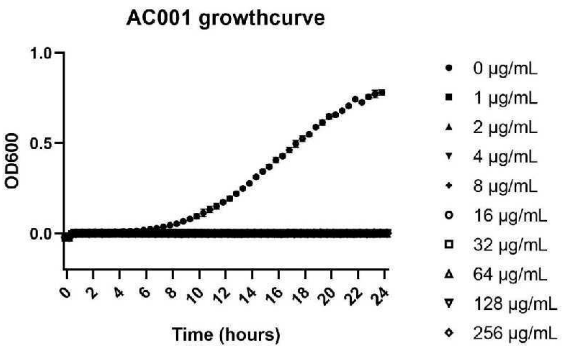
AC001 growthcurve
1.0
0.5
0.0
OD600
0
2
4
6
8
10
12
14
16
18
20
22
24
Time (hours)
0 µg/mL
1 µg/mL
2 µg/mL
4 µg/mL
8 µg/mL
16 µg/mL
32 µg/mL
64 µg/mL
128 µg/mL
256 µg/mL

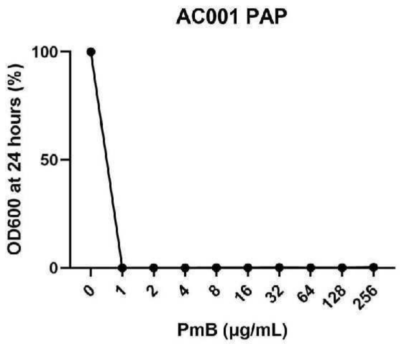
AC001 PAP
100
50
0
OD600 at 24 hours (%)
0
1
2
4
8
16
32
64
128
256
PmB (µg/mL)

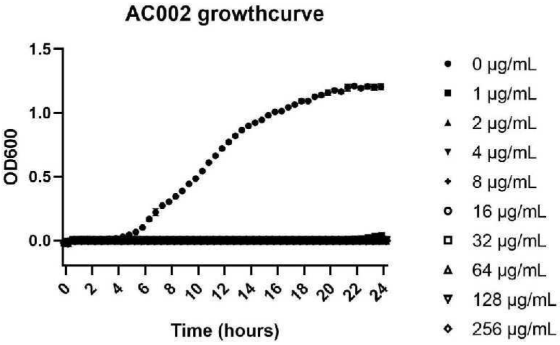
AC002 growthcurve
1.5
1.0
0.5
0.0
OD600
0
2
4
6
8
10
12
14
16
18
20
22
24
Time (hours)
0 µg/mL
1 µg/mL
2 µg/mL
4 µg/mL
8 µg/mL
16 µg/mL
32 µg/mL
64 µg/mL
128 µg/mL
256 µg/mL

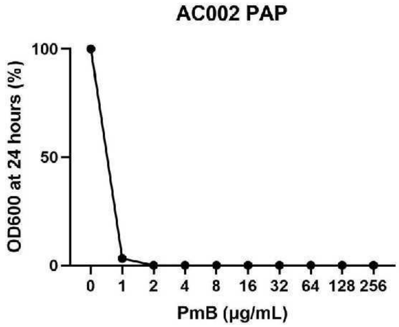
AC002 PAP
100
50
0
OD600 at 24 hours (%)
0
1
2
4
8
16
32
64
128
256
PmB (µg/mL)

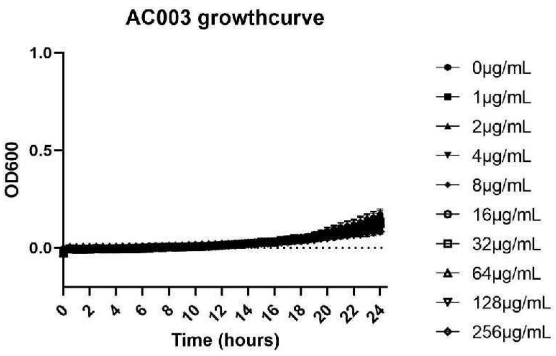
AC003 growthcurve
1.0
0.5
0.0
OD600
0
2
4
6
8
10
12
14
16
18
20
22
24
Time (hours)
0μg/mL
1μg/mL
2μg/mL
4μg/mL
8μg/mL
16μg/mL
32μg/mL
64μg/mL
128μg/mL
256μg/mL

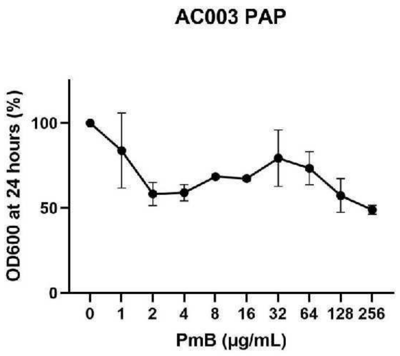
AC003 PAP
100
50
0
OD600 at 24 hours (%)
0
1
2
4
8
16
32
64
128
256
PmB (μg/mL)

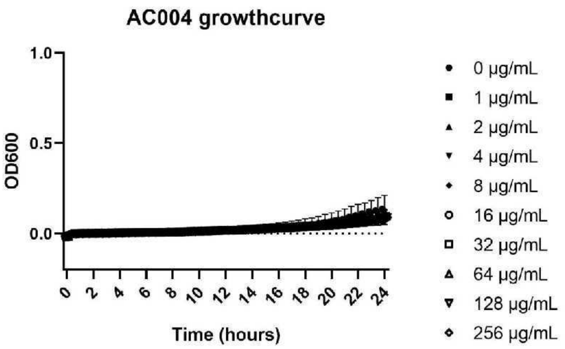
AC004 growthcurve
1.0
0.5
0.0
OD600
0
2
4
6
8
10
12
14
16
18
20
22
24
Time (hours)
0 μg/mL
1 μg/mL
2 μg/mL
4 μg/mL
8 μg/mL
16 μg/mL
32 μg/mL
64 μg/mL
128 μg/mL
256 μg/mL

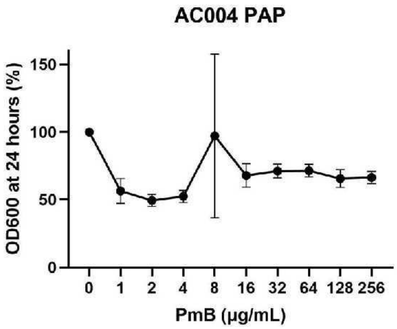
AC004 PAP
150
100
50
0
OD600 at 24 hours (%)
0
1
2
4
8
16
32
64
128
256
PmB (μg/mL)

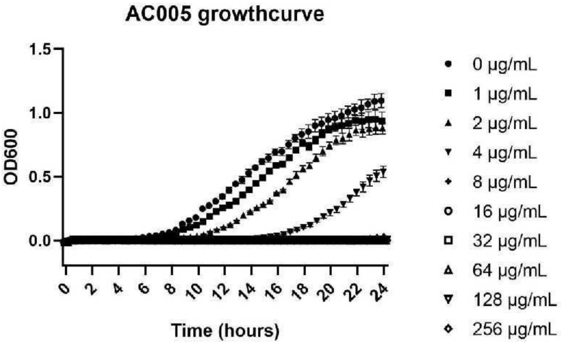
AC005 growthcurve
1.5
1.0
0.5
0.0
OD600
0
2
4
6
8
10
12
14
16
18
20
22
24
Time (hours)
0 µg/mL
1 µg/mL
2 µg/mL
4 µg/mL
8 µg/mL
16 µg/mL
32 µg/mL
64 µg/mL
128 µg/mL
256 µg/mL

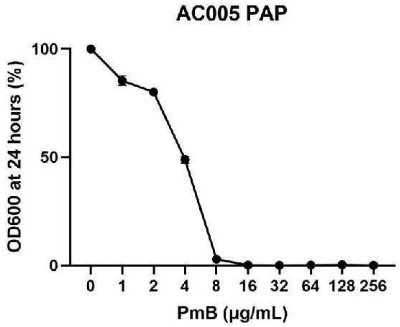
AC005 PAP
100
50
0
OD600 at 24 hours (%)
0
1
2
4
8
16
32
64
128
256
PmB (µg/mL)

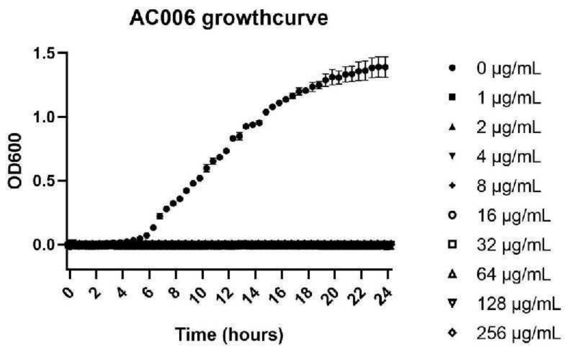
AC006 growthcurve
1.5
1.0
0.5
0.0
OD600
0
2
4
6
8
10
12
14
16
18
20
22
24
Time (hours)
0 µg/mL
1 µg/mL
2 µg/mL
4 µg/mL
8 µg/mL
16 µg/mL
32 µg/mL
64 µg/mL
128 µg/mL
256 µg/mL

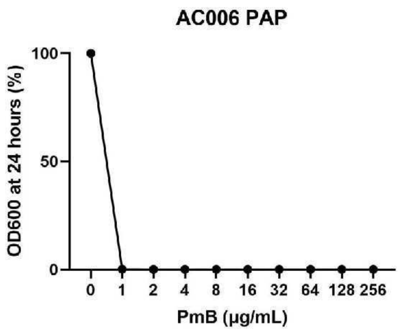
AC006 PAP
100
50
0
OD600 at 24 hours (%)
0
1
2
4
8
16
32
64
128
256
PmB (µg/mL)

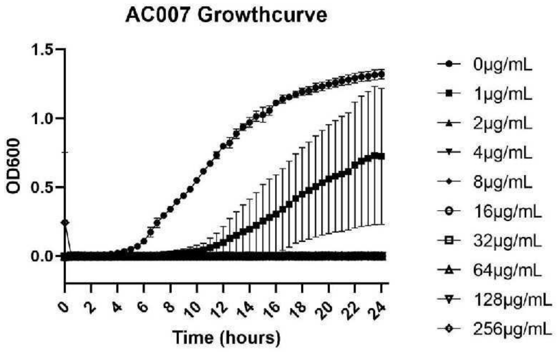
AC007 Growthcurve
1.5
1.0
0.5
0.0
OD600
0
2
4
6
8
10
12
14
16
18
20
22
24
Time (hours)
0µg/mL
1µg/mL
2µg/mL
4µg/mL
8µg/mL
16µg/mL
32µg/mL
64µg/mL
128µg/mL
256µg/mL

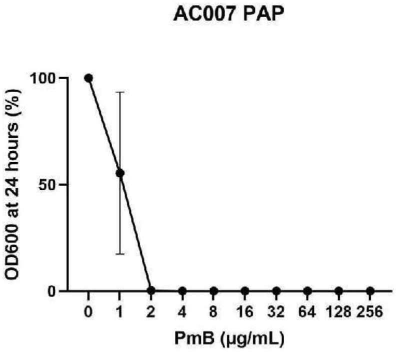
AC007 PAP
100
50
0
OD600 at 24 hours (%)
0
1
2
4
8
16
32
64
128
256
PmB (µg/mL)

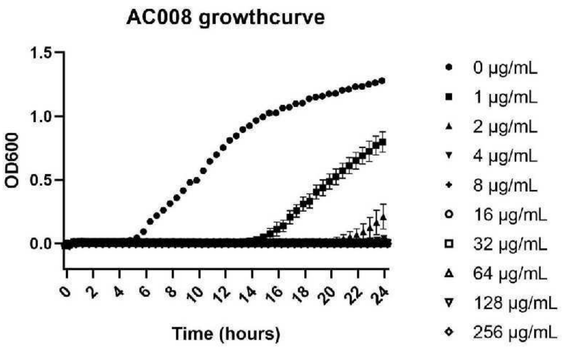
AC008 growthcurve
1.5
1.0
0.5
0.0
OD600
0
2
4
6
8
10
12
14
16
18
20
22
24
Time (hours)
0 µg/mL
1 µg/mL
2 µg/mL
4 µg/mL
8 µg/mL
16 µg/mL
32 µg/mL
64 µg/mL
128 µg/mL
256 µg/mL

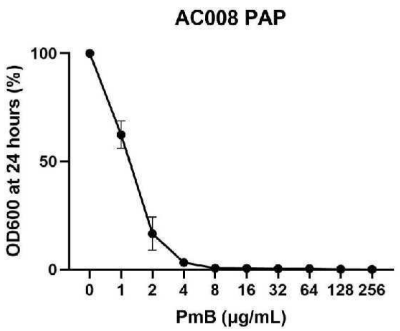
AC008 PAP
100
50
0
OD600 at 24 hours (%)
0
1
2
4
8
16
32
64
128
256
PmB (µg/mL)

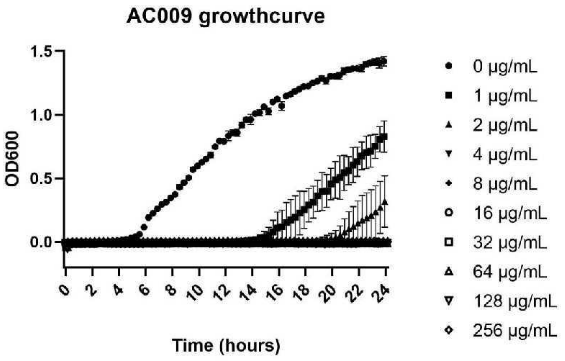
AC009 growthcurve
OD600
Time (hours)
0 µg/mL
1 µg/mL
2 µg/mL
4 µg/mL
8 µg/mL
16 µg/mL
32 µg/mL
64 µg/mL
128 µg/mL
256 µg/mL

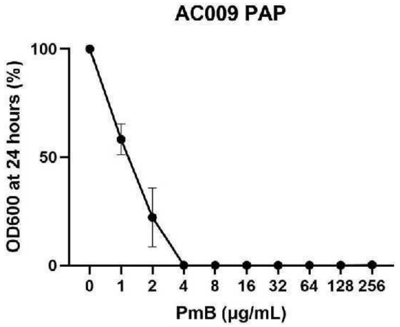
AC009 PAP
OD600 at 24 hours (%)
PmB (µg/mL)

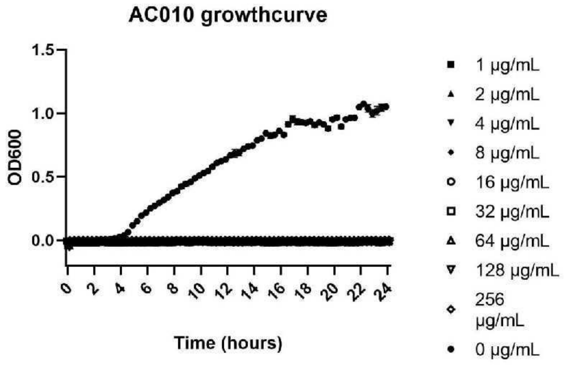
AC010 growthcurve
OD600
1.5
1.0
0.5
0.0
0
2
4
6
8
10
12
14
16
18
20
22
24
Time (hours)
1 µg/mL
2 µg/mL
4 µg/mL
8 µg/mL
16 µg/mL
32 µg/mL
64 µg/mL
128 µg/mL
256 µg/mL
0 µg/mL

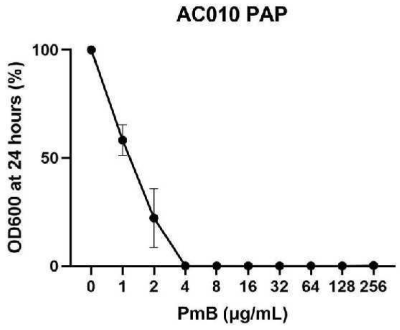
AC010 PAP
OD600 at 24 hours (%)
100
50
0
0
1
2
4
8
16
32
64
128
256
PmB (µg/mL)

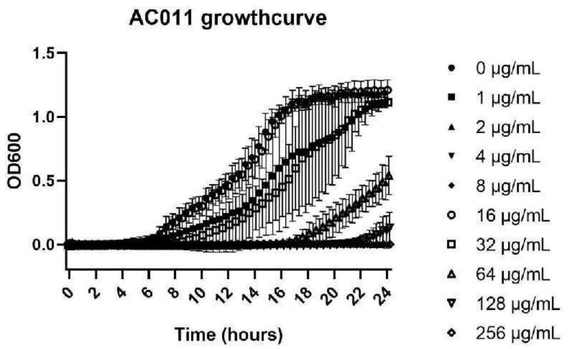
AC011 growthcurve
1.5
1.0
0.5
0.0
OD600
0
2
4
6
8
10
12
14
16
18
20
22
24
Time (hours)
0 µg/mL
1 µg/mL
2 µg/mL
4 µg/mL
8 µg/mL
16 µg/mL
32 µg/mL
64 µg/mL
128 µg/mL
256 µg/mL

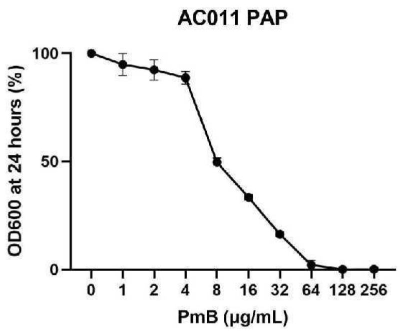
AC011 PAP
100
50
0
OD600 at 24 hours (%)
0
1
2
4
8
16
32
64
128
256
PmB (µg/mL)

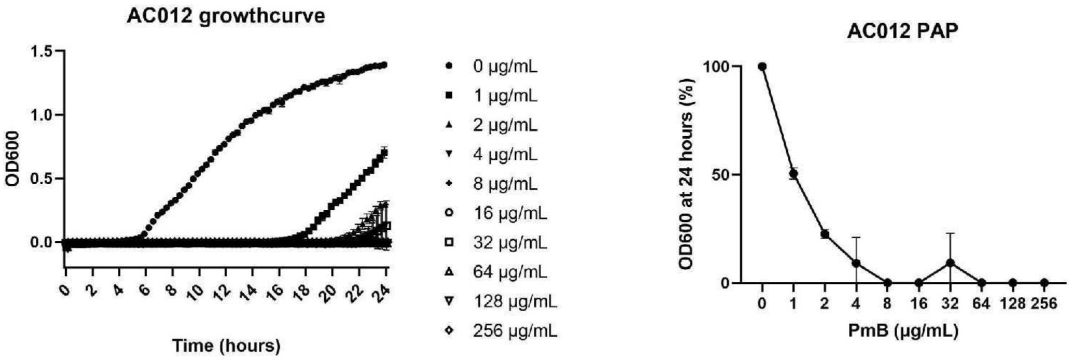
AC012 growthcurve
OD600
1.5
1.0
0.5
0.0
0 2 4 6 8 10 12 14 16 18 20 22 24
Time (hours)
0 µg/mL
1 µg/mL
2 µg/mL
4 µg/mL
8 µg/mL
16 µg/mL
32 µg/mL
64 µg/mL
128 µg/mL
256 µg/mL
AC012 PAP
OD600 at 24 hours (%)
100
50
0
0 1 2 4 8 16 32 64 128 256
PmB (µg/mL)

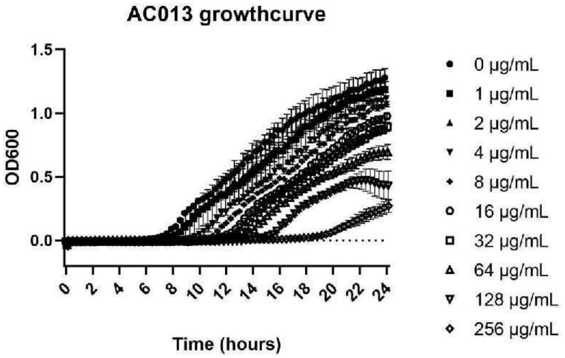
AC013 growthcurve
1.5
1.0
0.5
0.0
OD600
0
2
4
6
8
10
12
14
16
18
20
22
24
Time (hours)
0 µg/mL
1 µg/mL
2 µg/mL
4 µg/mL
8 µg/mL
16 µg/mL
32 µg/mL
64 µg/mL
128 µg/mL
256 µg/mL

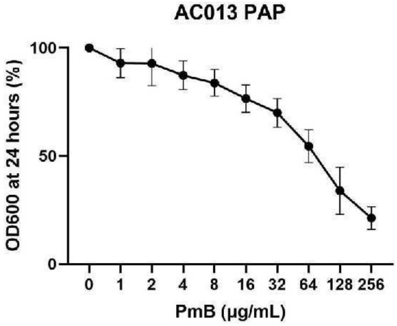
AC013 PAP
100
50
0
OD600 at 24 hours (%)
0
1
2
4
8
16
32
64
128
256
PmB (µg/mL)

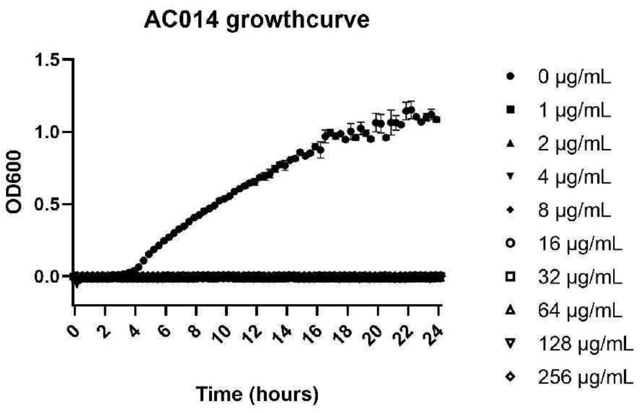
AC014 growthcurve
1.5
1.0
0.5
0.0
OD600
0
2
4
6
8
10
12
14
16
18
20
22
24
Time (hours)
0 µg/mL
1 µg/mL
2 µg/mL
4 µg/mL
8 µg/mL
16 µg/mL
32 µg/mL
64 µg/mL
128 µg/mL
256 µg/mL

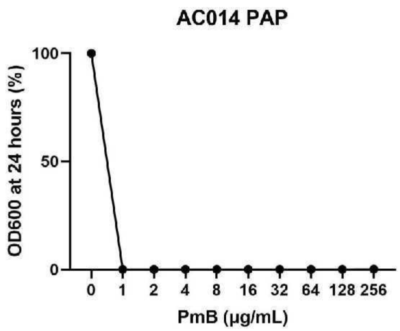
AC014 PAP
100
50
0
OD600 at 24 hours (%)
0
1
2
4
8
16
32
64
128
256
PmB (µg/mL)

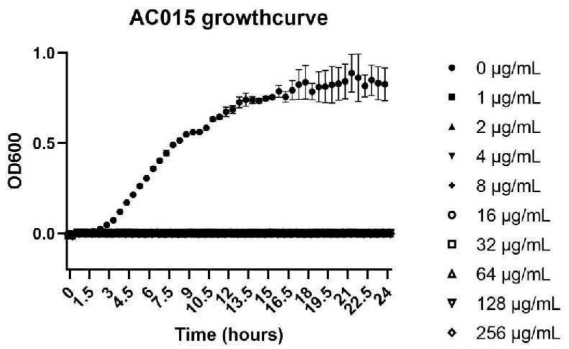
AC015 growthcurve
1.0
0.5
0.0
OD600
0
1.5
3
4.5
6
7.5
9
10.5
12
13.5
15
16.5
18
19.5
21
22.5
24
Time (hours)
0 µg/mL
1 µg/mL
2 µg/mL
4 µg/mL
8 µg/mL
16 µg/mL
32 µg/mL
64 µg/mL
128 µg/mL
256 µg/mL

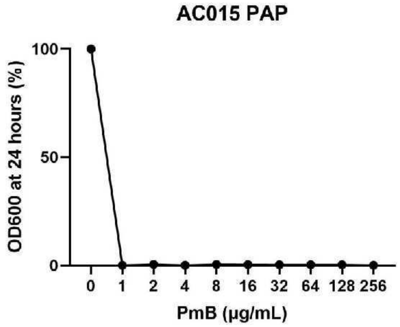
AC015 PAP
100
50
0
OD600 at 24 hours (%)
0
1
2
4
8
16
32
64
128
256
PmB (µg/mL)

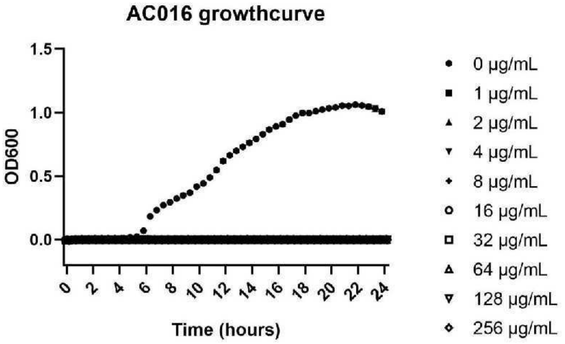
AC016 growthcurve
1.5
1.0
0.5
0.0
OD600
0
2
4
6
8
10
12
14
16
18
20
22
24
Time (hours)
0 μg/mL
1 μg/mL
2 μg/mL
4 μg/mL
8 μg/mL
16 μg/mL
32 μg/mL
64 μg/mL
128 μg/mL
256 μg/mL

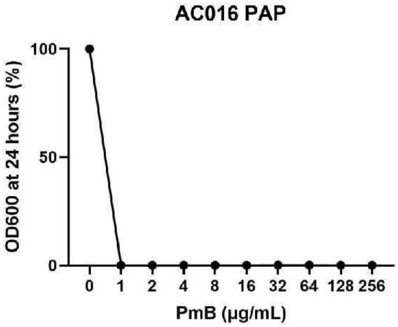
AC016 PAP
100
50
0
OD600 at 24 hours (%)
0
1
2
4
8
16
32
64
128
256
PmB (μg/mL)

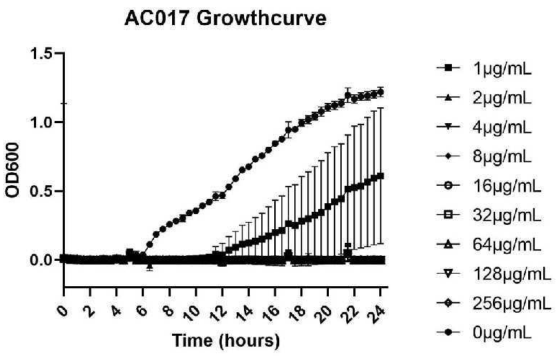
AC017 Growthcurve
1.5
1.0
0.5
0.0
OD600
0
2
4
6
8
10
12
14
16
18
20
22
24
Time (hours)
1μg/mL
2μg/mL
4μg/mL
8μg/mL
16μg/mL
32μg/mL
64μg/mL
128μg/mL
256μg/mL
0μg/mL

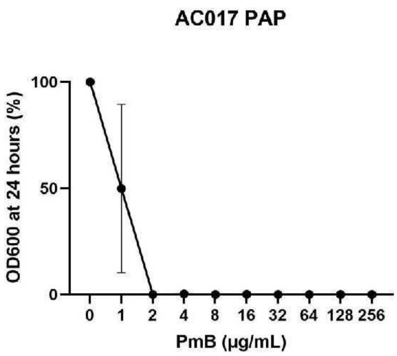
AC017 PAP
100
50
0
OD600 at 24 hours (%)
0
1
2
4
8
16
32
64
128
256
PmB (μg/mL)

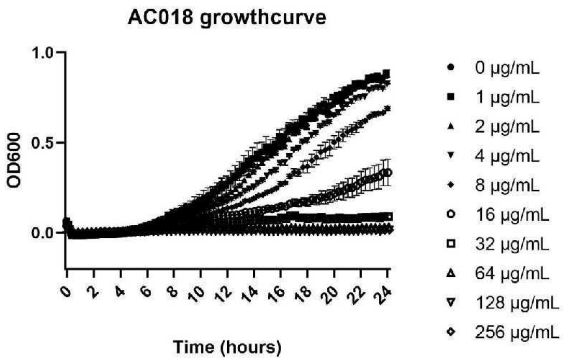
AC018 growthcurve
1.0
0.5
0.0
OD600
0
2
4
6
8
10
12
14
16
18
20
22
24
Time (hours)
0 µg/mL
1 µg/mL
2 µg/mL
4 µg/mL
8 µg/mL
16 µg/mL
32 µg/mL
64 µg/mL
128 µg/mL
256 µg/mL

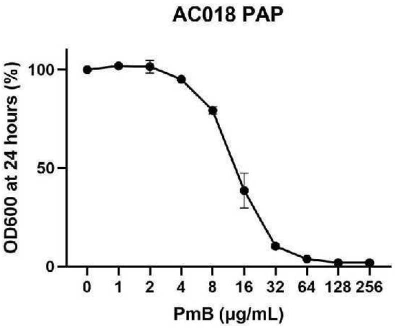
AC018 PAP
100
50
0
OD600 at 24 hours (%)
0
1
2
4
8
16
32
64
128
256
PmB (µg/mL)

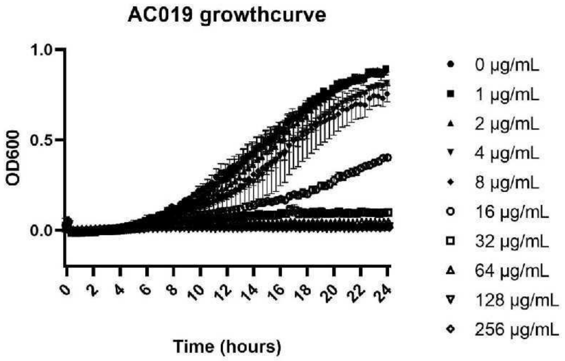
AC019 growthcurve
1.0
0.5
0.0
OD600
0
2
4
6
8
10
12
14
16
18
20
22
24
Time (hours)
0 µg/mL
1 µg/mL
2 µg/mL
4 µg/mL
8 µg/mL
16 µg/mL
32 µg/mL
64 µg/mL
128 µg/mL
256 µg/mL

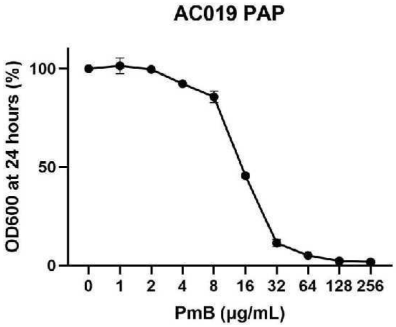
AC019 PAP
OD600 at 24 hours (%)
100
50
0
0
1
2
4
8
16
32
64
128
256
PmB (µg/mL)

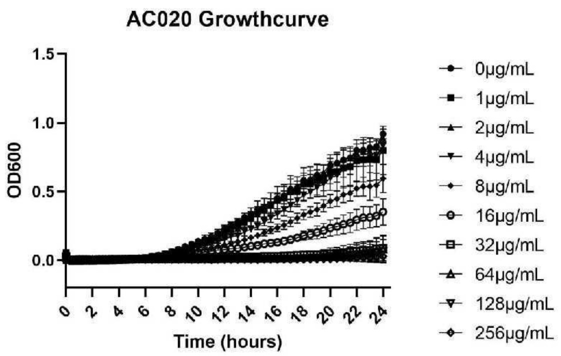
AC020 Growthcurve
1.5
1.0
0.5
0.0
OD600
0
2
4
6
8
10
12
14
16
18
20
22
24
Time (hours)
0μg/mL
1μg/mL
2μg/mL
4μg/mL
8μg/mL
16μg/mL
32μg/mL
64μg/mL
128μg/mL
256μg/mL

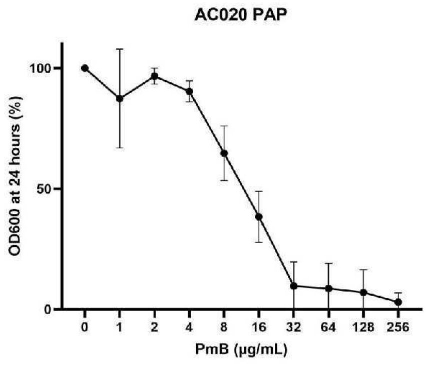
AC020 PAP
100
50
0
OD600 at 24 hours (%)
0
1
2
4
8
16
32
64
128
256
PmB (μg/mL)

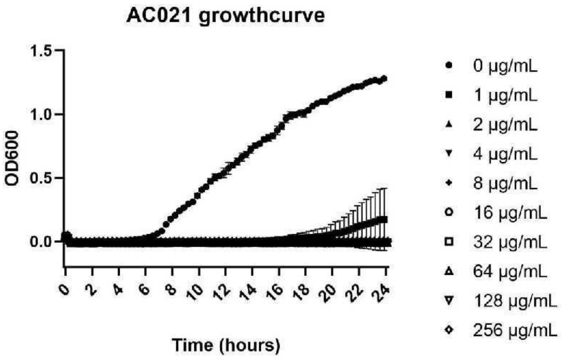
AC021 growthcurve
1.5
1.0
0.5
0.0
OD600
0
2
4
6
8
10
12
14
16
18
20
22
24
Time (hours)
0 μg/mL
1 μg/mL
2 μg/mL
4 μg/mL
8 μg/mL
16 μg/mL
32 μg/mL
64 μg/mL
128 μg/mL
256 μg/mL

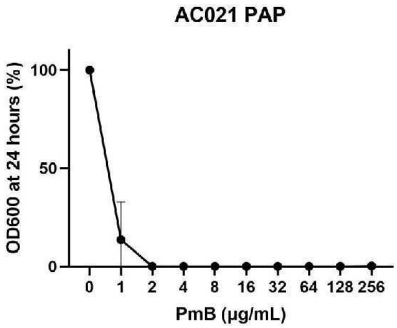
AC021 PAP
100
50
0
OD600 at 24 hours (%)
0
1
2
4
8
16
32
64
128
256
PmB (μg/mL)

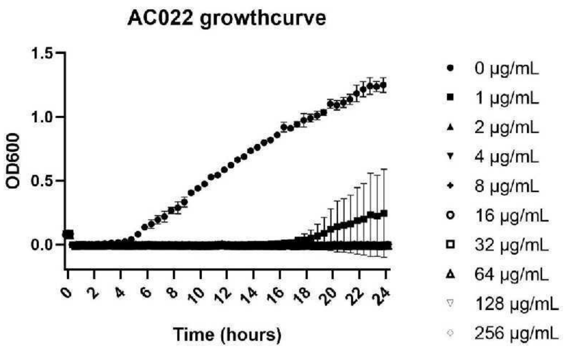
AC022 growthcurve
1.5
1.0
0.5
0.0
OD600
0
2
4
6
8
10
12
14
16
18
20
22
24
Time (hours)
0 µg/mL
1 µg/mL
2 µg/mL
4 µg/mL
8 µg/mL
16 µg/mL
32 µg/mL
64 µg/mL
128 µg/mL
256 µg/mL

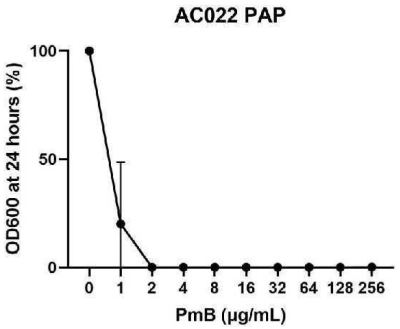
AC022 PAP
100
50
0
OD600 at 24 hours (%)
0
1
2
4
8
16
32
64
128
256
PmB (µg/mL)

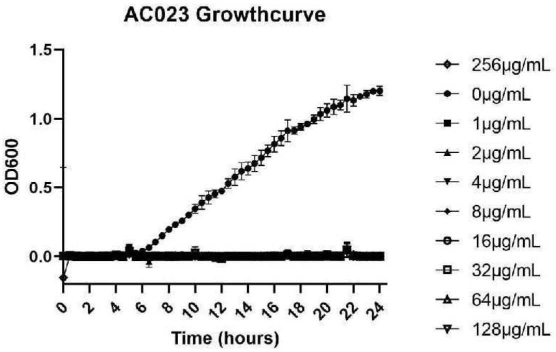
AC023 Growthcurve
1.5
1.0
0.5
0.0
OD600
0
2
4
6
8
10
12
14
16
18
20
22
24
Time (hours)
256µg/mL
0µg/mL
1µg/mL
2µg/mL
4µg/mL
8µg/mL
16µg/mL
32µg/mL
64µg/mL
128µg/mL

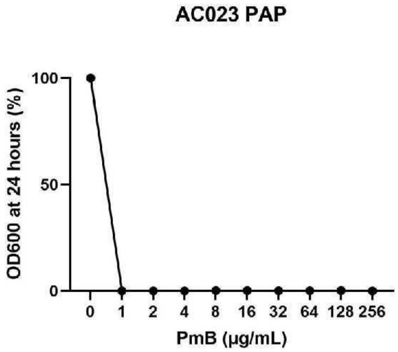
AC023 PAP
100
50
0
OD600 at 24 hours (%)
0
1
2
4
8
16
32
64
128
256
PmB (µg/mL)

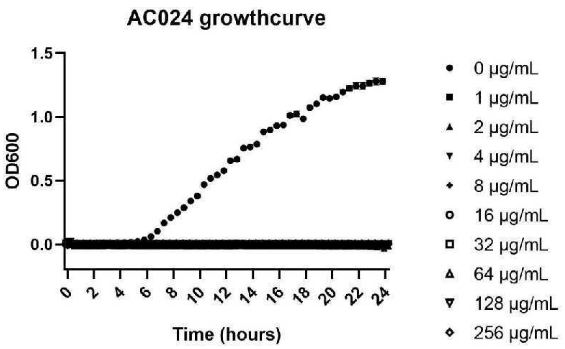
AC024 growthcurve
1.5
1.0
0.5
0.0
OD600
0
2
4
6
8
10
12
14
16
18
20
22
24
Time (hours)
0 µg/mL
1 µg/mL
2 µg/mL
4 µg/mL
8 µg/mL
16 µg/mL
32 µg/mL
64 µg/mL
128 µg/mL
256 µg/mL

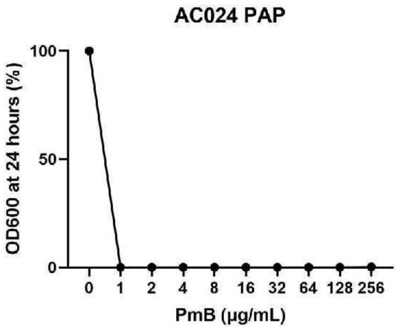
AC024 PAP
100
50
0
OD600 at 24 hours (%)
0
1
2
4
8
16
32
64
128
256
PmB (µg/mL)

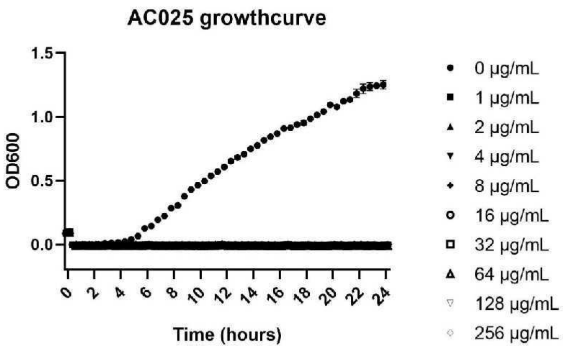
AC025 growthcurve
1.5
1.0
0.5
0.0
OD600
0
2
4
6
8
10
12
14
16
18
20
22
24
Time (hours)
0 µg/mL
1 µg/mL
2 µg/mL
4 µg/mL
8 µg/mL
16 µg/mL
32 µg/mL
64 µg/mL
128 µg/mL
256 µg/mL

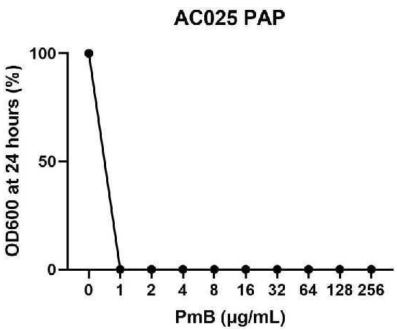
AC025 PAP
100
50
0
OD600 at 24 hours (%)
0
1
2
4
8
16
32
64
128
256
PmB (µg/mL)

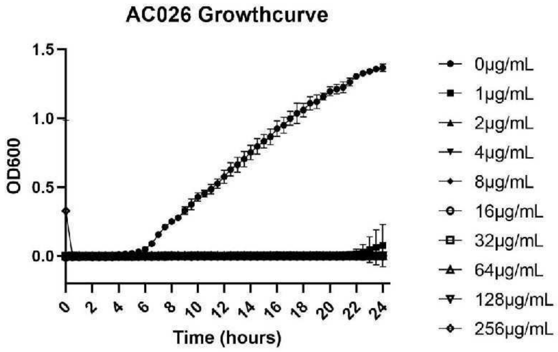
AC026 Growthcurve
1.5
1.0
0.5
0.0
OD600
0
2
4
6
8
10
12
14
16
18
20
22
24
Time (hours)
0µg/mL
1µg/mL
2µg/mL
4µg/mL
8µg/mL
16µg/mL
32µg/mL
64µg/mL
128µg/mL
256µg/mL

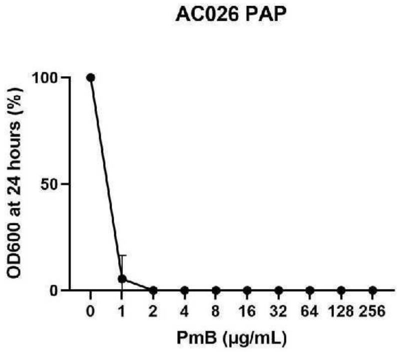
AC026 PAP
100
50
0
OD600 at 24 hours (%)
0
1
2
4
8
16
32
64
128
256
PmB (µg/mL)

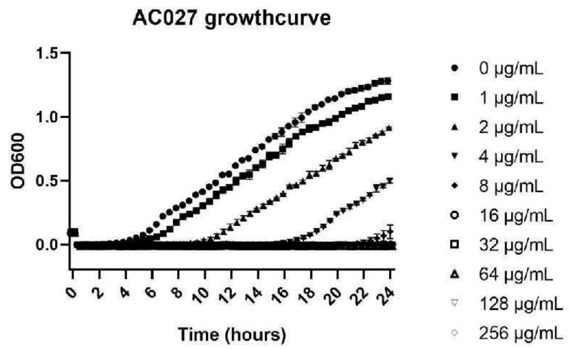
AC027 growthcurve
1.5
1.0
0.5
0.0
OD600
0
2
4
6
8
10
12
14
16
18
20
22
24
Time (hours)
0 µg/mL
1 µg/mL
2 µg/mL
4 µg/mL
8 µg/mL
16 µg/mL
32 µg/mL
64 µg/mL
128 µg/mL
256 µg/mL

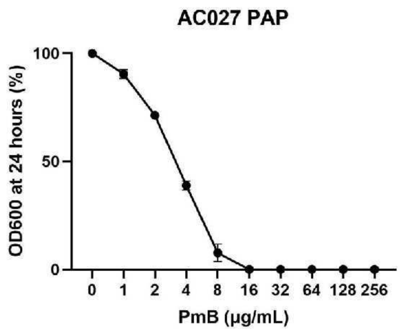
AC027 PAP
100
50
0
OD600 at 24 hours (%)
0
1
2
4
8
16
32
64
128
256
PmB (µg/mL)

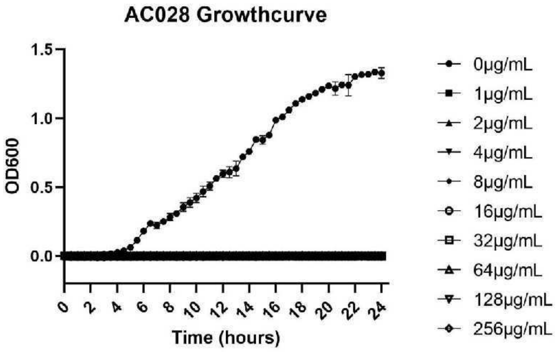
AC028 Growthcurve
1.5
1.0
0.5
0.0
OD600
0
2
4
6
8
10
12
14
16
18
20
22
24
Time (hours)
0μg/mL
1μg/mL
2μg/mL
4μg/mL
8μg/mL
16μg/mL
32μg/mL
64μg/mL
128μg/mL
256μg/mL

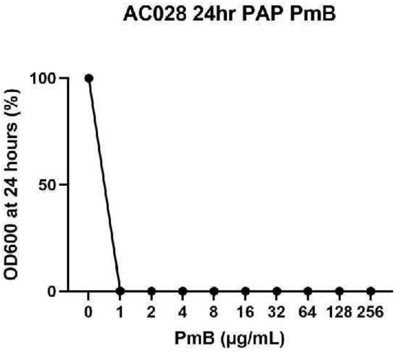
AC028 24hr PAP PmB
100
50
0
OD600 at 24 hours (%)
0
1
2
4
8
16
32
64
128
256
PmB (μg/mL)

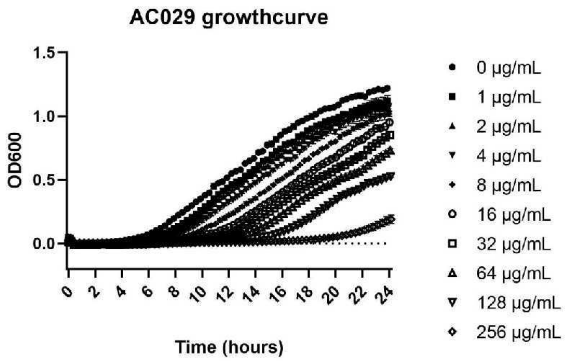
AC029 growthcurve
1.5
1.0
0.5
0.0
OD600
0
2
4
6
8
10
12
14
16
18
20
22
24
Time (hours)
0 μg/mL
1 μg/mL
2 μg/mL
4 μg/mL
8 μg/mL
16 μg/mL
32 μg/mL
64 μg/mL
128 μg/mL
256 μg/mL

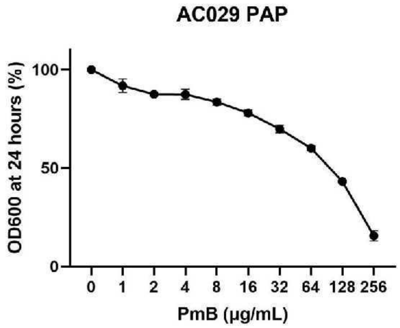
AC029 PAP
100
50
0
OD600 at 24 hours (%)
0
1
2
4
8
16
32
64
128
256
PmB (μg/mL)

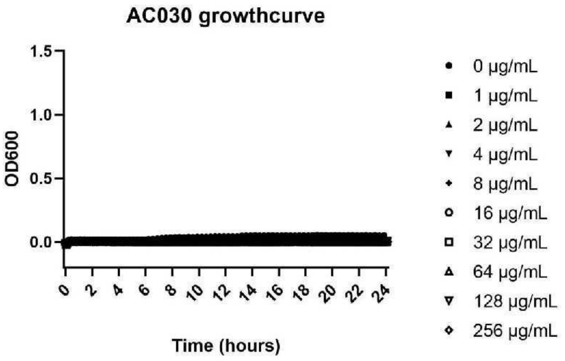
AC030 growthcurve
1.5
1.0
0.5
0.0
OD600
0
2
4
6
8
10
12
14
16
18
20
22
24
Time (hours)
0 µg/mL
1 µg/mL
2 µg/mL
4 µg/mL
8 µg/mL
16 µg/mL
32 µg/mL
64 µg/mL
128 µg/mL
256 µg/mL

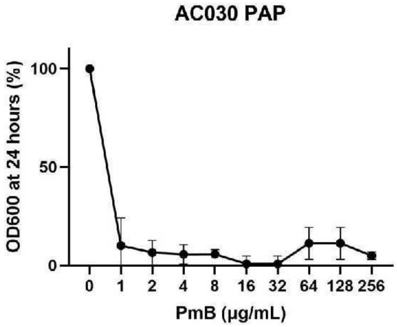
AC030 PAP
100
50
0
OD600 at 24 hours (%)
0
1
2
4
8
16
32
64
128
256
PmB (µg/mL)

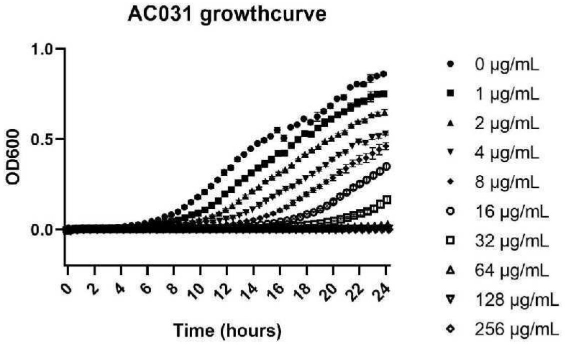
AC031 growthcurve
1.0
0.5
0.0
OD600
0
2
4
6
8
10
12
14
16
18
20
22
24
Time (hours)
0 µg/mL
1 µg/mL
2 µg/mL
4 µg/mL
8 µg/mL
16 µg/mL
32 µg/mL
64 µg/mL
128 µg/mL
256 µg/mL

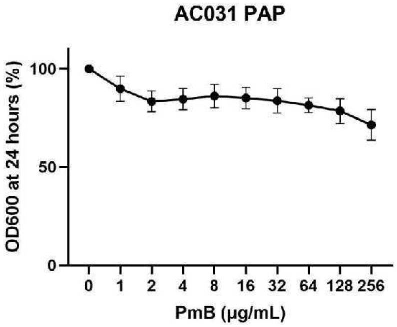
AC031 PAP
OD600 at 24 hours (%)
100
50
0
0
1
2
4
8
16
32
64
128
256
PmB (µg/mL)

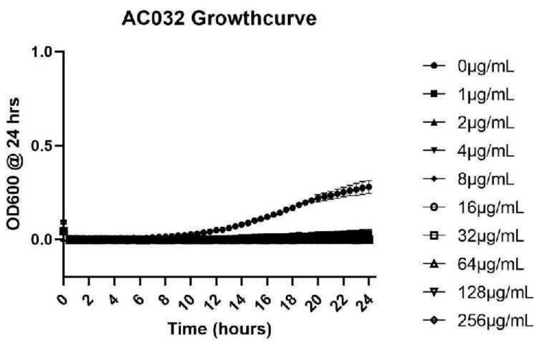
AC032 Growthcurve
1.0
0.5
0.0
OD600 @ 24 hrs
0
2
4
6
8
10
12
14
16
18
20
22
24
Time (hours)
0µg/mL
1µg/mL
2µg/mL
4µg/mL
8µg/mL
16µg/mL
32µg/mL
64µg/mL
128µg/mL
256µg/mL

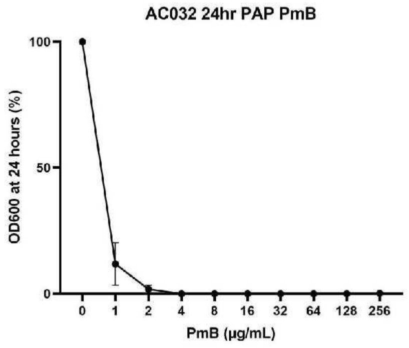
AC032 24hr PAP PmB
100
50
0
OD600 at 24 hours (%)
0
1
2
4
8
16
32
64
128
256
PmB (µg/mL)

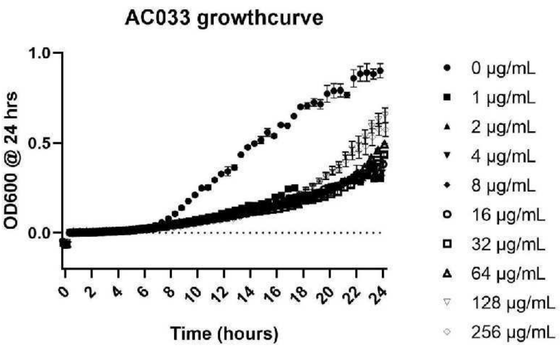
AC033 growthcurve
1.0
0.5
0.0
OD600 @ 24 hrs
0
2
4
6
8
10
12
14
16
18
20
22
24
Time (hours)
0 µg/mL
1 µg/mL
2 µg/mL
4 µg/mL
8 µg/mL
16 µg/mL
32 µg/mL
64 µg/mL
128 µg/mL
256 µg/mL

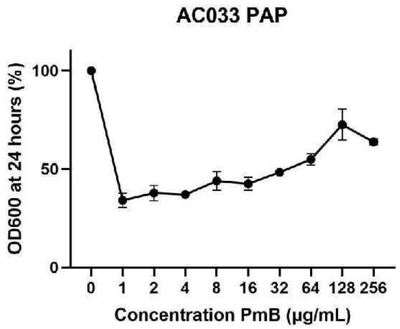
AC033 PAP
100
50
0
OD600 at 24 hours (%)
0
1
2
4
8
16
32
64
128
256
Concentration PmB (µg/mL)

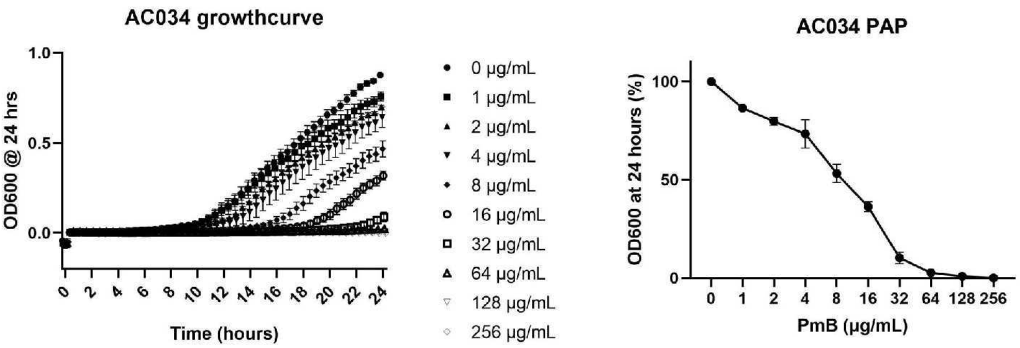

AC034 growthcurve
1.0
0.5
0.0
OD600 @ 24 hrs
0
2
4
6
8
10
12
14
16
18
20
22
24
Time (hours)
0 μg/mL
1 μg/mL
2 μg/mL
4 μg/mL
8 μg/mL
16 μg/mL
32 μg/mL
64 μg/mL
128 μg/mL
256 μg/mL
AC034 PAP
100
50
0
OD600 at 24 hours (%)
0
1
2
4
8
16
32
64
128
256
PmB (μg/mL)

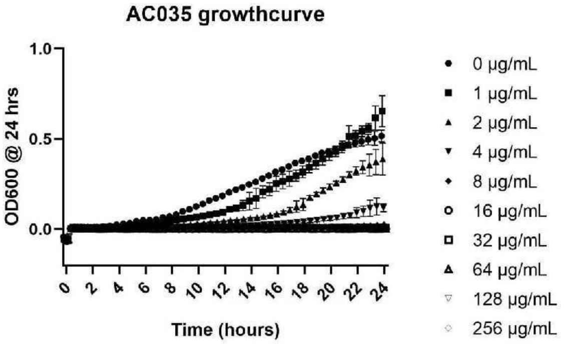
AC035 growthcurve
1.0
0.5
0.0
OD600 @ 24 hrs
0 2 4 6 8 10 12 14 16 18 20 22 24
Time (hours)
0 μg/mL
1 μg/mL
2 μg/mL
4 μg/mL
8 μg/mL
16 μg/mL
32 μg/mL
64 μg/mL
128 μg/mL
256 μg/mL

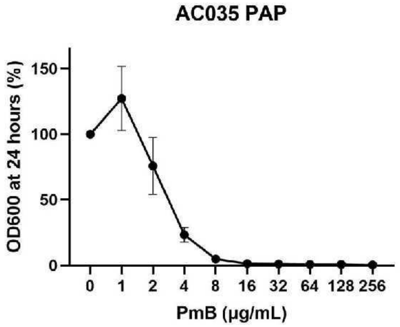
AC035 PAP
150
100
50
0
OD600 at 24 hours (%)
0 1 2 4 8 16 32 64 128 256
PmB (μg/mL)

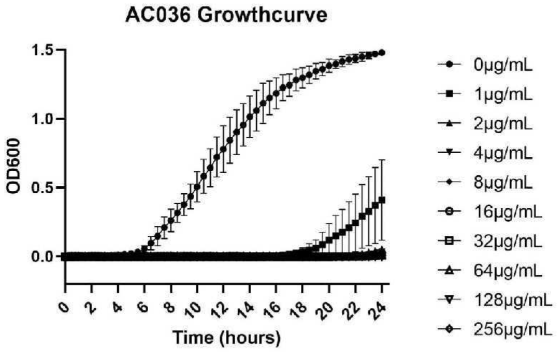
AC036 Growthcurve
1.5
1.0
0.5
0.0
OD600
0
2
4
6
8
10
12
14
16
18
20
22
24
Time (hours)
0μg/mL
1μg/mL
2μg/mL
4μg/mL
8μg/mL
16μg/mL
32μg/mL
64μg/mL
128μg/mL
256μg/mL

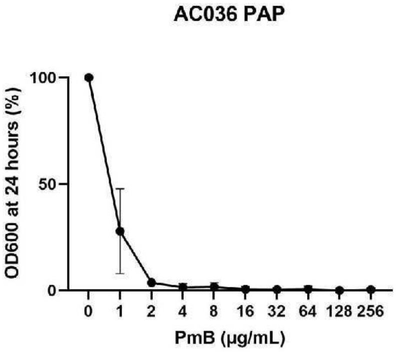
AC036 PAP
100
50
0
OD600 at 24 hours (%)
0
1
2
4
8
16
32
64
128
256
PmB (μg/mL)

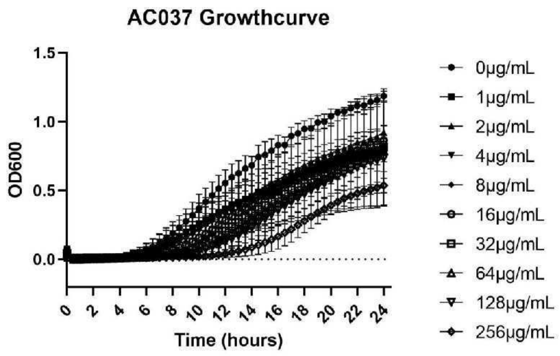
AC037 Growthcurve
OD600
1.5
1.0
0.5
0.0
0
2
4
6
8
10
12
14
16
18
20
22
24
Time (hours)
0μg/mL
1μg/mL
2μg/mL
4μg/mL
8μg/mL
16μg/mL
32μg/mL
64μg/mL
128μg/mL
256μg/mL

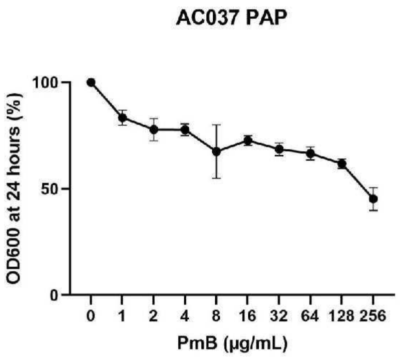
AC037 PAP
OD600 at 24 hours (%)
100
50
0
0
1
2
4
8
16
32
64
128
256
PmB (μg/mL)

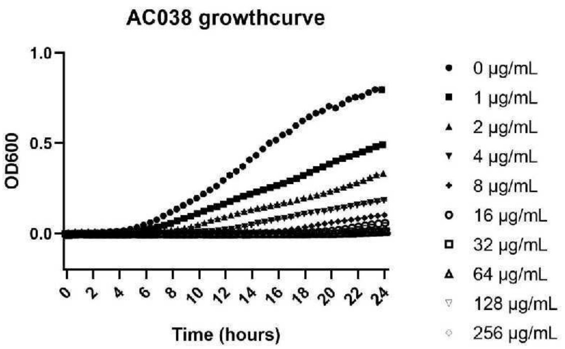
AC038 growthcurve
1.0
0.5
0.0
OD600
0
2
4
6
8
10
12
14
16
18
20
22
24
Time (hours)
0 μg/mL
1 μg/mL
2 μg/mL
4 μg/mL
8 μg/mL
16 μg/mL
32 μg/mL
64 μg/mL
128 μg/mL
256 μg/mL

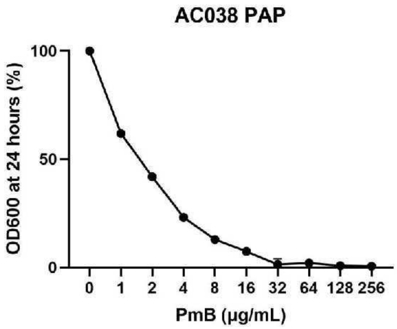
AC038 PAP
100
50
0
OD600 at 24 hours (%)
0
1
2
4
8
16
32
64
128
256
PmB (μg/mL)

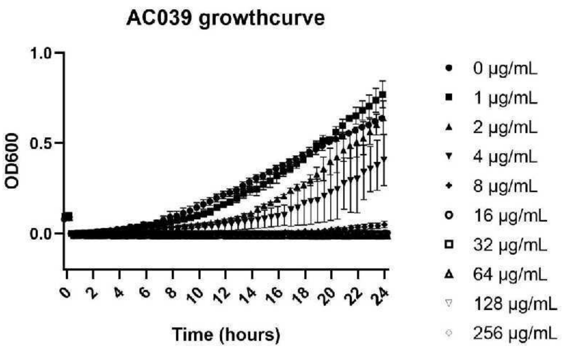
AC039 growthcurve
1.0
0.5
0.0
OD600
0
2
4
6
8
10
12
14
16
18
20
22
24
Time (hours)
0 µg/mL
1 µg/mL
2 µg/mL
4 µg/mL
8 µg/mL
16 µg/mL
32 µg/mL
64 µg/mL
128 µg/mL
256 µg/mL

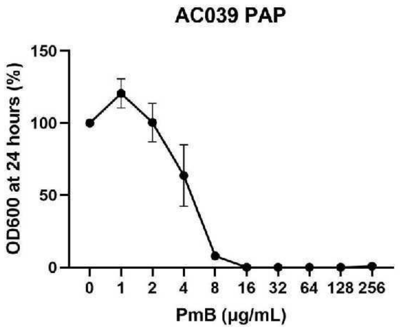
AC039 PAP
150
100
50
0
OD600 at 24 hours (%)
0
1
2
4
8
16
32
64
128
256
PmB (µg/mL)

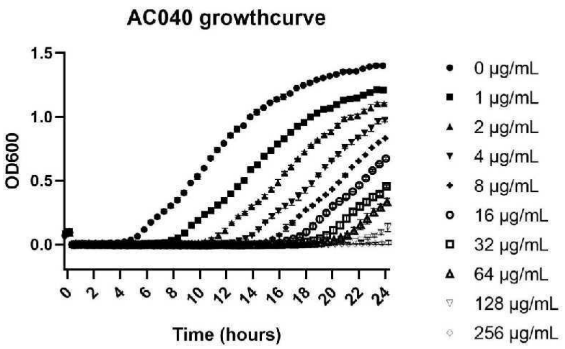
AC040 growthcurve
1.5
1.0
0.5
0.0
OD600
0
2
4
6
8
10
12
14
16
18
20
22
24
Time (hours)
0 µg/mL
1 µg/mL
2 µg/mL
4 µg/mL
8 µg/mL
16 µg/mL
32 µg/mL
64 µg/mL
128 µg/mL
256 µg/mL

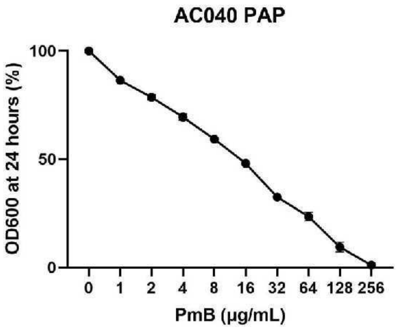
AC040 PAP
100
50
0
OD600 at 24 hours (%)
0
1
2
4
8
16
32
64
128
256
PmB (µg/mL)

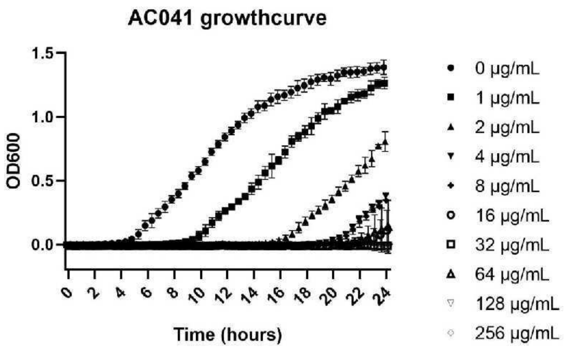
AC041 growthcurve
OD600
1.5
1.0
0.5
0.0
0
2
4
6
8
10
12
14
16
18
20
22
24
Time (hours)
0 µg/mL
1 µg/mL
2 µg/mL
4 µg/mL
8 µg/mL
16 µg/mL
32 µg/mL
64 µg/mL
128 µg/mL
256 µg/mL

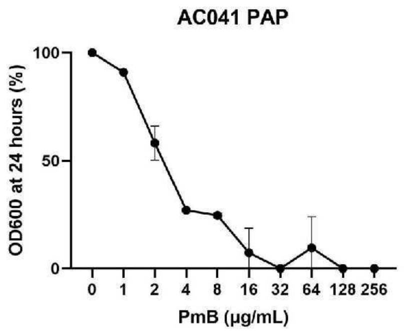
AC041 PAP
OD600 at 24 hours (%)
100
50
0
0
1
2
4
8
16
32
64
128
256
PmB (µg/mL)

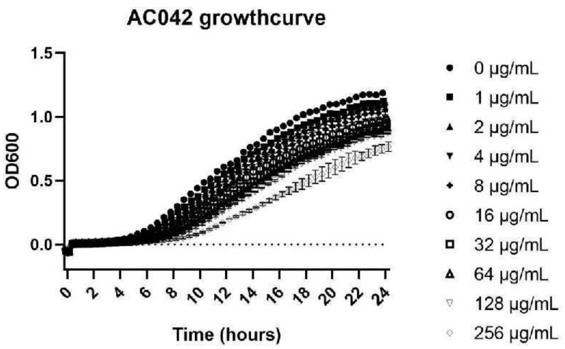
AC042 growthcurve
1.5
1.0
0.5
0.0
OD600
0
2
4
6
8
10
12
14
16
18
20
22
24
Time (hours)
0 µg/mL
1 µg/mL
2 µg/mL
4 µg/mL
8 µg/mL
16 µg/mL
32 µg/mL
64 µg/mL
128 µg/mL
256 µg/mL

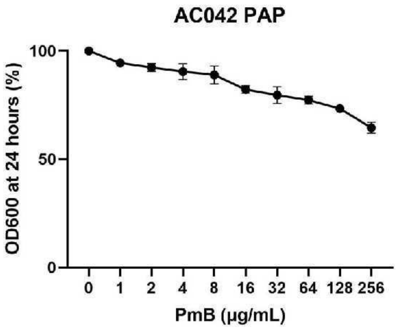
AC042 PAP
100
50
0
OD600 at 24 hours (%)
0
1
2
4
8
16
32
64
128
256
PmB (µg/mL)

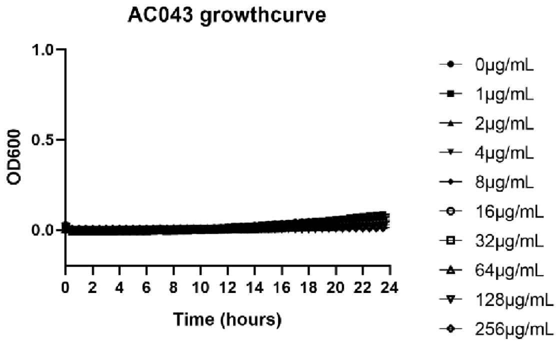
AC043 growthcurve
1.0
0.5
0.0
OD600
0
2
4
6
8
10
12
14
16
18
20
22
24
Time (hours)
0μg/mL
1μg/mL
2μg/mL
4μg/mL
8μg/mL
16μg/mL
32μg/mL
64μg/mL
128μg/mL
256μg/mL

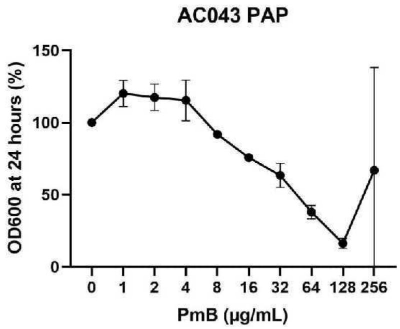
AC043 PAP
150
100
50
0
OD600 at 24 hours (%)
0
1
2
4
8
16
32
64
128
256
PmB (μg/mL)

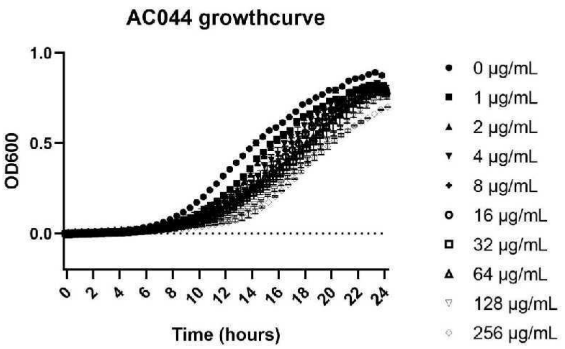

AC044 growthcurve
1.0
0.5
0.0
OD600
0
2
4
6
8
10
12
14
16
18
20
22
24
Time (hours)
0 µg/mL
1 µg/mL
2 µg/mL
4 µg/mL
8 µg/mL
16 µg/mL
32 µg/mL
64 µg/mL
128 µg/mL
256 µg/mL

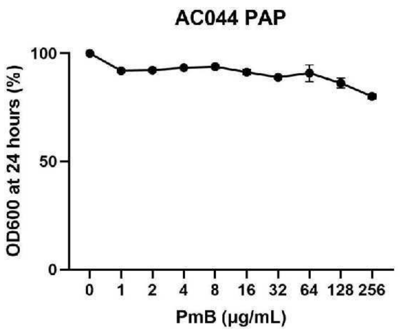

AC044 PAP
100
50
0
OD600 at 24 hours (%)
0
1
2
4
8
16
32
64
128
256
PmB (µg/mL)

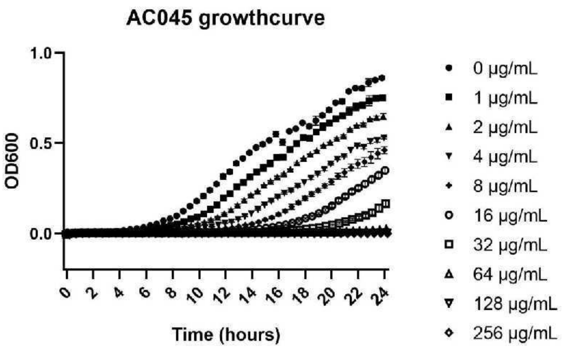
AC045 growthcurve
1.0
0.5
0.0
OD600
0
2
4
6
8
10
12
14
16
18
20
22
24
Time (hours)
0 µg/mL
1 µg/mL
2 µg/mL
4 µg/mL
8 µg/mL
16 µg/mL
32 µg/mL
64 µg/mL
128 µg/mL
256 µg/mL

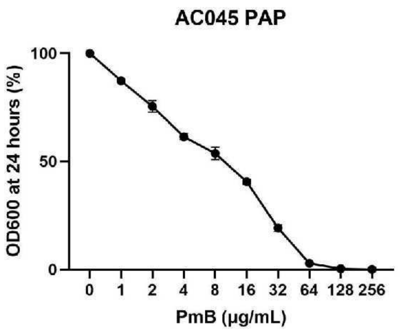
AC045 PAP
100
50
0
OD600 at 24 hours (%)
0
1
2
4
8
16
32
64
128
256
PmB (µg/mL)

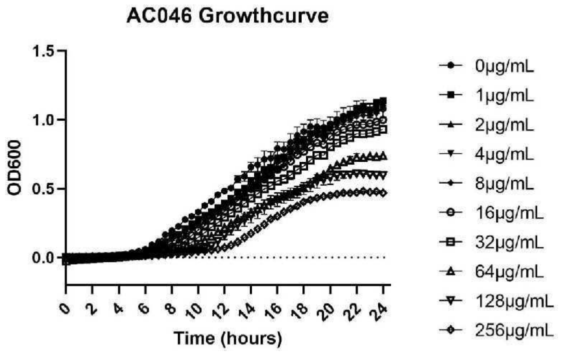
AC046 Growthcurve
1.5
1.0
0.5
0.0
OD600
0
2
4
6
8
10
12
14
16
18
20
22
24
Time (hours)
0μg/mL
1μg/mL
2μg/mL
4μg/mL
8μg/mL
16μg/mL
32μg/mL
64μg/mL
128μg/mL
256μg/mL

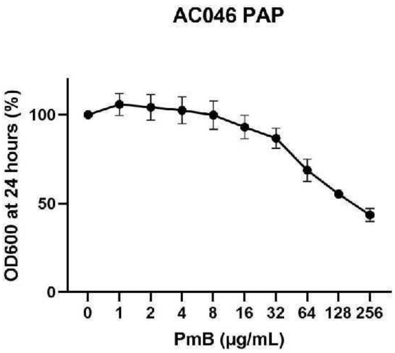
AC046 PAP
OD600 at 24 hours (%)
100
50
0
0
1
2
4
8
16
32
64
128
256
PmB (μg/mL)

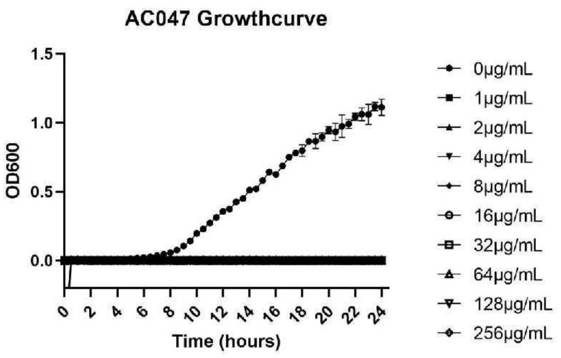
AC047 Growthcurve
1.5
1.0
0.5
0.0
OD600
0 2 4 6 8 10 12 14 16 18 20 22 24
Time (hours)
0µg/mL
1µg/mL
2µg/mL
4µg/mL
8µg/mL
16µg/mL
32µg/mL
64µg/mL
128µg/mL
256µg/mL

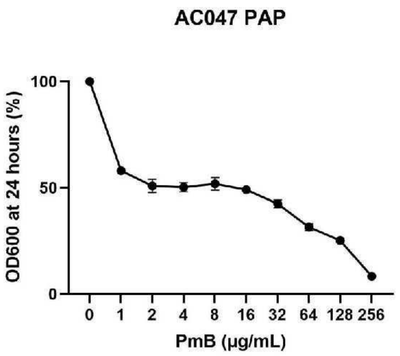
AC047 PAP
100
50
0
OD600 at 24 hours (%)
0 1 2 4 8 16 32 64 128 256
PmB (µg/mL)

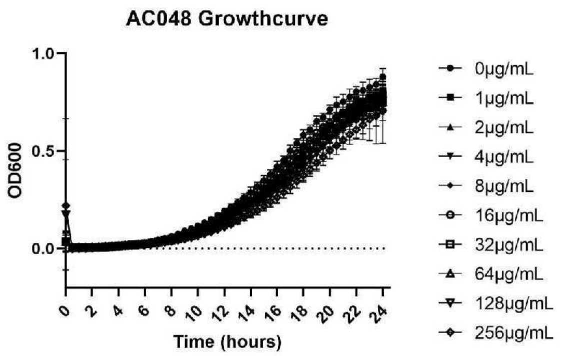

AC048 Growthcurve
1.0
0.5
0.0
OD600
0
2
4
6
8
10
12
14
16
18
20
22
24
Time (hours)
0μg/mL
1μg/mL
2μg/mL
4μg/mL
8μg/mL
16μg/mL
32μg/mL
64μg/mL
128μg/mL
256μg/mL

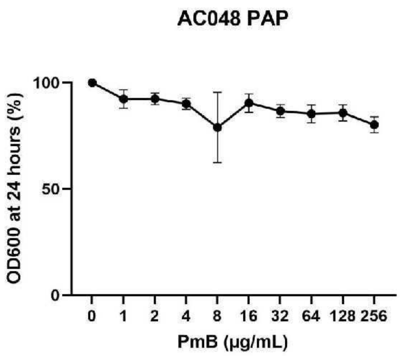

AC048 PAP
100
50
0
OD600 at 24 hours (%)
0
1
2
4
8
16
32
64
128
256
PmB (μg/mL)

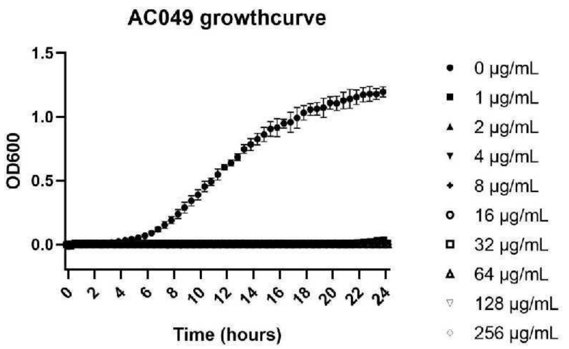
AC049 growthcurve
OD600
Time (hours)
0 µg/mL
1 µg/mL
2 µg/mL
4 µg/mL
8 µg/mL
16 µg/mL
32 µg/mL
64 µg/mL
128 µg/mL
256 µg/mL

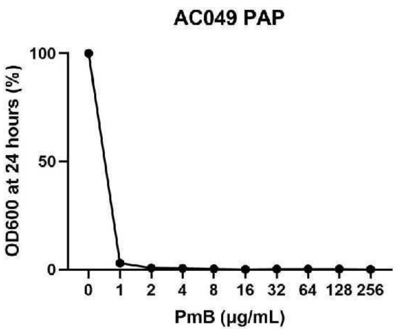
AC049 PAP
OD600 at 24 hours (%)
PmB (µg/mL)

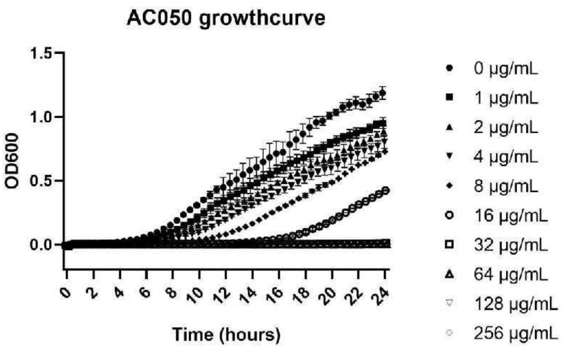
AC050 growthcurve
1.5
1.0
0.5
0.0
OD600
0
2
4
6
8
10
12
14
16
18
20
22
24
Time (hours)
0 μg/mL
1 μg/mL
2 μg/mL
4 μg/mL
8 μg/mL
16 μg/mL
32 μg/mL
64 μg/mL
128 μg/mL
256 μg/mL

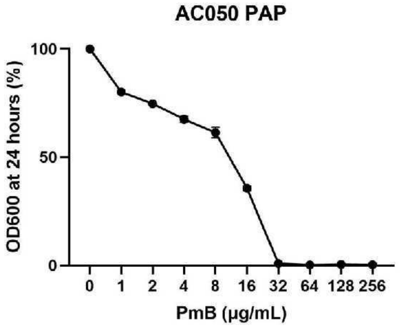
AC050 PAP
100
50
0
OD600 at 24 hours (%)
0
1
2
4
8
16
32
64
128
256
PmB (μg/mL)

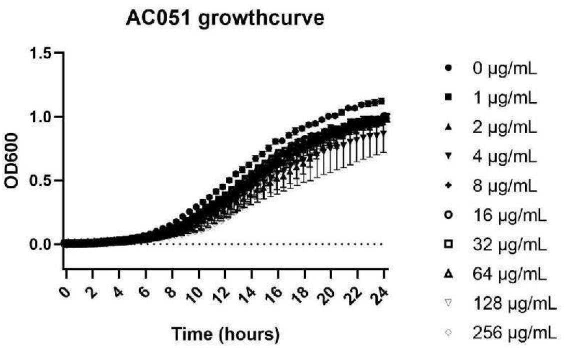
AC051 growthcurve
1.5
1.0
0.5
0.0
OD600
0
2
4
6
8
10
12
14
16
18
20
22
24
Time (hours)
0 µg/mL
1 µg/mL
2 µg/mL
4 µg/mL
8 µg/mL
16 µg/mL
32 µg/mL
64 µg/mL
128 µg/mL
256 µg/mL

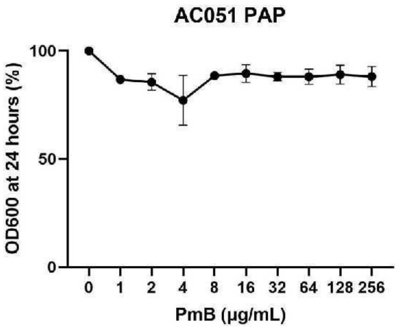
AC051 PAP
100
50
0
OD600 at 24 hours (%)
0
1
2
4
8
16
32
64
128
256
PmB (µg/mL)

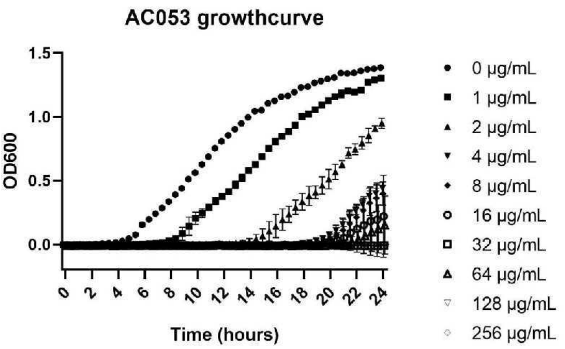
AC053 growthcurve
1.5
1.0
0.5
0.0
OD600
0
2
4
6
8
10
12
14
16
18
20
22
24
Time (hours)
0 µg/mL
1 µg/mL
2 µg/mL
4 µg/mL
8 µg/mL
16 µg/mL
32 µg/mL
64 µg/mL
128 µg/mL
256 µg/mL

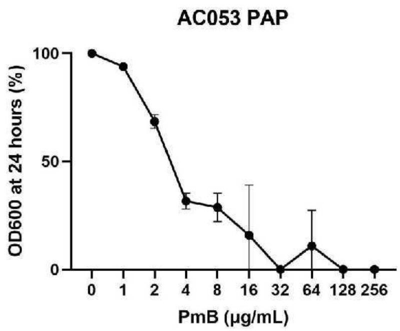
AC053 PAP
100
50
0
OD600 at 24 hours (%)
0
1
2
4
8
16
32
64
128
256
PmB (µg/mL)

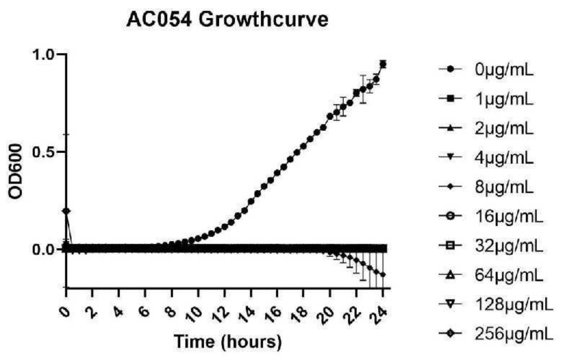
AC054 Growthcurve
OD600
1.0
0.5
0.0
0
2
4
6
8
10
12
14
16
18
20
22
24
Time (hours)
0μg/mL
1μg/mL
2μg/mL
4μg/mL
8μg/mL
16μg/mL
32μg/mL
64μg/mL
128μg/mL
256μg/mL

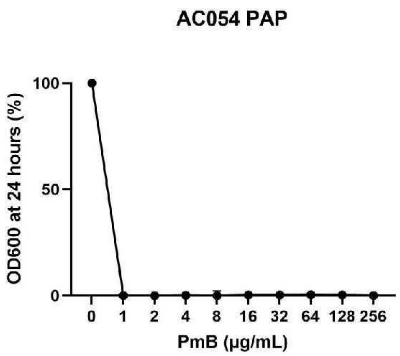
AC054 PAP
OD600 at 24 hours (%)
100
50
0
0
1
2
4
8
16
32
64
128
256
PmB (μg/mL)

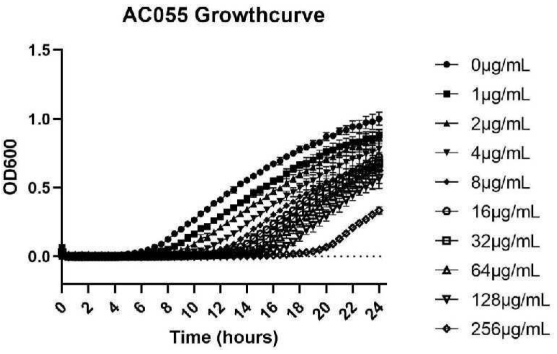
AC055 Growthcurve
1.5
1.0
0.5
0.0
OD600
0
2
4
6
8
10
12
14
16
18
20
22
24
Time (hours)
0μg/mL
1μg/mL
2μg/mL
4μg/mL
8μg/mL
16μg/mL
32μg/mL
64μg/mL
128μg/mL
256μg/mL

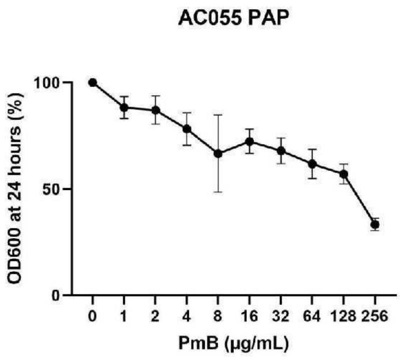
AC055 PAP
100
50
0
OD600 at 24 hours (%)
0
1
2
4
8
16
32
64
128
256
PmB (μg/mL)

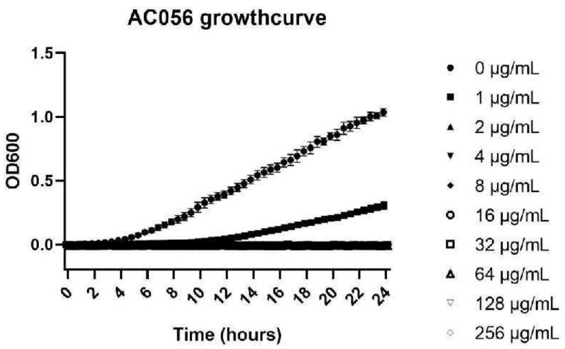
AC056 growthcurve
OD600
1.5
1.0
0.5
0.0
0 2 4 6 8 10 12 14 16 18 20 22 24
Time (hours)
0 µg/mL
1 µg/mL
2 µg/mL
4 µg/mL
8 µg/mL
16 µg/mL
32 µg/mL
64 µg/mL
128 µg/mL
256 µg/mL

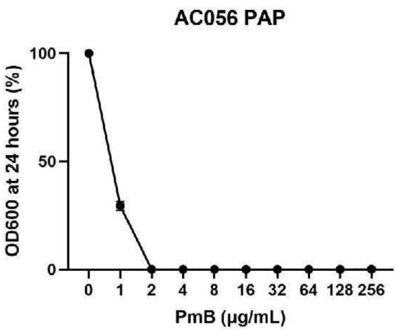
AC056 PAP
OD600 at 24 hours (%)
100
50
0
0 1 2 4 8 16 32 64 128 256
PmB (µg/mL)

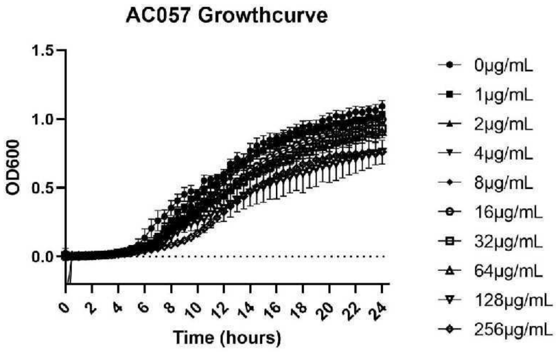
AC057 Growthcurve
1.5
1.0
0.5
0.0
OD600
0
2
4
6
8
10
12
14
16
18
20
22
24
Time (hours)
0µg/mL
1µg/mL
2µg/mL
4µg/mL
8µg/mL
16µg/mL
32µg/mL
64µg/mL
128µg/mL
256µg/mL

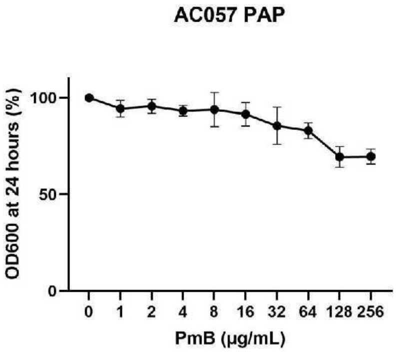
AC057 PAP
100
50
0
OD600 at 24 hours (%)
0
1
2
4
8
16
32
64
128
256
PmB (µg/mL)

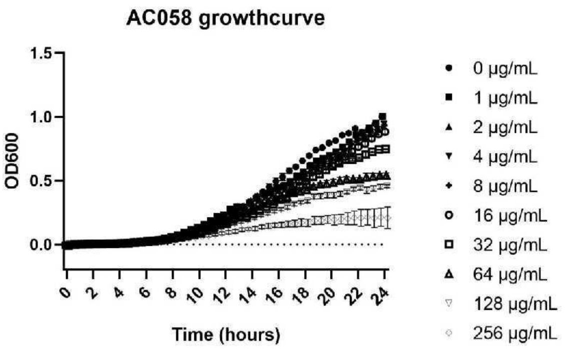
AC058 growthcurve
1.5
1.0
0.5
0.0
OD600
0
2
4
6
8
10
12
14
16
18
20
22
24
Time (hours)
0 µg/mL
1 µg/mL
2 µg/mL
4 µg/mL
8 µg/mL
16 µg/mL
32 µg/mL
64 µg/mL
128 µg/mL
256 µg/mL

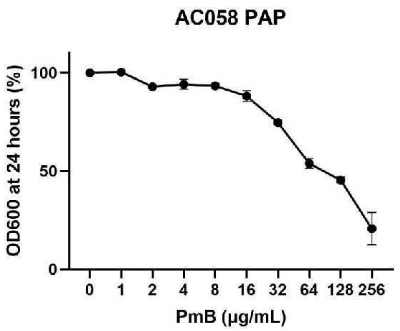
AC058 PAP
100
50
0
OD600 at 24 hours (%)
0
1
2
4
8
16
32
64
128
256
PmB (µg/mL)

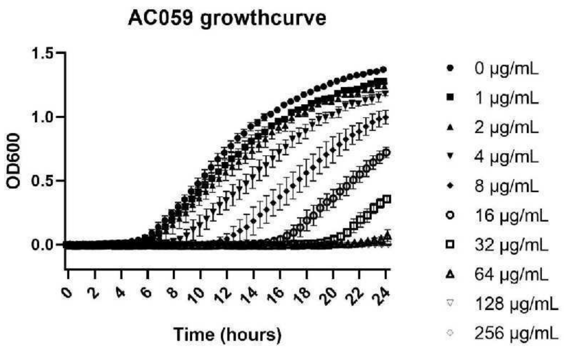
AC059 growthcurve
1.5
1.0
0.5
0.0
OD600
0
2
4
6
8
10
12
14
16
18
20
22
24
Time (hours)
0 µg/mL
1 µg/mL
2 µg/mL
4 µg/mL
8 µg/mL
16 µg/mL
32 µg/mL
64 µg/mL
128 µg/mL
256 µg/mL

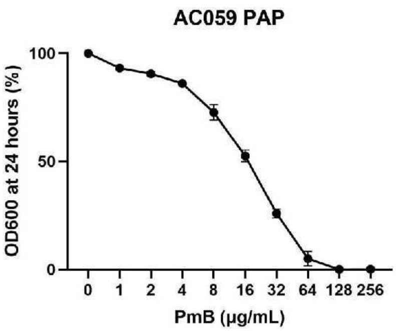
AC059 PAP
100
50
0
OD600 at 24 hours (%)
0
1
2
4
8
16
32
64
128
256
PmB (µg/mL)

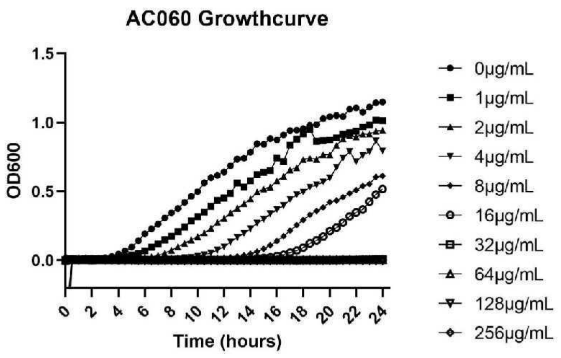
AC060 Growthcurve
1.5
1.0
0.5
0.0
OD600
0 2 4 6 8 10 12 14 16 18 20 22 24
Time (hours)
0μg/mL
1μg/mL
2μg/mL
4μg/mL
8μg/mL
16μg/mL
32μg/mL
64μg/mL
128μg/mL
256μg/mL

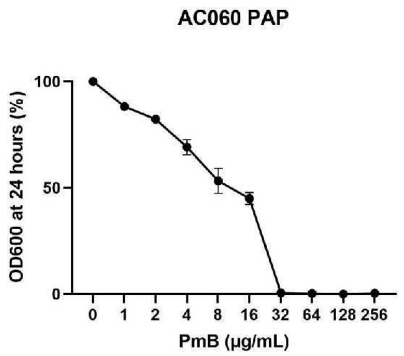
AC060 PAP
100
50
0
OD600 at 24 hours (%)
0 1 2 4 8 16 32 64 128 256
PmB (μg/mL)

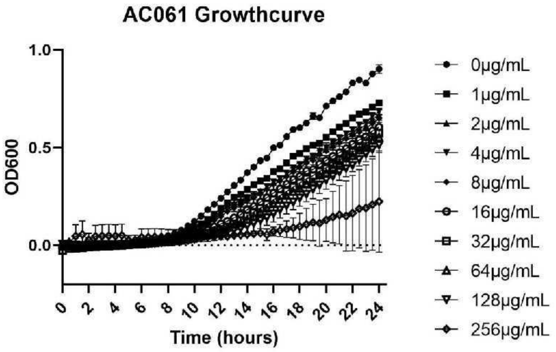
AC061 Growthcurve
1.0
0.5
0.0
OD600
0
2
4
6
8
10
12
14
16
18
20
22
24
Time (hours)
0µg/mL
1µg/mL
2µg/mL
4µg/mL
8µg/mL
16µg/mL
32µg/mL
64µg/mL
128µg/mL
256µg/mL

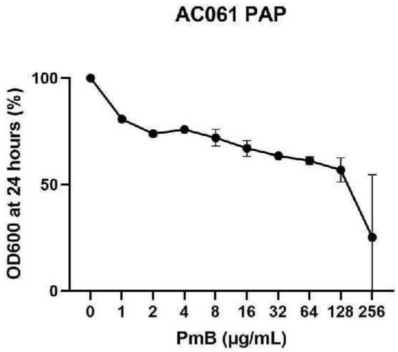
AC061 PAP
100
50
0
OD600 at 24 hours (%)
0
1
2
4
8
16
32
64
128
256
PmB (µg/mL)

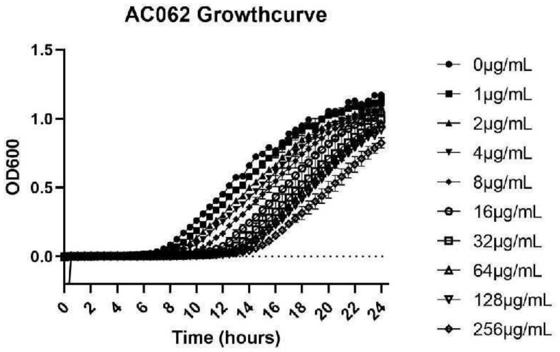
AC062 Growthcurve
1.5
1.0
0.5
0.0
OD600
0
2
4
6
8
10
12
14
16
18
20
22
24
Time (hours)
0μg/mL
1μg/mL
2μg/mL
4μg/mL
8μg/mL
16μg/mL
32μg/mL
64μg/mL
128μg/mL
256μg/mL

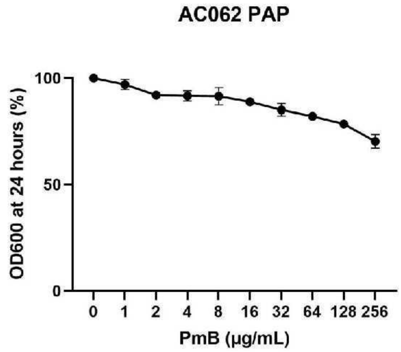
AC062 PAP
100
50
0
OD600 at 24 hours (%)
0
1
2
4
8
16
32
64
128
256
PmB (μg/mL)

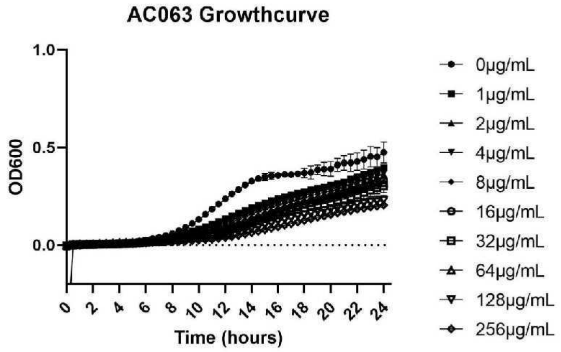

AC063 Growthcurve
OD600
1.0
0.5
0.0
0 2 4 6 8 10 12 14 16 18 20 22 24
Time (hours)
0µg/mL
1µg/mL
2µg/mL
4µg/mL
8µg/mL
16µg/mL
32µg/mL
64µg/mL
128µg/mL
256µg/mL

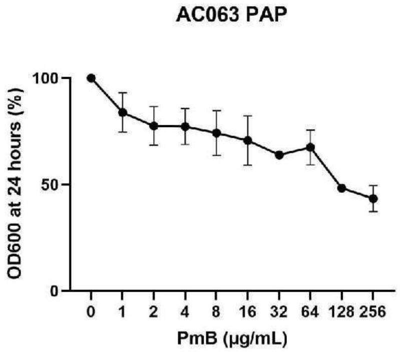

AC063 PAP
OD600 at 24 hours (%)
100
50
0
0 1 2 4 8 16 32 64 128 256
PmB (µg/mL)

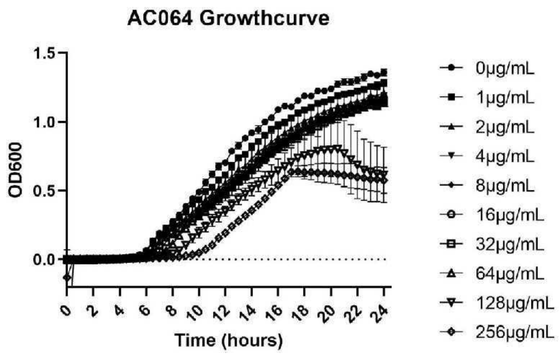
AC064 Growthcurve
1.5
1.0
0.5
0.0
OD600
0
2
4
6
8
10
12
14
16
18
20
22
24
Time (hours)
0μg/mL
1μg/mL
2μg/mL
4μg/mL
8μg/mL
16μg/mL
32μg/mL
64μg/mL
128μg/mL
256μg/mL

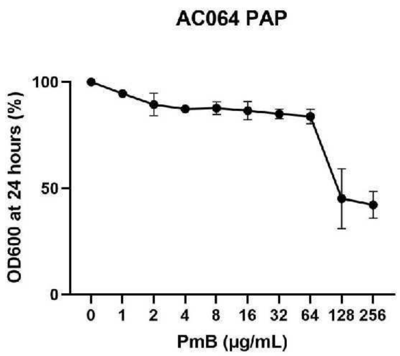
AC064 PAP
100
50
0
OD600 at 24 hours (%)
0
1
2
4
8
16
32
64
128
256
PmB (μg/mL)

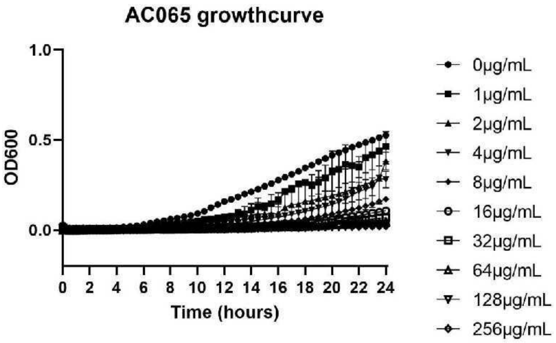
AC065 growthcurve
1.0
0.5
0.0
OD600
0 2 4 6 8 10 12 14 16 18 20 22 24
Time (hours)
0μg/mL
1μg/mL
2μg/mL
4μg/mL
8μg/mL
16μg/mL
32μg/mL
64μg/mL
128μg/mL
256μg/mL

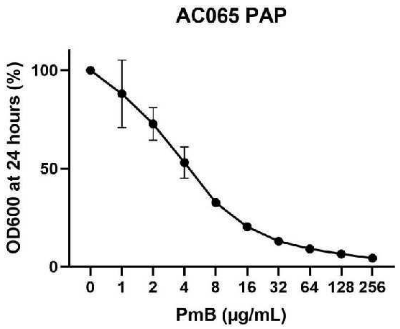
AC065 PAP
OD600 at 24 hours (%)
100
50
0
0 1 2 4 8 16 32 64 128 256
PmB (μg/mL)

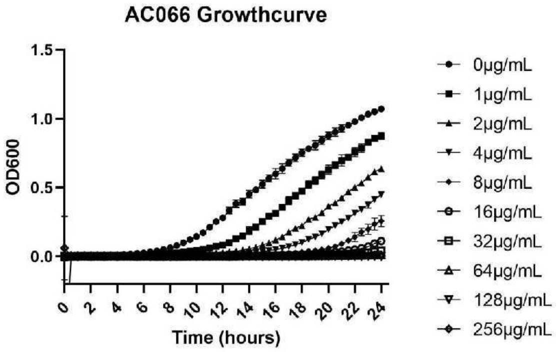
AC066 Growthcurve
1.5
1.0
0.5
0.0
OD600
0
2
4
6
8
10
12
14
16
18
20
22
24
Time (hours)
0μg/mL
1μg/mL
2μg/mL
4μg/mL
8μg/mL
16μg/mL
32μg/mL
64μg/mL
128μg/mL
256μg/mL

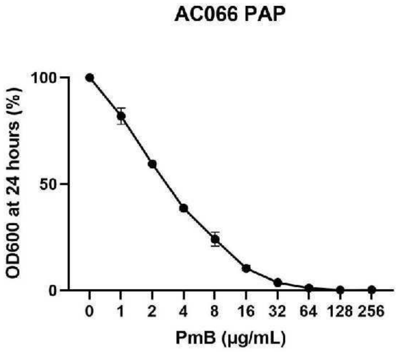
AC066 PAP
100
50
0
OD600 at 24 hours (%)
0
1
2
4
8
16
32
64
128
256
PmB (μg/mL)

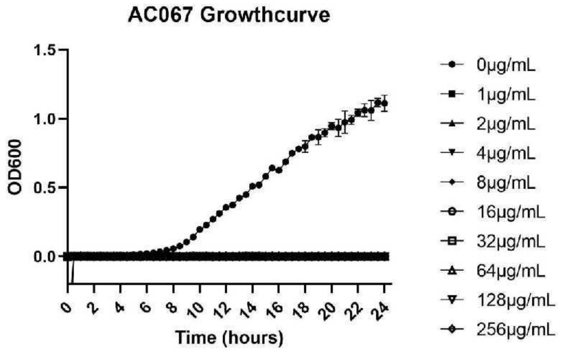
AC067 Growthcurve
1.5
1.0
0.5
0.0
OD600
0
2
4
6
8
10
12
14
16
18
20
22
24
Time (hours)
0μg/mL
1μg/mL
2μg/mL
4μg/mL
8μg/mL
16μg/mL
32μg/mL
64μg/mL
128μg/mL
256μg/mL

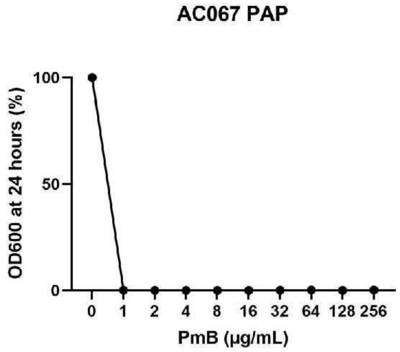
AC067 PAP
100
50
0
OD600 at 24 hours (%)
0
1
2
4
8
16
32
64
128
256
PmB (μg/mL)

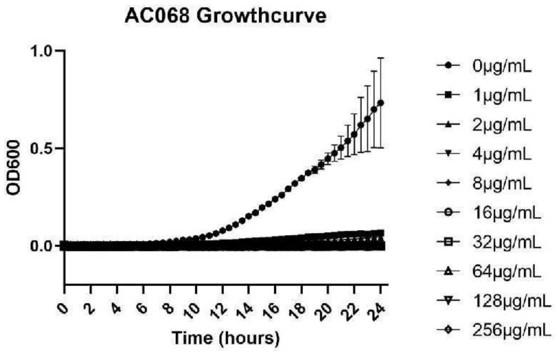
AC068 Growthcurve
1.0
0.5
0.0
OD600
0
2
4
6
8
10
12
14
16
18
20
22
24
Time (hours)
0µg/mL
1µg/mL
2µg/mL
4µg/mL
8µg/mL
16µg/mL
32µg/mL
64µg/mL
128µg/mL
256µg/mL

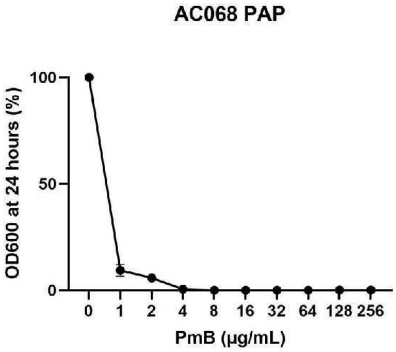
AC068 PAP
100
50
0
OD600 at 24 hours (%)
0
1
2
4
8
16
32
64
128
256
PmB (µg/mL)

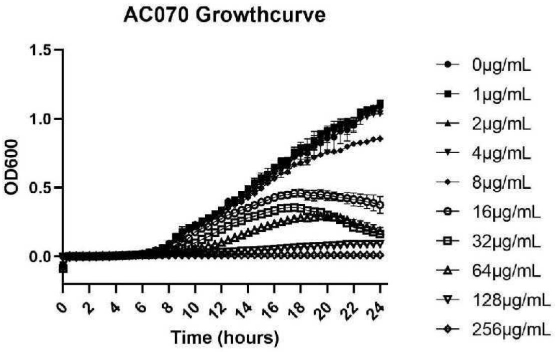
AC070 Growthcurve
1.5
1.0
0.5
0.0
OD600
0
2
4
6
8
10
12
14
16
18
20
22
24
Time (hours)
0μg/mL
1μg/mL
2μg/mL
4μg/mL
8μg/mL
16μg/mL
32μg/mL
64μg/mL
128μg/mL
256μg/mL

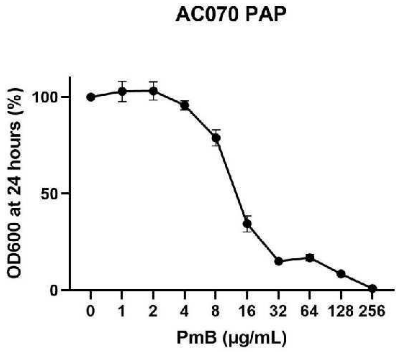
AC070 PAP
100
50
0
OD600 at 24 hours (%)
0
1
2
4
8
16
32
64
128
256
PmB (μg/mL)

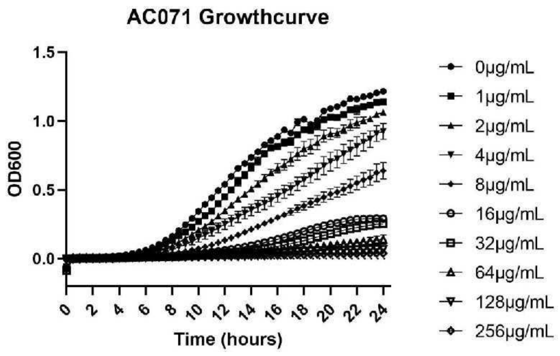
AC071 Growthcurve
1.5
1.0
0.5
0.0
OD600
0
2
4
6
8
10
12
14
16
18
20
22
24
Time (hours)
0µg/mL
1µg/mL
2µg/mL
4µg/mL
8µg/mL
16µg/mL
32µg/mL
64µg/mL
128µg/mL
256µg/mL

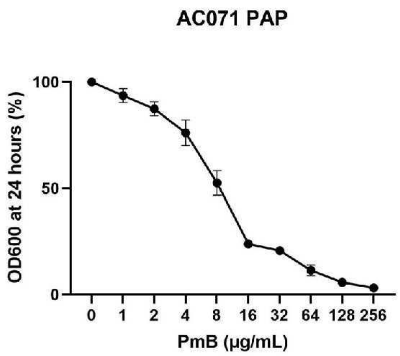
AC071 PAP
100
50
0
OD600 at 24 hours (%)
0
1
2
4
8
16
32
64
128
256
PmB (µg/mL)

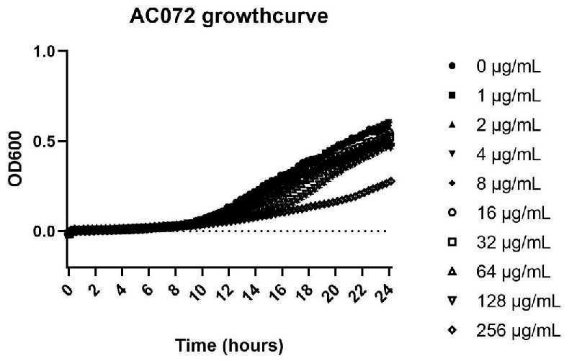
AC072 growthcurve
1.0
0.5
0.0
OD600
0 2 4 6 8 10 12 14 16 18 20 22 24
Time (hours)
0 µg/mL
1 µg/mL
2 µg/mL
4 µg/mL
8 µg/mL
16 µg/mL
32 µg/mL
64 µg/mL
128 µg/mL
256 µg/mL

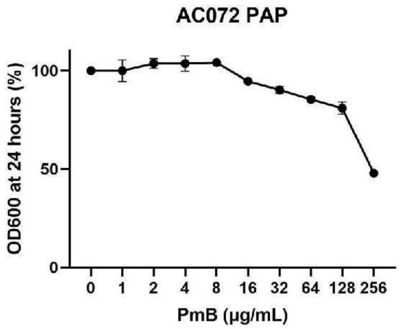
AC072 PAP
100
50
0
OD600 at 24 hours (%)
0 1 2 4 8 16 32 64 128 256
PmB (µg/mL)

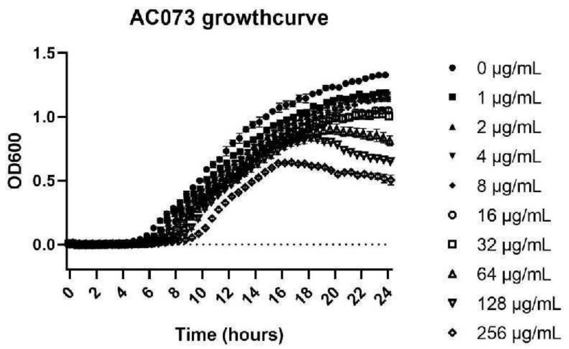
AC073 growthcurve
1.5
1.0
0.5
0.0
OD600
0
2
4
6
8
10
12
14
16
18
20
22
24
Time (hours)
0 µg/mL
1 µg/mL
2 µg/mL
4 µg/mL
8 µg/mL
16 µg/mL
32 µg/mL
64 µg/mL
128 µg/mL
256 µg/mL

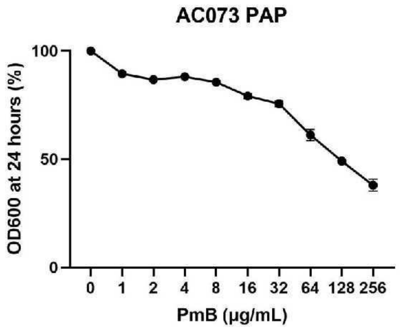
AC073 PAP
100
50
0
OD600 at 24 hours (%)
0
1
2
4
8
16
32
64
128
256
PmB (µg/mL)

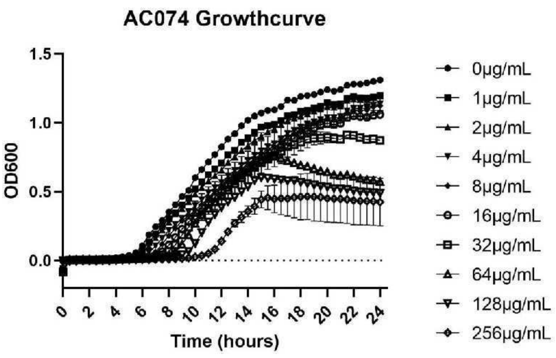
AC074 Growthcurve
1.5
1.0
0.5
0.0
OD600
0
2
4
6
8
10
12
14
16
18
20
22
24
Time (hours)
0μg/mL
1μg/mL
2μg/mL
4μg/mL
8μg/mL
16μg/mL
32μg/mL
64μg/mL
128μg/mL
256μg/mL

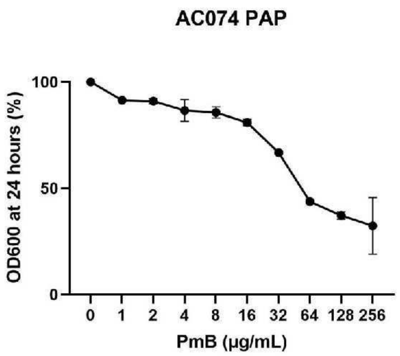
AC074 PAP
100
50
0
OD600 at 24 hours (%)
0
1
2
4
8
16
32
64
128
256
PmB (μg/mL)

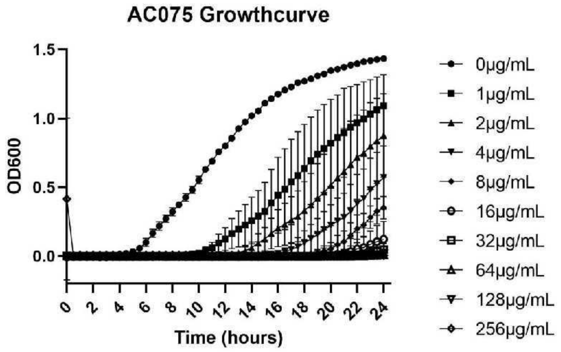
AC075 Growthcurve
1.5
1.0
0.5
0.0
OD600
0
2
4
6
8
10
12
14
16
18
20
22
24
Time (hours)
0μg/mL
1μg/mL
2μg/mL
4μg/mL
8μg/mL
16μg/mL
32μg/mL
64μg/mL
128μg/mL
256μg/mL

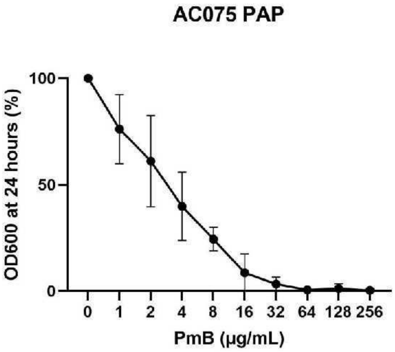
AC075 PAP
100
50
0
OD600 at 24 hours (%)
0
1
2
4
8
16
32
64
128
256
PmB (μg/mL)

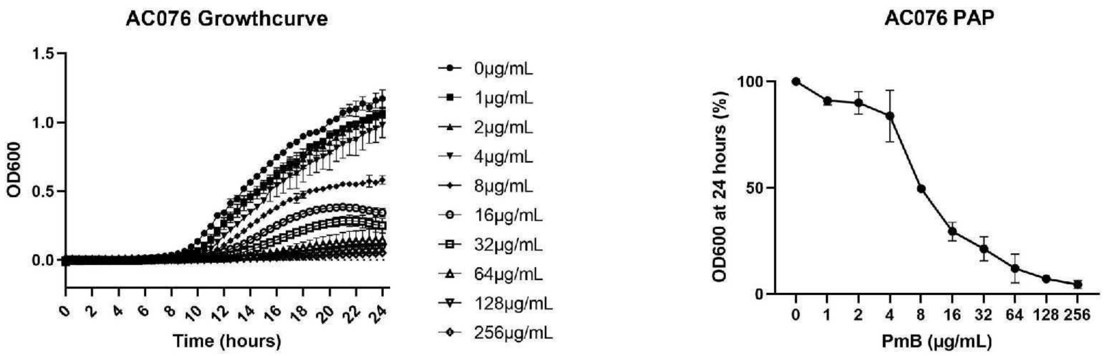
AC076 Growthcurve
OD600
1.5
1.0
0.5
0.0
0
2
4
6
8
10
12
14
16
18
20
22
24
Time (hours)
0µg/mL
1µg/mL
2µg/mL
4µg/mL
8µg/mL
16µg/mL
32µg/mL
64µg/mL
128µg/mL
256µg/mL
AC076 PAP
OD600 at 24 hours (%)
100
50
0
0
1
2
4
8
16
32
64
128
256
PmB (µg/mL)

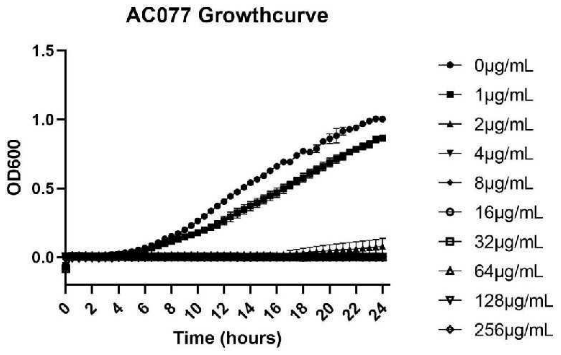
AC077 Growthcurve
1.5
1.0
0.5
0.0
OD600
0
2
4
6
8
10
12
14
16
18
20
22
24
Time (hours)
0μg/mL
1μg/mL
2μg/mL
4μg/mL
8μg/mL
16μg/mL
32μg/mL
64μg/mL
128μg/mL
256μg/mL

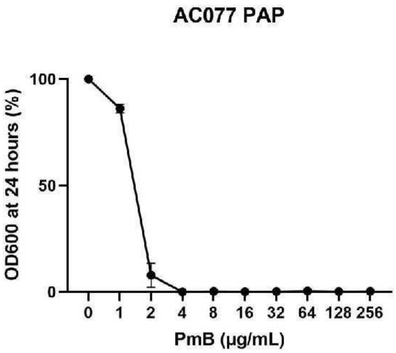
AC077 PAP
100
50
0
OD600 at 24 hours (%)
0
1
2
4
8
16
32
64
128
256
PmB (μg/mL)

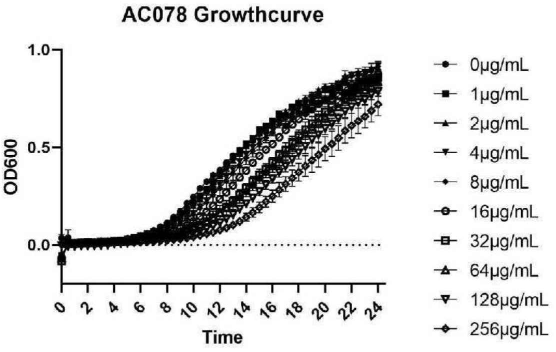
AC078 Growthcurve
1.0
0.5
0.0
OD600
0
2
4
6
8
10
12
14
16
18
20
22
24
Time
0μg/mL
1μg/mL
2μg/mL
4μg/mL
8μg/mL
16μg/mL
32μg/mL
64μg/mL
128μg/mL
256μg/mL

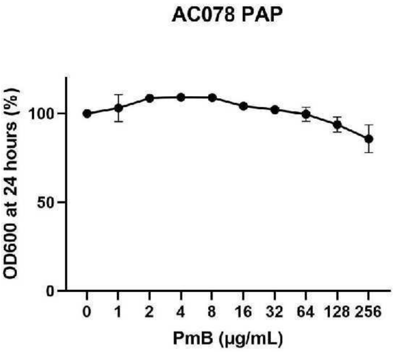
AC078 PAP
100
50
0
OD600 at 24 hours (%)
0
1
2
4
8
16
32
64
128
256
PmB (μg/mL)

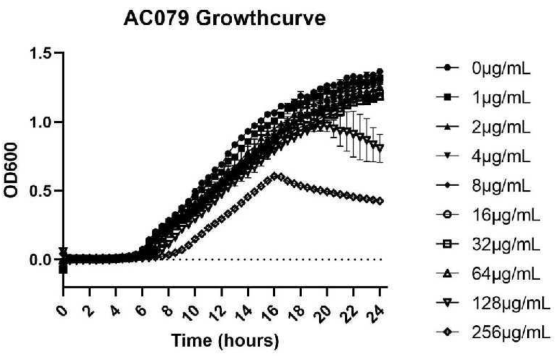
AC079 Growthcurve
1.5
1.0
0.5
0.0
OD600
0
2
4
6
8
10
12
14
16
18
20
22
24
Time (hours)
0μg/mL
1μg/mL
2μg/mL
4μg/mL
8μg/mL
16μg/mL
32μg/mL
64μg/mL
128μg/mL
256μg/mL

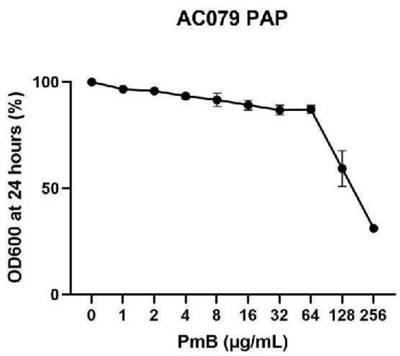
AC079 PAP
100
50
0
OD600 at 24 hours (%)
0
1
2
4
8
16
32
64
128
256
PmB (μg/mL)

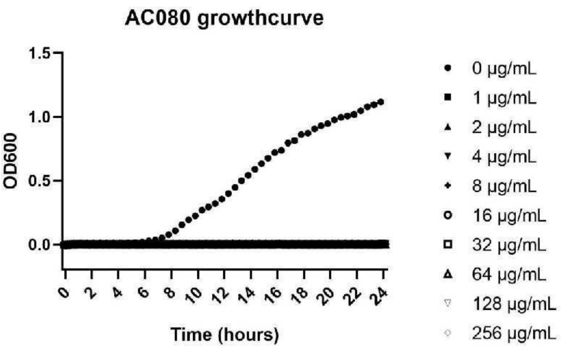
AC080 growthcurve
1.5
1.0
0.5
0.0
OD600
0 2 4 6 8 10 12 14 16 18 20 22 24
Time (hours)
0 µg/mL
1 µg/mL
2 µg/mL
4 µg/mL
8 µg/mL
16 µg/mL
32 µg/mL
64 µg/mL
128 µg/mL
256 µg/mL

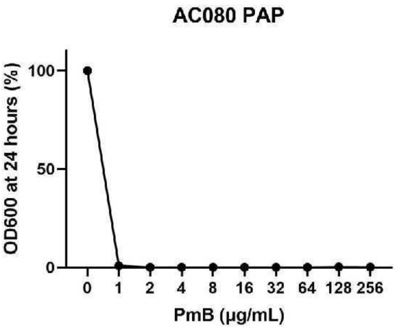
AC080 PAP
100
50
0
OD600 at 24 hours (%)
0 1 2 4 8 16 32 64 128 256
PmB (µg/mL)

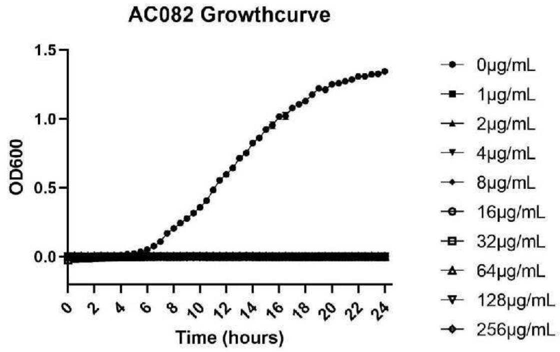
AC082 Growthcurve
1.5
1.0
0.5
0.0
OD600
0
2
4
6
8
10
12
14
16
18
20
22
24
Time (hours)
0μg/mL
1μg/mL
2μg/mL
4μg/mL
8μg/mL
16μg/mL
32μg/mL
64μg/mL
128μg/mL
256μg/mL

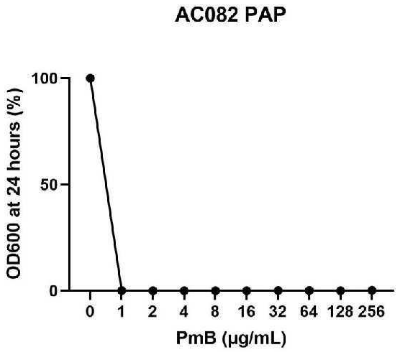
AC082 PAP
100
50
0
OD600 at 24 hours (%)
0
1
2
4
8
16
32
64
128
256
PmB (μg/mL)

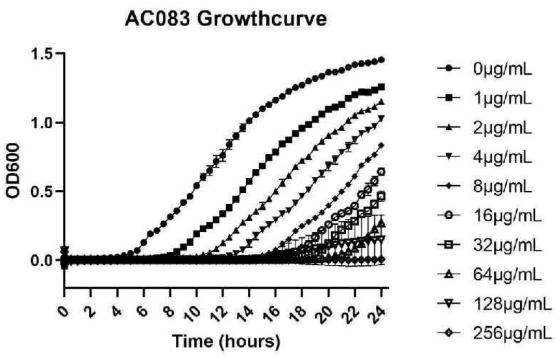
AC083 Growthcurve
OD600
Time (hours)
0µg/mL
1µg/mL
2µg/mL
4µg/mL
8µg/mL
16µg/mL
32µg/mL
64µg/mL
128µg/mL
256µg/mL

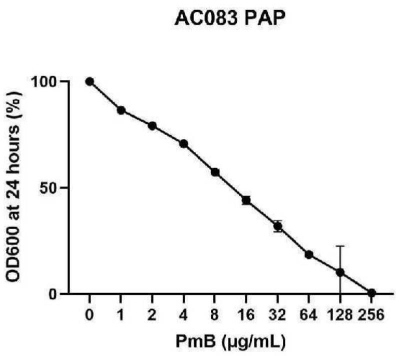
AC083 PAP
OD600 at 24 hours (%)
PmB (µg/mL)

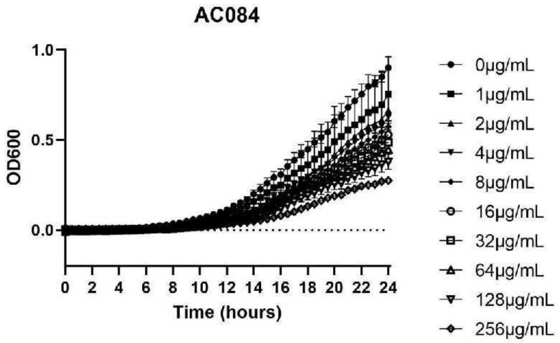
AC084
1.0
0.5
0.0
OD600
0 2 4 6 8 10 12 14 16 18 20 22 24
Time (hours)
0μg/mL
1μg/mL
2μg/mL
4μg/mL
8μg/mL
16μg/mL
32μg/mL
64μg/mL
128μg/mL
256μg/mL

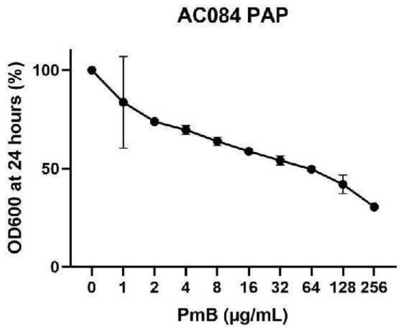
AC084 PAP
100
50
0
OD600 at 24 hours (%)
0 1 2 4 8 16 32 64 128 256
PmB (μg/mL)

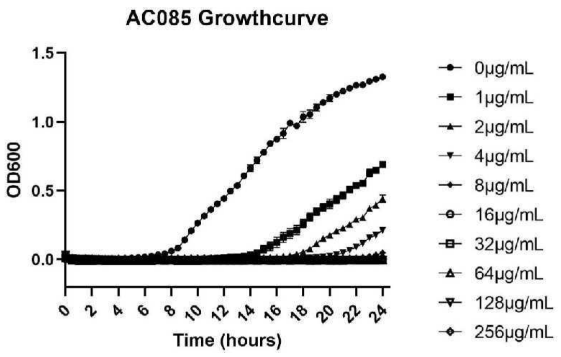
AC085 Growthcurve
1.5
1.0
0.5
0.0
OD600
0
2
4
6
8
10
12
14
16
18
20
22
24
Time (hours)
0μg/mL
1μg/mL
2μg/mL
4μg/mL
8μg/mL
16μg/mL
32μg/mL
64μg/mL
128μg/mL
256μg/mL

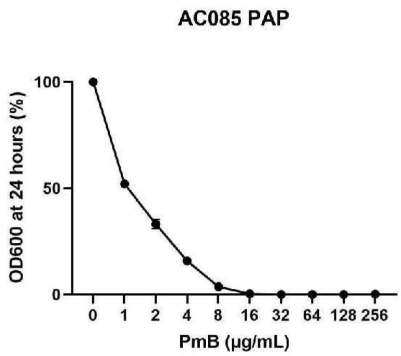
AC085 PAP
100
50
0
OD600 at 24 hours (%)
0
1
2
4
8
16
32
64
128
256
PmB (μg/mL)

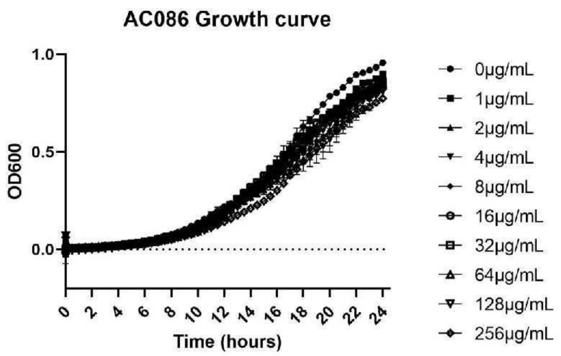

AC086 Growth curve
1.0
0.5
0.0
OD600
0
2
4
6
8
10
12
14
16
18
20
22
24
Time (hours)
0μg/mL
1μg/mL
2μg/mL
4μg/mL
8μg/mL
16μg/mL
32μg/mL
64μg/mL
128μg/mL
256μg/mL

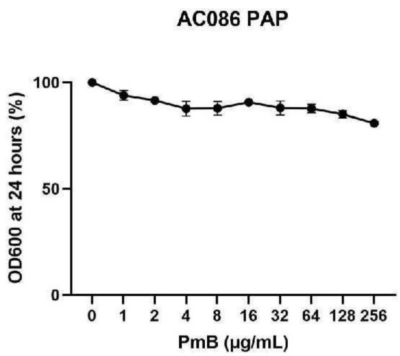

AC086 PAP
100
50
0
OD600 at 24 hours (%)
0
1
2
4
8
16
32
64
128
256
PmB (μg/mL)

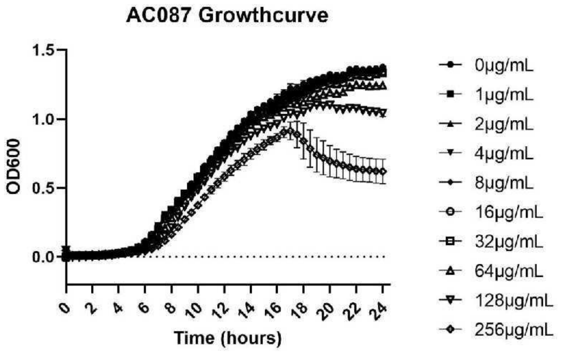
AC087 Growthcurve
1.5
1.0
0.5
0.0
OD600
0
2
4
6
8
10
12
14
16
18
20
22
24
Time (hours)
0μg/mL
1μg/mL
2μg/mL
4μg/mL
8μg/mL
16μg/mL
32μg/mL
64μg/mL
128μg/mL
256μg/mL

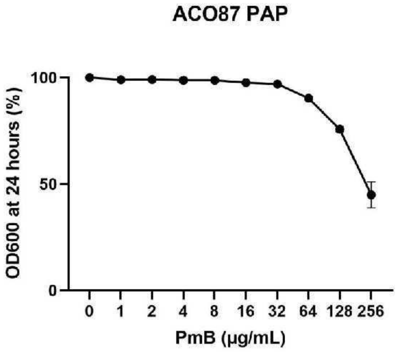
ACO87 PAP
100
50
0
OD600 at 24 hours (%)
0
1
2
4
8
16
32
64
128
256
PmB (μg/mL)

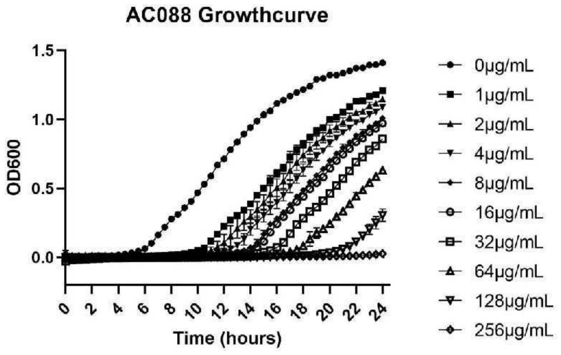
AC088 Growthcurve
OD600
1.5
1.0
0.5
0.0
0
2
4
6
8
10
12
14
16
18
20
22
24
Time (hours)
0μg/mL
1μg/mL
2μg/mL
4μg/mL
8μg/mL
16μg/mL
32μg/mL
64μg/mL
128μg/mL
256μg/mL

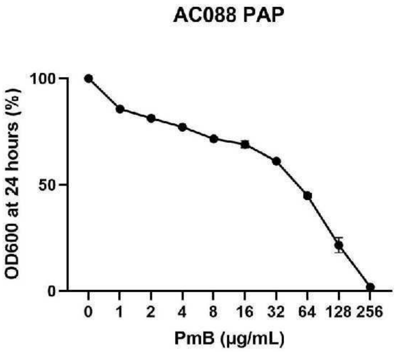
AC088 PAP
OD600 at 24 hours (%)
100
50
0
0
1
2
4
8
16
32
64
128
256
PmB (μg/mL)

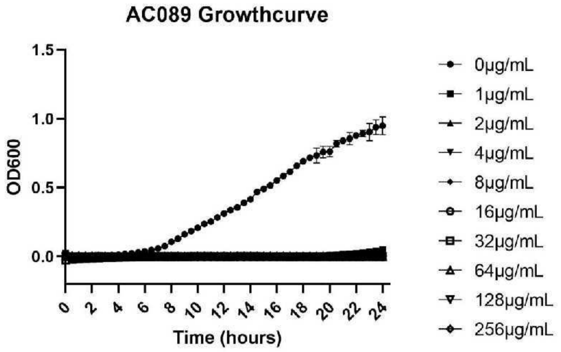
AC089 Growthcurve
1.5
1.0
0.5
0.0
OD600
0
2
4
6
8
10
12
14
16
18
20
22
24
Time (hours)
0μg/mL
1μg/mL
2μg/mL
4μg/mL
8μg/mL
16μg/mL
32μg/mL
64μg/mL
128μg/mL
256μg/mL

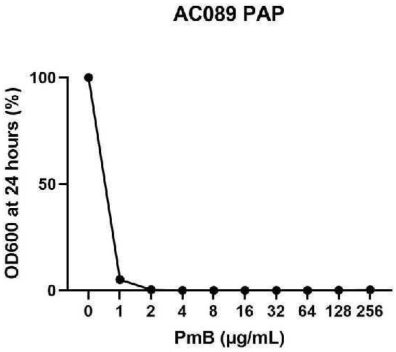
AC089 PAP
100
50
0
OD600 at 24 hours (%)
0
1
2
4
8
16
32
64
128
256
PmB (μg/mL)

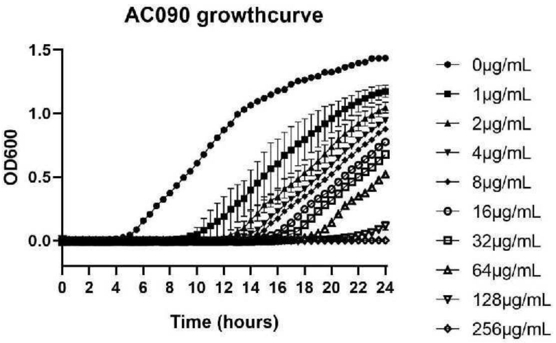

AC090 growthcurve
OD600
1.5
1.0
0.5
0.0
0 2 4 6 8 10 12 14 16 18 20 22 24
Time (hours)
0μg/mL
1μg/mL
2μg/mL
4μg/mL
8μg/mL
16μg/mL
32μg/mL
64μg/mL
128μg/mL
256μg/mL

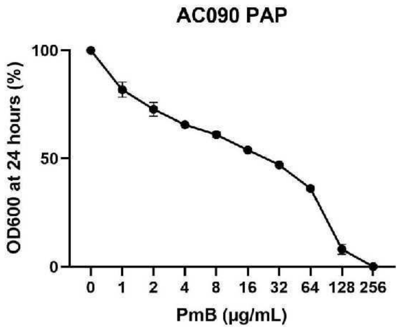

AC090 PAP
OD600 at 24 hours (%)
100
50
0
0 1 2 4 8 16 32 64 128 256
PmB (μg/mL)

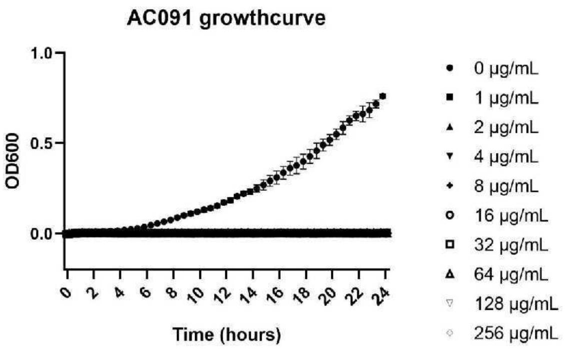
AC091 growthcurve
1.0
0.5
0.0
OD600
0
2
4
6
8
10
12
14
16
18
20
22
24
Time (hours)
0 μg/mL
1 μg/mL
2 μg/mL
4 μg/mL
8 μg/mL
16 μg/mL
32 μg/mL
64 μg/mL
128 μg/mL
256 μg/mL

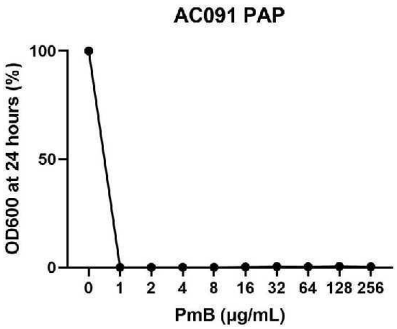
AC091 PAP
100
50
0
OD600 at 24 hours (%)
0
1
2
4
8
16
32
64
128
256
PmB (μg/mL)

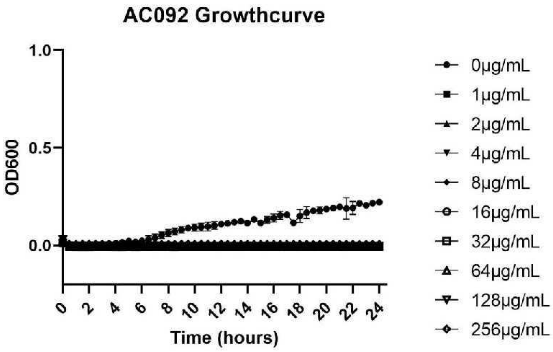
AC092 Growthcurve
1.0
0.5
0.0
OD600
0
2
4
6
8
10
12
14
16
18
20
22
24
Time (hours)
0μg/mL
1μg/mL
2μg/mL
4μg/mL
8μg/mL
16μg/mL
32μg/mL
64μg/mL
128μg/mL
256μg/mL

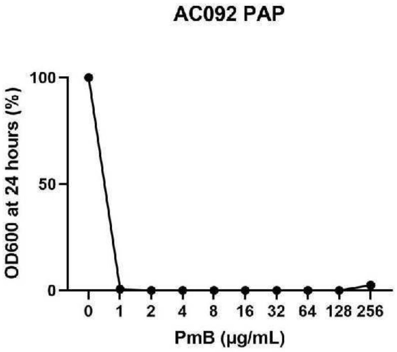
AC092 PAP
100
50
0
OD600 at 24 hours (%)
0
1
2
4
8
16
32
64
128
256
PmB (μg/mL)

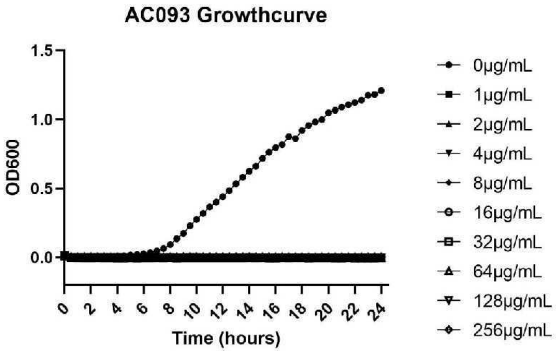
AC093 Growthcurve
1.5
1.0
0.5
0.0
OD600
0 2 4 6 8 10 12 14 16 18 20 22 24
Time (hours)
0µg/mL
1µg/mL
2µg/mL
4µg/mL
8µg/mL
16µg/mL
32µg/mL
64µg/mL
128µg/mL
256µg/mL

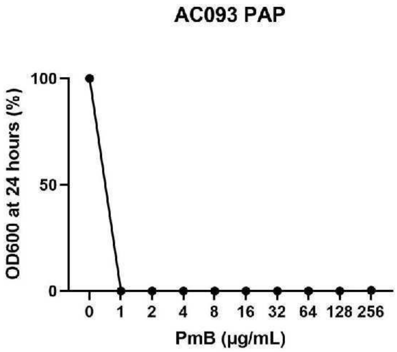
AC093 PAP
100
50
0
OD600 at 24 hours (%)
0 1 2 4 8 16 32 64 128 256
PmB (µg/mL)

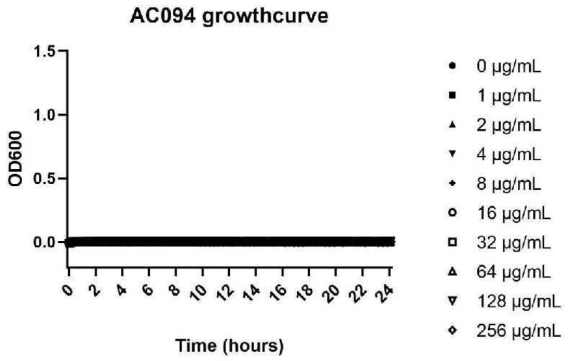

AC094 growthcurve
1.5
1.0
0.5
0.0
OD600
0
2
4
6
8
10
12
14
16
18
20
22
24
Time (hours)
0 µg/mL
1 µg/mL
2 µg/mL
4 µg/mL
8 µg/mL
16 µg/mL
32 µg/mL
64 µg/mL
128 µg/mL
256 µg/mL

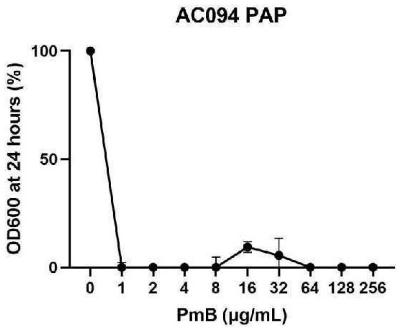

AC094 PAP
100
50
0
OD600 at 24 hours (%)
0
1
2
4
8
16
32
64
128
256
PmB (µg/mL)

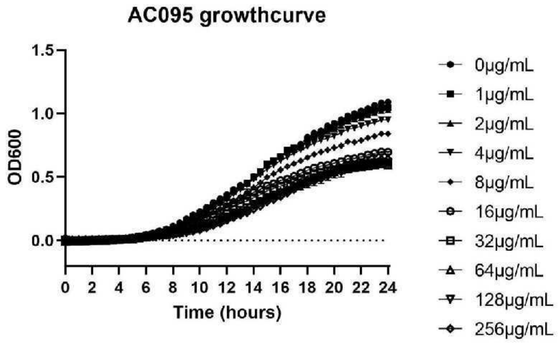
AC095 growthcurve
OD600
1.5
1.0
0.5
0.0
0 2 4 6 8 10 12 14 16 18 20 22 24
Time (hours)
0μg/mL
1μg/mL
2μg/mL
4μg/mL
8μg/mL
16μg/mL
32μg/mL
64μg/mL
128μg/mL
256μg/mL

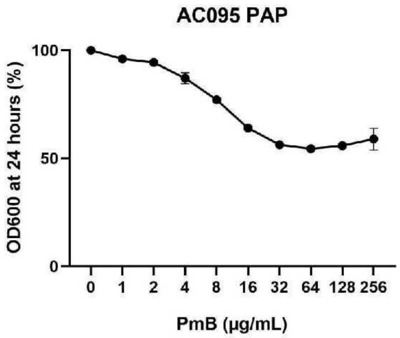
AC095 PAP
OD600 at 24 hours (%)
100
50
0
0 1 2 4 8 16 32 64 128 256
PmB (μg/mL)

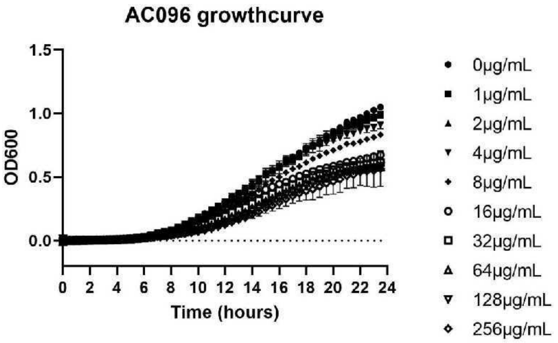

AC096 growthcurve
1.5
1.0
0.5
0.0
OD600
0 2 4 6 8 10 12 14 16 18 20 22 24
Time (hours)
0μg/mL
1μg/mL
2μg/mL
4μg/mL
8μg/mL
16μg/mL
32μg/mL
64μg/mL
128μg/mL
256μg/mL

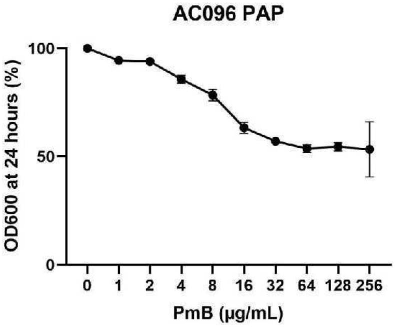

AC096 PAP
100
50
0
OD600 at 24 hours (%)
0 1 2 4 8 16 32 64 128 256
PmB (μg/mL)

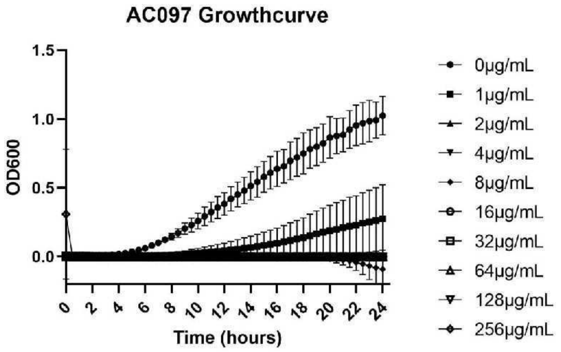
AC097 Growthcurve
1.5
1.0
0.5
0.0
OD600
0
2
4
6
8
10
12
14
16
18
20
22
24
Time (hours)
0μg/mL
1μg/mL
2μg/mL
4μg/mL
8μg/mL
16μg/mL
32μg/mL
64μg/mL
128μg/mL
256μg/mL

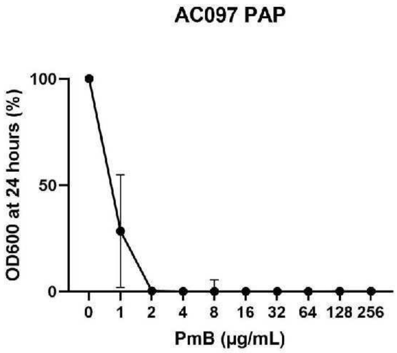
AC097 PAP
100
50
0
OD600 at 24 hours (%)
0
1
2
4
8
16
32
64
128
256
PmB (μg/mL)

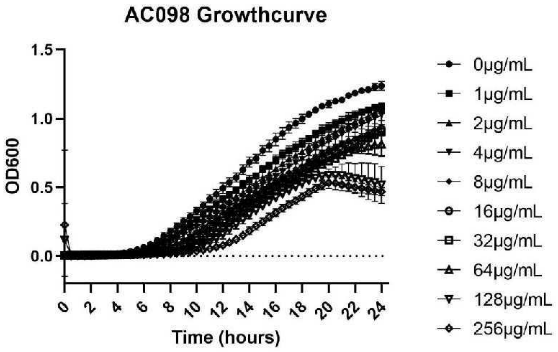
AC098 Growthcurve
1.5
1.0
0.5
0.0
OD600
0
2
4
6
8
10
12
14
16
18
20
22
24
Time (hours)
0µg/mL
1µg/mL
2µg/mL
4µg/mL
8µg/mL
16µg/mL
32µg/mL
64µg/mL
128µg/mL
256µg/mL

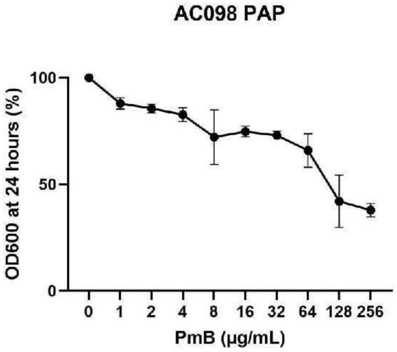
AC098 PAP
100
50
0
OD600 at 24 hours (%)
0
1
2
4
8
16
32
64
128
256
PmB (µg/mL)

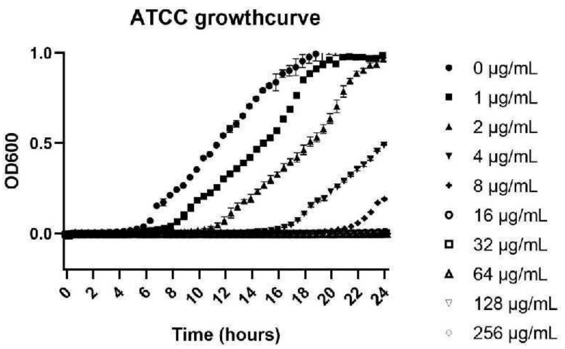
ATCC growthcurve
1.0
0.5
0.0
OD600
0
2
4
6
8
10
12
14
16
18
20
22
24
Time (hours)
0 μg/mL
1 μg/mL
2 μg/mL
4 μg/mL
8 μg/mL
16 μg/mL
32 μg/mL
64 μg/mL
128 μg/mL
256 μg/mL

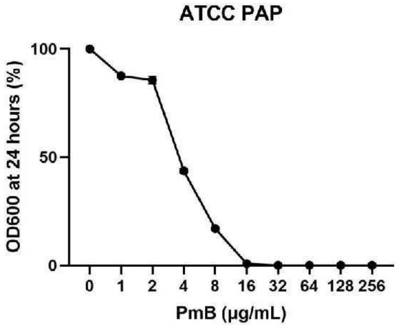
ATCC PAP
100
50
0
OD600 at 24 hours (%)
0
1
2
4
8
16
32
64
128
256
PmB (μg/mL)
